# Supplementary material for: Cobalt-catalyzed electrooxidative C-H/N-H [4+2] annulation with ethylene or ethyne
Source: Nat Commun. 2018 Feb 23;9:798. doi: 10.1038/s41467-018-03246-4 (PMC5824839; doi:10.1038/s41467-018-03246-4)
Supplement: Supplementary file 1 — Supplementary Information [file 41467_2018_3246_MOESM1_ESM.pdf]

Cobalt-catalyzed electrooxidative C-H/N-H [4+2] annulation with ethylene or ethyne

Tang et al.

Supplementary Methods

All glassware was oven dried at 110 ^{o}C for hours and cooled down under vacuum. **1a-1e**,[1] **1f-1h**,[2] **1i**,[3] **1j**,[4] **1k**[5] and **[D$_5$]-1a**[6] was prepared according to reported procedures. Unless otherwise noted, materials were obtained from commercial suppliers and used without further purification. The instrument for electrolysis is dual display potentiostat (DJS-292B) (made in China). Cyclic voltammograms were obtained on a CHI 605E potentiostat. All the electrolysis reactions were conducted in an H-type divided cell with ULTREX® AMI-7001-30 membrane (A corrosion-resistant base anion exchange membrane). The electrolytic cell was shown in Supplementary Figure 1. The anodic electrode was carbon cloth (15 mm×15 mm×0.36 mm) and cathodic electrode was nickel plate (15 mm×15 mm×1.0 mm). Thin layer chromatography (TLC) employed glass 0.25 mm silica gel plates. Flash chromatography columns were packed with 200-300 mesh silica gel in petroleum (bp. 60-90 ^{o}C). ^{1}H and ^{13}C NMR data were recorded with Bruker Advance III (400 MHz) spectrometers with tetramethylsilane as an internal standard. All chemical shifts (δ) are reported in ppm and coupling constants (*J*) in Hz. All chemical shifts were reported relative to tetramethylsilane (0 ppm for ^{1}H), $CDCl_3$ (77.0 ppm for ^{13}C) and DMSO-d_6 (2.50 ppm for ^{1}H, 39.52 ppm for ^{13}C), respectively. IR spectra were recorded on a NICOLET 5700 FTIR spectrometer. High resolution mass spectra (HRMS) were measured with a Waters Micromass GCT instrument and accurate masses were reported for the molecular hydrogen ion $(M+H)^+$ or $(M+Na)^+$.

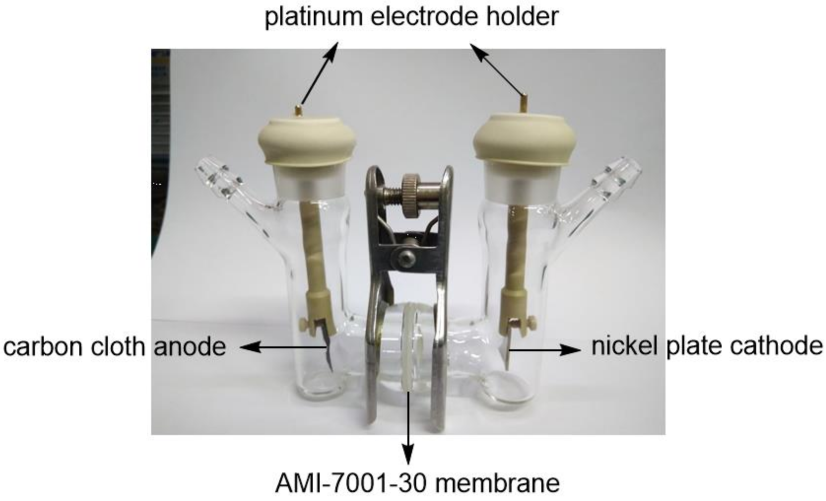

Supplementary Figure 1. The H-type divided cell for electrolysis.

Electrooxidative C-H/N-H [4+2] annulation with ethylene (Method A): The electrolysis was carried out in an oven-dried H-type divided cell equipped with two stir bars. Carbon cloth (15 mm×15 mm×0.36 mm) was used as the anode and nickel plate (15 mm×15 mm×1.0 mm) was used as the cathode. The two electrodes were separated by an ULTREX® AMI-7001-30 membrane. The anodic chamber was added with amide (0.20 mmol), Co(acac)$_2$ (0.030 mmol, 7.7 mg), NaOPiv•H_2O (0.30 mmol, 42.6 mg) and ^{n}Bu$_4$NBF$_4$ (0.60 mmol, 197.6 mg) while the cathodic chamber was added with NaOPiv•H_2O (0.30 mmol, 42.6 mg) and HOPiv (2.0 mmol, 204.3 mg). A balloon filled with ethylene (1 atm) was connected to the electrolysis system and purged for three times. Subsequently, CF_3CH_2OH (8.0 mL) and MeOH (8.0 mL) were added to the anodic chamber and cathodic chamber, respectively. Then the electrolysis system was stirred at a constant current of 4 mA at 70 ^oC for 4 h. When the reaction was finished, the reaction mixture of the anodic chamber was washed with water and extracted with diethyl ether (10 mL x 3). The organic layers were combined, dried over Na_2SO_4, and concentrated. The pure product was obtained by flash column chromatography on silica gel (petroleum: ethyl acetate = 1:1).

Electrooxidative C-H/N-H [4+2] annulation with ethyne (Method B): The electrolysis was carried out in an oven-dried H-type divided cell equipped with two stir bars. Carbon cloth (15 mm×15 mm×0.36 mm) was used as the anode and nickel plate (15 mm×15 mm×1.0 mm) was used as the cathode. The two electrodes were separated by an ULTREX® AMI-7001-30 membrane. The anodic chamber was added with amide (0.20 mmol), Co(acac)$_2$ (0.020 mmol, 5.1 mg), NaOPiv•H_2O (0.40 mmol, 56.9 mg) and ^{n}Bu$_4$NBF$_4$ (0.60 mmol, 197.6 mg) while the cathodic chamber was added with NaOPiv•H_2O (0.40 mmol, 56.9 mg) and HOPiv (2.0 mmol, 204.3 mg). A balloon filled with ethyne (1 atm) was connected to the electrolysis system and purged for three times. Subsequently, CF_3CH_2OH (8.0 mL) and MeOH (8.0 mL) were added to the anodic chamber and cathodic chamber, respectively. Then electrolysis system was stirred at a constant current of 5 mA at 70 ^oC for 3 h. When the reaction was finished, the reaction mixture of the anodic chamber was washed with water and extracted with diethyl ether (10 mL x 3). The organic layers were combined, dried over Na_2SO_4, and concentrated. The pure product was obtained by flash column chromatography on silica gel (petroleum: ethyl acetate = 1:1).

Procedure for the gram scale synthesis of 3aa: The electrolysis was carried out in an oven-dried H-type divided cell equipped with two stir bars. Carbon cloth (30 mm×30 mm×0.36 mm) electrodes were used both for anode and cathode. The two electrodes were separated by an ULTREX® AMI-7001-30 membrane. The anodic chamber was added with **1a** (5.0 mmol, 1.2 g), Co(acac)$_2$ (0.75 mmol, 0.19 g), NaOPiv•H_2O (7.5 mmol, 1.1 g) and ^{n}Bu$_4$NBF$_4$ (15 mmol, 4.9 g) while the cathodic chamber was added with NaOPiv•H_2O (7.5 mmol, 1.1 g) and HOPiv (50 mmol, 5.1 g). A balloon filled with ethylene (1 atm) was connected to the electrolysis system and purged for three times. Subsequently, CF_3CH_2OH (50 mL) and MeOH (50 mL) were added to the anodic chamber and cathodic chamber, respectively. Then electrolysis system was stirred at a constant current of 30 mA at 70 ^oC for 13 h. The reaction mixture of the anodic chamber was washed with water and extracted with diethyl ether (10 mL x 3). The organic layers were combined, dried over Na_2SO_4, and concentrated. The pure product was obtained by flash column chromatography on silica gel (petroleum: ethyl acetate = 1:1). White solid was obtained in 66% isolated yield (0.90 g).

Method of the kinetic isotope effect experiment of parallel reactions with 1a and [D$_5$]-1a: The electrolysis was carried out in an oven-dried H-type divided cell equipped with two stir bars. Carbon cloth (15 mm×15 mm×0.36 mm) was used as the anode and nickel plate (15 mm×15 mm×1.0 mm) was used as the cathode. The two electrodes were separated by an ULTREX® AMI-7001-30 membrane. The anodic chamber was added with **1a** (0.20 mmol, 49.6 mg), Co(acac)$_2$ (0.030 mmol, 7.7 mg), NaOPiv•H_2O (0.30 mmol, 42.6 mg) and ^{n}Bu$_4$NBF$_4$ (0.60 mmol, 197.6 mg) while the cathodic chamber was added with NaOPiv•H_2O (0.30 mmol, 42.6 mg) and HOPiv (2.0 mmol, 204.3 mg). Diphenyl (0.13 mol, 20.0 mg) was added as an internal standard. A balloon filled with ethylene (1 atm) was connected to the electrolysis system and purged for three times. A balloon filled with ethylene (1 atm) was connected to the electrolysis system and purged for three times. Subsequently, CF_3CH_2OH (8.0 mL) and MeOH (8.0 mL) were added to the anodic chamber and cathodic chamber, respectively. Then the electrolysis system was stirred at a constant current of 4 mA at 70 ^oC. 0.1 mL solution were taken out from the anode chamber via syringe at designated time interval (5, 10, 15, 20, 25, and 30 min). The samples were quenched by water and then analyzed by GC analysis. By using above procedure, the similar sets of experiments were conducted by using **[D$_5$]-1a** instead of **1a**. The KIE value for the C-H bond cleavage was k_H/k_D = 0.9 (See Supplementary Figure S2).

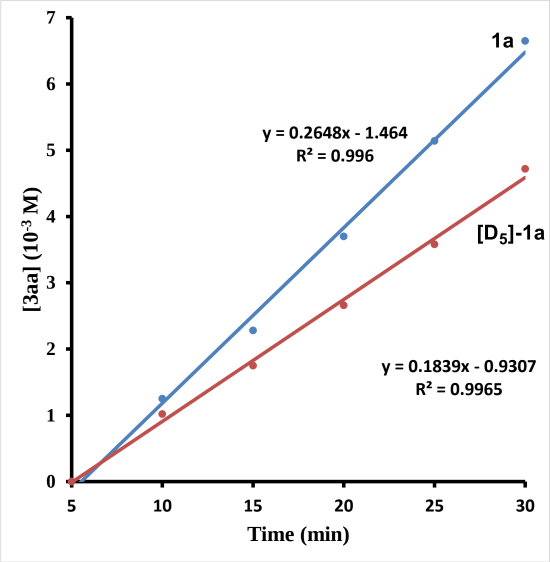

Supplementary Figure 2. The initial reaction rate of **1a** and **[D$_5$]-1a**

General procedure for kinetic study: The electrolysis was carried out in an oven-dried H-type divided cell equipped with two stir bars. Carbon cloth (15 mm×15 mm×0.36 mm) was used as the anode and nickel plate (15 mm×15 mm×1.0 mm) was used as the cathode. The two electrodes were separated by an ULTREX® AMI-7001-30 membrane. The anodic chamber was added with **1a** (0.20 mmol, 49.6 mg), Co(acac)$_2$ (0.030 mmol, 7.7 mg), NaOPiv•H_2O (0.30 mmol, 42.6 mg) and ^{n}Bu$_4$NBF$_4$ (0.60 mmol, 197.6 mg) while the cathodic chamber was added with NaOPiv•H_2O (0.30 mmol, 42.6 mg) and HOPiv (2.0 mmol, 204.3 mg). Diphenyl (0.13 mol, 20.0 mg) was added as an internal standard. Diphenyl (0.13 mol, 20.0 mg) was added as an internal standard. A balloon filled with ethylene (1 atm) was connected to the electrolysis system and purged for three times. A balloon filled with ethylene (1 atm) was connected to the electrolysis system and purged for three times. Subsequently, CF_3CH_2OH (8.0 mL) and MeOH (8.0 mL) were added to the anodic chamber and cathodic chamber, respectively. Then the electrolysis system was stirred at a constant current of 5 mA at 70 ^{o}C. 0.1 mL solution were taken out from the anode chamber via syringe at designated time interval (5, 10, 15, 20, 25, and 30 min; 4, 8, 12, 16, and 20 min). By using above procedure, the similar sets of experiments were conducted by using different concentration of **1a**, NaOPiv•H_2O or Co(acac)$_2$. Moreover, the similar sets of experiments were also conducted under different constant current. Only one parameter was changed from the general procedure in one reaction.

General procedure for cyclic voltammetry (CV): Cyclic voltammetry was performed in a three-electrode cell connected to a schlenk line under nitrogen at room temperature. The working electrode was a steady glassy carbon disk electrode, the counter electrode was a platinum wire. The reference was a Ag/AgCl electrode submerged in saturated aqueous KCl solution and was separated from reaction by a salt bridge. 10 mL of CF_3CH_2OH containing 0.06 M $^n Bu_4NBF_4$ were poured into the electrochemical cell in all experiments. The scan rate is 0.1 V/s, ranging from 0 V to 2.0 V.

Procedure for potential controlled electrolysis: The electrolysis was carried out in an oven-dried H-type divided cell equipped with two stir bars. Carbon cloth (15 mm×15 mm×0.36 mm) was used as the anode and nickel plate (15 mm×15 mm×1.0 mm) was used as the cathode. The two electrodes were separated by an ULTREX® AMI-7001-30 membrane. Different from the current controlled electrolysis, a reference electrode (Ag/AgCl) was place in the anodic chamber near the anode. The anodic chamber was added with amide (0.20 mmol), $Co(acac)_2$ (0.030 mmol, 7.7 mg), NaOPiv•H_2O (0.30 mmol, 42.6 mg) and $^n Bu_4NBF_4$ (0.60 mmol, 197.6 mg) while the cathodic chamber was added with NaOPiv•H_2O (0.30 mmol, 42.6 mg) and HOPiv (2.0 mmol, 204.3 mg). A balloon filled with ethylene (1 atm) was connected to the electrolysis system and purged for three times. Subsequently, CF_3CH_2OH (8.0 mL) and MeOH (8.0 mL) were added to the anodic chamber and cathodic chamber, respectively. Then the electrolysis system was stirred at at a controlled potential of 1.13 V (vs Ag/AgCl) and stopped until complete consumption of **1a**. The reaction mixture of the anodic chamber was washed with water and extracted with diethyl ether (10 mL x 3). The organic layers were combined, dried over Na_2SO_4, and concentrated. The pure product was obtained by flash column chromatography on silica gel (petroleum: ethyl acetate = 1:1).

Selected reaction results with substituted alkenes and alkynes

To further demonstrate the applicability of the electrooxidative annulation reaction, substituted alkenes and alkynes were also tried as substrates (Supplementary Figure 3). Both aryl and alkyl alkenes were found to be suitable in the [4+2] annulation reaction (**3ac** and **3ad**). Moreover, cyclic alkene could also generate a bicyclic compound in 58% yield (**3ae**). The reaction demonstrated good regioselectivity. Notably, (*E*)-prop-1-en-1-ylbenzene could lead to the formation of a single regioisomer in 50% yield. In addition, both terminal and internal alkynes were effective substrates under modified electrolytic conditions, affording the cyclization products in moderate to good yields (**3af-3ah**). The high regioselectivity observed in this reaction system was likely to be a result of the steric effect in the alkyne/alkene insertion step.

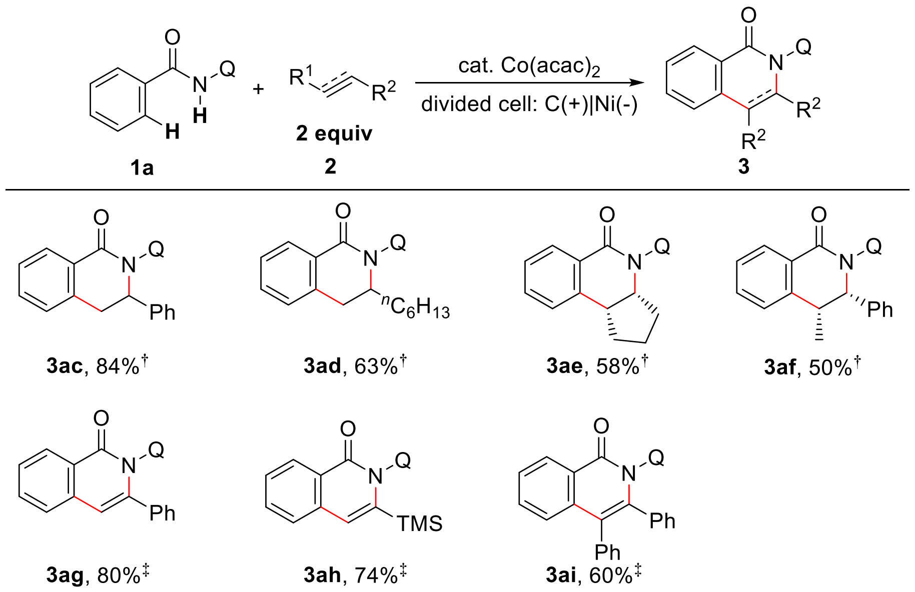

Supplementary Figure 3. Electrooxidative C-H/N-H [4+2] annulation with substituted alkenes or alkynes. † Reaction conditions: **1a** (0.20 mmol), **2** (2.0 equiv), $Co(acac)_2$ (15 mol%), NaOPiv•H_2O (1.5 equiv), $^{n}Bu_4NBF_4$ (3.0 equiv), CF_3CH_2OH (8.0 mL) [anode], and NaOPiv•H_2O (1.5 equiv), HOPiv (10 equiv), MeOH (8.0 mL) [cathode] in and H-type divided cell with carbon cloth anode, nickel plate cathode and a AMI-7001-30 membrane, constant current = 4 mA (J_{anode} = 1.78 mA/cm^2), 70 °C, 4 h (3.0 F/mol). Isolated yields are shown. ‡ Reaction conditions: **1a** (0.20 mmol), **2** (2.0 equiv), $Co(acac)_2$ (10 mol%), NaOPiv•H_2O (2.0 equiv), $^{n}Bu_4NBF_4$ (3.0 equiv), CF_3CH_2OH (8.0 mL) [anode], and NaOPiv•H_2O (2.0 equiv), HOPiv (10 equiv), MeOH (8.0 mL) [cathode] in and H-type divided cell with carbon cloth anode, nickel plate cathode and a AMI-7001-30 membrane, constant current = 5 mA (J_{anode} = 2.22 mA/cm^2), 70 °C, 3 h (2.80 F/mol). Isolated yields are shown.

Detail descriptions for products

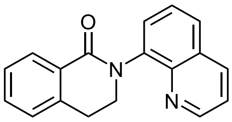

2-(Quinolin-8-yl)-3,4-dihydroisoquinolin-1(2H)-one (3aa):[3] white solid was obtained in 89% isolated yield following Method A. ^{1}H NMR (400 MHz, DMSO-d_6) δ 8.90 (dd, *J* = 4.1, 1.6 Hz, 1H), 8.44 (dd, *J* = 8.3, 1.5 Hz, 1H), 8.00 (d, *J* = 8.2 Hz, 1H), 7.94 (d, *J* = 7.7 Hz, 1H), 7.79 (dd, *J* = 7.3, 1.1 Hz, 1H), 7.68 (t, *J* = 7.7 Hz, 1H), 7.60 – 7.51 (m, 2H), 7.41 (t, *J* = 7.9 Hz, 2H), 4.28 – 3.69 (m, 2H), 3.27 – 3.17 (m, 2H). ^{13}C NMR (101 MHz, DMSO-d_6) δ 163.83, 150.70, 143.62, 140.77, 139.41, 136.65, 132.07, 129.56, 129.17, 129.11, 127.90, 127.78, 127.59, 126.93, 126.61, 121.89, 49.89, 28.10.

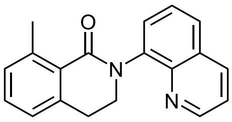

8-Methyl-2-(quinolin-8-yl)-3,4-dihydroisoquinolin-1(2H)-one (3ba): pale yellow solid was obtained in 85% isolated yield following Method A. ^{1}H NMR (400 MHz, $CDCl_3$) δ 8.89 (dd, *J* = 4.2, 1.7 Hz, 1H), 8.17 (dd, *J* = 8.3, 1.7 Hz, 1H), 7.80 (dd, *J* = 8.2, 1.3 Hz, 1H), 7.76 (dd, *J* = 7.3, 1.4 Hz, 1H), 7.61 – 7.56 (m, 1H), 7.39 (dd, *J* = 8.3, 4.2 Hz, 1H), 7.31 (t, *J* = 7.6 Hz, 1H), 7.16 (d, *J* = 7.6 Hz, 1H), 7.11 (d, *J* = 7.5 Hz, 1H), 4.37 – 3.64 (m, 2H), 3.43 – 3.07 (m, 2H), 2.70 (s, 3H). ^{13}C NMR (101 MHz, $CDCl_3$) δ 165.53, 150.27, 144.04, 141.38, 140.80, 140.40, 136.15, 130.79, 129.55, 128.99, 128.09, 127.42, 126.27, 124.93, 121.33, 49.93, 30.06, 22.49. IR (film): 3058, 2921, 2848, 1650, 1591, 1469, 1425, 1309, 1238, 1160, 1118, 1029, 985, 950, 877, 827, 790, 767, 692, 619, 566 cm^{-1}. HRMS calcd. for $C_{19}H_{16}N_2O$ $[M+Na]^+$: 311.1155; found: 311.1153.

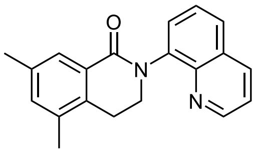

5,7-Dimethyl-2-(quinolin-8-yl)-3,4-dihydroisoquinolin-1(2H)-one (3ca): pale yellow solid was obtained in 80% isolated yield following Method A. ^{1}H NMR (400 MHz, $CDCl_3$) δ 8.87 (d, *J* = 2.7 Hz, 1H), 8.18 – 8.12 (m, 1H), 7.89 (s, 1H), 7.81 – 7.72 (m, 2H), 7.57 (t, *J* = 7.8 Hz, 1H), 7.37 (dd, *J* = 8.2, 4.1 Hz, 1H), 7.16 (s, 1H), 4.55 – 3.68 (m, 2H), 3.30 – 2.98 (m, 2H), 2.35 (s, 3H), 2.32 (s, 3H). ^{13}C NMR (101 MHz, $CDCl_3$) δ 165.39, 150.26, 143.99, 140.62, 136.14, 135.86, 134.50, 134.32, 134.16, 129.57, 129.46, 128.77, 127.54, 126.85, 126.27, 121.29, 49.73, 25.08, 20.92, 18.98. IR

(film): 3442, 3039, 2921, 2856, 1650, 1600, 1471, 1427, 1336, 1228, 1147, 1114, 1031, 904, 871, 823, 788, 613, 566 cm^{-1}. HRMS calcd. for $C_{20}H_{18}N_2O$ $[M+H]^+$: 303.1492; found: 303.1494.

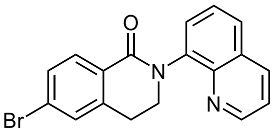

6-Bromo-2-(quinolin-8-yl)-3,4-dihydroisoquinolin-1(2H)-one (3da): white solid was obtained in 65% isolated yield following Method A. ^{1}H NMR (400 MHz, $CDCl_3$) δ 8.88 (dd, *J* = 4.1, 1.6 Hz, 1H), 8.17 (d, *J* = 8.3 Hz, 1H), 8.03 (d, *J* = 8.3 Hz, 1H), 7.81 (d, *J* = 8.2 Hz, 1H), 7.75 (dd, *J* = 7.3, 1.3 Hz, 1H), 7.58 (t, *J* = 7.8 Hz, 1H), 7.50 (dd, *J* = 8.3, 1.6 Hz, 1H), 7.44 (s, 1H), 7.40 (dd, *J* = 8.2, 4.2 Hz, 1H), 4.59 – 3.60 (m, 2H), 3.43 – 3.05 (m, 2H). ^{13}C NMR (101 MHz, $CDCl_3$) δ 164.07, 150.32, 143.75, 140.83, 140.04, 136.14, 130.34, 130.07, 129.83, 129.40, 128.68, 128.64, 127.75, 126.33, 126.20, 121.36, 49.87, 28.33. IR (film): 3448, 3062, 3033, 2954, 2923, 2856, 1650, 1587, 1471, 1313, 1270, 1234, 1135, 1068, 1022, 916, 877, 831, 701, 615, 520 cm^{-1}. HRMS calcd. for $C_{18}H_{13}BrN_2O$ $[M+H]^+$: 375.0103; found: 375.0109.

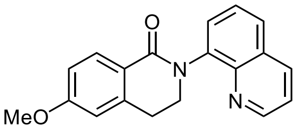

6-Methoxy-2-(quinolin-8-yl)-3,4-dihydroisoquinolin-1(2H)-one (3ea): white solid was obtained in 77% isolated yield following Method A. ^{1}H NMR (400 MHz, $CDCl_3$) δ 8.90 (dd, *J* = 4.2, 1.7 Hz, 1H), 8.19 (dd, *J* = 8.3, 1.7 Hz, 1H), 8.13 (d, *J* = 8.6 Hz, 1H), 7.81 (dd, *J* = 8.2, 1.3 Hz, 1H), 7.76 (dd, *J* = 7.3, 1.4 Hz, 1H), 7.61 – 7.57 (m, 1H), 7.40 (dd, *J* = 8.3, 4.2 Hz, 1H), 6.89 (dd, *J* = 8.6, 2.6 Hz, 1H), 6.77 (d, *J* = 2.5 Hz, 1H), 4.52 – 3.71 (m, 5H), 3.34 – 3.14 (m, 2H). ^{13}C NMR (101 MHz, $CDCl_3$) δ 164.93, 162.35, 150.35, 144.17, 141.12, 140.67, 136.21, 130.87, 129.54, 128.95, 127.56, 126.34, 122.71, 121.35, 112.35, 111.96, 55.37, 50.16, 29.10. IR (film): 3519, 2921, 2848, 1637, 1604, 1498, 1473, 1270, 1232, 1126, 1027, 917, 829, 792 cm^{-1}. HRMS calcd. for $C_{19}H_{16}N_2O_2$ $[M+Na]^+$: 327.1104; found: 327.1107.

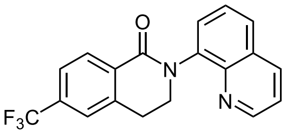

2-(Quinolin-8-yl)-6-(trifluoromethyl)-3,4-dihydroisoquinolin-1(2H)-one (3fa): white solid was obtained in 90% isolated yield following Method A. ^{1}H NMR (400 MHz, $CDCl_3$) δ 8.89 (dd, *J* =

4.2, 1.7 Hz, 1H), 8.30 (d, *J* = 8.1 Hz, 1H), 8.21 (dd, *J* = 8.3, 1.7 Hz, 1H), 7.85 (dd, *J* = 8.2, 1.3 Hz, 1H), 7.77 (dd, *J* = 7.3, 1.4 Hz, 1H), 7.66 – 7.59 (m, 2H), 7.57 (s, 1H), 7.43 (dd, *J* = 8.3, 4.2 Hz, 1H), 4.56 – 3.70 (m, 2H), 3.54 – 3.18 (m, 2H). ^{13}C NMR (101 MHz, $CDCl_3$) δ 163.71, 150.54, 143.83, 140.01, 139.67, 136.31, 133.29 (q, *J* = 32.4 Hz), 132.88, 129.58, 129.34, 128.79, 128.03, 126.36, 124.06 (q, *J* = 3.7 Hz), 123.80 (q, *J* = 3.8 Hz), 123.771 (q, *J* = 273.7 Hz), 121.56, 49.94, 28.63. ^{19}F NMR (376 MHz, $CDCl_3$) δ -62.81. IR (film): 3417, 3064, 3041, 2958, 2923, 2852, 1658, 1577, 1500, 1475, 1436, 1324, 1263, 1172, 1118, 1068, 1024, 925, 890, 829, 794, 703, 673 cm^{-1}. HRMS calcd. for $C_{19}H_{13}F_3N_2O$ $[M+H]^+$: 343.1053; found: 343.1059.

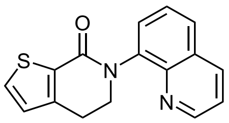

6-(Quinolin-8-yl)-5,6-dihydrothieno[2,3-c]pyridin-7(4H)-one (3ga): white solid was obtained in 77% isolated yield following Method A. ^{1}H NMR (400 MHz, $CDCl_3$) δ 8.90 (dd, *J* = 4.2, 1.7 Hz, 1H), 8.18 (dd, *J* = 8.3, 1.6 Hz, 1H), 7.81 (dd, *J* = 8.2, 1.2 Hz, 1H), 7.77 (dd, *J* = 7.3, 1.3 Hz, 1H), 7.58 (t, *J* = 7.8 Hz, 1H), 7.52 (d, *J* = 5.0 Hz, 1H), 7.41 (dd, *J* = 8.3, 4.2 Hz, 1H), 7.00 (d, *J* = 5.0 Hz, 1H), 4.70 – 3.76 (m, 2H), 3.30 – 3.08 (m, 2H). ^{13}C NMR (101 MHz, CDCl3) δ 161.71, 150.29, 144.68, 144.15, 139.79, 136.20, 132.40, 131.17, 129.47, 129.02, 127.67, 126.57, 126.25, 121.33, 51.51, 25.25. IR (film): 3426, 3104, 2950, 2923, 2856, 1637, 1494, 1454, 1432, 1334, 1243, 1205, 1151, 1118, 1027, 977, 879, 827, 786, 757, 725, 696, 620, 595 cm^{-1}. HRMS calcd. for $C_{16}H_{12}N_2OS$ $[M+Na]^+$: 303.0563; found: 303.0567.

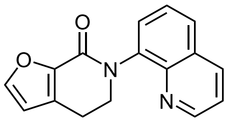

6-(Quinolin-8-yl)-5,6-dihydrofuro[2,3-c]pyridin-7(4H)-one (3ha): white solid was obtained in 75% isolated yield following Method A. ^{1}H NMR (400 MHz, $CDCl_3$) δ 8.91 (dd, *J* = 4.1, 1.6 Hz, 1H), 8.18 (dd, *J* = 8.3, 1.6 Hz, 1H), 7.85 – 7.73 (m, 2H), 7.62 – 7.53 (m, 2H), 7.41 (dd, *J* = 8.3, 4.2 Hz, 1H), 6.46 (d, *J* = 1.7 Hz, 1H), 4.63 – 3.64 (m, 2H), 3.20 – 2.87 (m, 2H). ^{13}C NMR (101 MHz, $CDCl_3$) δ 158.61, 150.30, 146.05, 144.16, 143.72, 139.29, 136.29, 129.85, 129.53, 129.15, 127.78, 126.31, 121.39, 110.29, 51.79, 22.19. IR (film): 3430, 3118, 2962, 2923, 2858, 1672, 1596, 1467, 1442, 1403, 1321, 1193, 1129, 1089, 1010, 970, 898, 829, 784, 597 cm^{-1}. HRMS calcd. for $C_{16}H_{12}N_2O_2$ $[M+H]^+$: 265.0972; found: 265.0966.

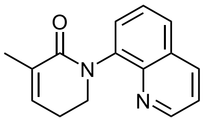

3-Methyl-1-(quinolin-8-yl)-5,6-dihydropyridin-2(1H)-one (3ia): pale yellow solid was obtained in 55% isolated yield following Method A. ^{1}H NMR (400 MHz, $CDCl_3$) δ 8.91 (dd, *J* = 4.1, 1.6 Hz, 1H), 8.16 (dd, *J* = 8.3, 1.5 Hz, 1H), 7.77 (dd, *J* = 8.2, 1.0 Hz, 1H), 7.69 (dd, *J* = 7.3, 1.2 Hz, 1H), 7.55 (t, *J* = 7.8 Hz, 1H), 7.39 (dd, *J* = 8.3, 4.2 Hz, 1H), 6.55 – 6.47 (m, 1H), 4.21 – 3.67 (m, 2H), 2.66 – 2.51 (m, 2H), 2.02 – 1.95 (m, 3H). ^{13}C NMR (101 MHz, $CDCl_3$) δ 166.12, 150.24, 144.04, 140.55, 136.20, 135.00, 131.85, 129.47, 128.84, 127.40, 126.26, 121.25, 49.86, 24.55, 17.27. IR (film): 3361, 2921, 2850, 1670, 1633, 1496, 1473, 1427, 1357, 1321, 1297, 1197, 1133, 1049, 979, 941, 831, 794, 750, 636, 572 cm^{-1}. HRMS calcd. for $C_{15}H_{14}N_2O$ [M+Na]$^+$: 261.0998; found: 261.1004.

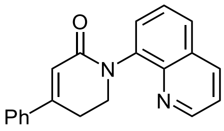

4-Phenyl-1-(quinolin-8-yl)-5,6-dihydropyridin-2(1H)-one (3ja): white solid was obtained in 52% isolated yield following Method A. ^{1}H NMR (400 MHz, $CDCl_3$) δ 8.94 (dd, *J* = 4.2, 1.7 Hz, 1H), 8.18 (dd, *J* = 8.3, 1.7 Hz, 1H), 7.81 (dd, *J* = 8.2, 1.3 Hz, 1H), 7.77 (dd, *J* = 7.3, 1.4 Hz, 1H), 7.64 – 7.55 (m, 3H), 7.47 – 7.37 (m, 4H), 6.58 – 6.52 (m, 1H), 4.51 – 3.72 (m, 2H), 3.19 – 2.95 (m, 2H). ^{13}C NMR (101 MHz, $CDCl_3$) δ 165.63, 150.54, 150.34, 144.06, 139.92, 137.76, 136.32, 129.60, 129.44, 129.01, 128.73, 127.67, 126.38, 125.86, 121.40, 120.37, 49.52, 27.24. IR (film): 3432, 3062, 2952, 2921, 1652, 1602, 1473, 1434, 1344, 1240, 1157, 1024, 867, 833, 788, 761, 715, 682, 588 cm^{-1}. HRMS calcd. for $C_{20}H_{16}N_2O$ [M+H]$^+$: 301.1335; found: 301.1325.

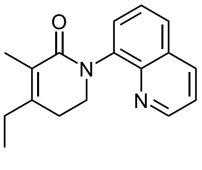

4-Ethyl-3-methyl-1-(quinolin-8-yl)-5,6-dihydropyridin-2(1H)-one (3ka): pale yellow solid was obtained in 80% isolated yield following Method A. ^{1}H NMR (400 MHz, $CDCl_3$) δ 8.91 (dd, *J* = 4.2, 1.7 Hz, 1H), 8.15 (dd, *J* = 8.3, 1.7 Hz, 1H), 7.76 (dd, *J* = 8.2, 1.4 Hz, 1H), 7.68 (dd, *J* = 7.3, 1.4 Hz, 1H), 7.57 – 7.51 (m, 1H), 7.38 (dd, *J* = 8.3, 4.2 Hz, 1H), 4.27 – 3.44 (m, 2H), 2.68 – 2.50 (m, 2H), 2.32 (q, *J* = 7.6 Hz, 2H), 1.98 – 1.93 (m, 3H), 1.13 (t, *J* = 7.6 Hz, 3H). ^{13}C NMR (101 MHz, $CDCl_3$) δ 166.59, 150.13, 149.07, 144.14, 140.81, 136.16, 129.45, 128.90, 127.18, 126.23, 124.70,

121.15, 48.84, 28.49, 27.17, 11.85, 11.70. IR (film): 3425, 2966, 2873, 2933, 1658, 1631, 1496, 1471, 1430, 1378, 1321, 1236, 1159, 1135, 1103, 985, 950, 894, 829, 792, 755, 640, 551 cm^{-1}. HRMS calcd. for $C_{17}H_{18}N_2O$ $[M+H]^+$: 267.1492; found: 267.1492.

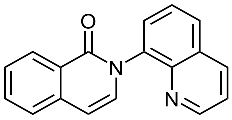

2-(Quinolin-8-yl)isoquinolin-1(2H)-one (3ab): white solid was obtained in 85% isolated yield following Method B. ^1H NMR (400 MHz, $CDCl_3$) δ 8.86 (dd, *J* = 4.2, 1.7 Hz, 1H), 8.49 (d, *J* = 8.0 Hz, 1H), 8.17 (dd, *J* = 8.3, 1.6 Hz, 1H), 7.89 (dd, *J* = 8.3, 1.2 Hz, 1H), 7.78 (dd, *J* = 7.3, 1.3 Hz, 1H), 7.67 – 7.59 (m, 2H), 7.56 (d, *J* = 7.7 Hz, 1H), 7.51 – 7.45 (m, 1H), 7.38 (dd, *J* = 8.3, 4.2 Hz, 1H), 7.17 (d, *J* = 7.4 Hz, 1H), 6.59 (d, *J* = 7.4 Hz, 1H). ^{13}C NMR (101 MHz, $CDCl_3$) δ 162.37, 151.00, 143.62, 138.36, 137.38, 136.07, 133.25, 132.29, 129.22, 128.92, 128.80, 128.10, 126.67, 126.48, 125.99, 125.90, 121.62, 105.32. IR (film): 3440, 3033, 2925, 1949, 1839, 1652, 1496, 1409, 1382, 1336, 1276, 1133, 908, 829, 788, 692, 580 cm^{-1}. HRMS calcd. for $C_{18}H_{12}N_2O$ $[M+H]^+$: 273.1022; found: 273.1025.

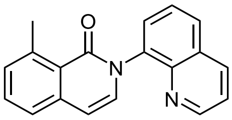

8-Methyl-2-(quinolin-8-yl)isoquinolin-1(2H)-one (3bb): pale yellow solid was obtained in 73% isolated yield following Method B. ^1H NMR (400 MHz, $CDCl_3$) δ 8.91 (dd, *J* = 4.2, 1.6 Hz, 1H), 8.23 (dd, *J* = 8.3, 1.6 Hz, 1H), 7.93 (dd, *J* = 8.2, 1.1 Hz, 1H), 7.80 (dd, *J* = 7.3, 1.2 Hz, 1H), 7.69 – 7.63 (m, 1H), 7.50 (t, *J* = 7.6 Hz, 1H), 7.45 – 7.38 (m, 2H), 7.27 – 7.22 (m, 1H), 7.14 (d, *J* = 7.3 Hz, 1H), 6.55 (d, *J* = 7.3 Hz, 1H), 2.90 (s, 3H). ^{13}C NMR (101 MHz, $CDCl_3$) δ 163.40, 151.19, 143.95, 142.47, 139.37, 138.92, 136.18, 132.99, 131.72, 130.01, 129.44, 129.16, 128.84, 126.20, 125.05, 124.47, 121.76, 105.90, 24.04. IR (film): 3436, 2962, 2921, 1658, 1627, 1496, 1427, 1384, 1315, 1284, 1124, 880, 831, 784, 686, 576 cm^{-1}. HRMS calcd. for $C_{19}H_{14}N_2O$ $[M+H]^+$: 287.1179; found: 287.1180.

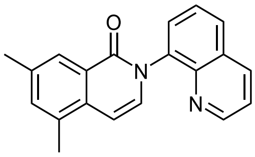

5,7-Dimethyl-2-(quinolin-8-yl)isoquinolin-1(2H)-one (3cb): pale yellow solid was obtained in 85% isolated yield following Method B. ^1H NMR (400 MHz, $CDCl_3$) δ 8.90 (dd, *J* = 4.2, 1.7 Hz, 1H),

8.22 (dd, *J* = 8.3, 1.6 Hz, 1H), 8.17 (s, 1H), 7.93 (dd, *J* = 8.2, 1.2 Hz, 1H), 7.81 (dd, *J* = 7.3, 1.3 Hz, 1H), 7.69 – 7.63 (m, 1H), 7.43 (dd, *J* = 8.3, 4.2 Hz, 1H), 7.35 (s, 1H), 7.18 (d, *J* = 7.6 Hz, 1H), 6.72 (d, *J* = 7.6 Hz, 1H), 2.55 (s, 3H), 2.45 (s, 3H). ^{13}C NMR (101 MHz, $CDCl_3$) δ 162.72, 151.14, 143.81, 138.67, 136.42, 136.16, 134.81, 134.00, 133.13, 131.96, 129.36, 128.93, 128.92, 126.76, 126.14, 125.80, 121.69, 102.15, 21.39, 18.93. IR (film): 3428, 2954, 2921, 2854, 1652, 1608, 1496, 1382, 1321, 1295, 1259, 1191, 1033, 904, 875, 831, 792, 754, 696, 572 cm^{-1}. HRMS calcd. for $C_{20}H_{16}N_2O$ [M+H]$^+$: 301.1335; found: 301.1345.

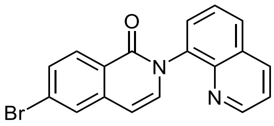

6-Bromo-2-(quinolin-8-yl)isoquinolin-1(2H)-one (3db): white solid was obtained in 68% isolated yield following Method B. ^{1}H NMR (400 MHz, $CDCl_3$) δ 8.90 (dd, *J* = 4.2, 1.6 Hz, 1H), 8.33 (d, *J* = 8.6 Hz, 1H), 8.24 (dd, *J* = 8.3, 1.5 Hz, 1H), 7.96 (dd, *J* = 8.2, 0.9 Hz, 1H), 7.81 (dd, *J* = 7.3, 1.0 Hz, 1H), 7.76 (d, *J* = 1.7 Hz, 1H), 7.67 (t, *J* = 7.8 Hz, 1H), 7.60 (dd, *J* = 8.6, 1.8 Hz, 1H), 7.45 (dd, *J* = 8.3, 4.2 Hz, 1H), 7.22 (d, *J* = 7.4 Hz, 1H), 6.53 (d, *J* = 7.4 Hz, 1H). ^{13}C NMR (101 MHz, $CDCl_3$) δ 162.04, 151.21, 143.61, 138.96, 138.13, 136.23, 134.74, 130.17, 130.04, 129.36, 129.20, 128.85, 128.39, 127.54, 126.13, 125.29, 121.84, 104.30. IR (film): 3428, 3050, 2925, 2850, 2362, 1656, 1627, 1591, 1498, 1467, 1375, 1317, 1286, 1135,1066, 885, 829, 790, 769, 680, 584 cm^{-1}. HRMS calcd. for $C_{18}H_{11}BrN_2O$ [M+Na]$^+$: 375.0103; found: 375.0109.

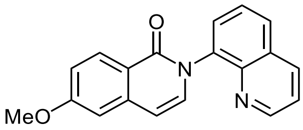

6-Methoxy-2-(quinolin-8-yl)isoquinolin-1(2H)-one (3eb): white solid was obtained in 80% isolated yield following Method B. ^{1}H NMR (400 MHz, $CDCl_3$) δ 8.90 (dd, *J* = 4.2, 1.7 Hz, 1H), 8.40 (d, *J* = 8.9 Hz, 1H), 8.23 (dd, *J* = 8.3, 1.7 Hz, 1H), 7.93 (dd, *J* = 8.2, 1.3 Hz, 1H), 7.81 (dd, *J* = 7.3, 1.4 Hz, 1H), 7.65 (dd, *J* = 8.1, 7.4 Hz, 1H), 7.43 (dd, *J* = 8.3, 4.2 Hz, 1H), 7.18 (d, *J* = 7.4 Hz, 1H), 7.08 (dd, *J* = 8.9, 2.5 Hz, 1H), 6.95 (d, *J* = 2.5 Hz, 1H), 6.54 (d, *J* = 7.4 Hz, 1H), 3.92 (s, 3H). ^{13}C NMR (101 MHz, $CDCl_3$) δ 162.86, 162.21, 151.12, 143.85, 139.60, 138.50, 136.19, 133.99, 130.36, 129.35, 129.02, 128.93, 126.12, 121.70, 120.41, 116.09, 106.98, 105.26, 55.45. IR (film): 3434, 3058, 2994, 2958, 2923, 2852, 1654, 1627, 1602, 1473, 1388, 1303, 1242, 1164, 1130, 1029, 902, 846, 794, 755, 684, 574 cm^{-1}. HRMS calcd. for $C_{19}H_{14}N_2O_2$ [M+H]$^+$: 303.1128; found: 303.1128.

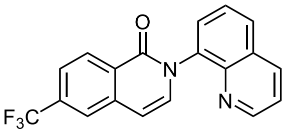

2-(Quinolin-8-yl)-6-(trifluoromethyl)isoquinolin-1(2H)-one (3fb): white solid was obtained in 89% isolated yield following Method B. ^{1}H NMR (400 MHz, $CDCl_3$) δ 8.89 (dd, *J* = 4.2, 1.7 Hz, 1H), 8.60 (dd, *J* = 8.4, 0.4 Hz, 1H), 8.25 (dd, *J* = 8.3, 1.7 Hz, 1H), 7.97 (dd, *J* = 8.3, 1.3 Hz, 1H), 7.88 (s, 1H), 7.82 (dd, *J* = 7.3, 1.4 Hz, 1H), 7.75 – 7.62 (m, 2H), 7.46 (dd, *J* = 8.3, 4.2 Hz, 1H), 7.29 (d, *J* = 7.4 Hz, 1H), 6.67 (d, *J* = 7.4 Hz, 1H). ^{13}C NMR (101 MHz, $CDCl_3$) δ 161.72, 151.26, 143.56, 138.03, 137.50, 136.27, 134.93, 134.00 (q, *J* = 32.6 Hz), 129.42, 129.41, 129.35, 128.80, 128.76， 126.16, 123.74 (q, *J* = 273.9 Hz), 123.33 (q, *J* = 4.1 Hz), 122.79 (q, *J* = 3.4 Hz), 121.92, 105.00. ^{19}F NMR (376 MHz, $CDCl_3$) δ -62.91. IR (film): 3421, 3048, 2956, 2923, 2852, 1656, 1620, 1631, 1560, 1500, 1459, 1388, 1351, 1321, 1290, 1164, 1105, 1064, 919, 894, 829, 792, 761, 696 cm^{-1}. HRMS calcd. for $C_{19}H_{11}F_3N_2O$ $[M+H]^+$: 341.0896; found: 341.0893.

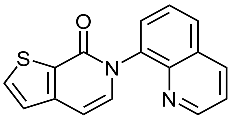

6-(Quinolin-8-yl)thieno[2,3-c]pyridin-7(6H)-one (3gb): white solid was obtained in 83% isolated yield following Method B. ^{1}H NMR (400 MHz, $CDCl_3$) δ 8.91 (dd, *J* = 4.2, 1.7 Hz, 1H), 8.24 (dd, *J* = 8.3, 1.6 Hz, 1H), 7.95 (dd, *J* = 8.3, 1.2 Hz, 1H), 7.83 (dd, *J* = 7.3, 1.3 Hz, 1H), 7.74 (d, *J* = 5.2 Hz, 1H), 7.70 – 7.63 (m, 1H), 7.45 (dd, *J* = 8.3, 4.2 Hz, 1H), 7.32 – 7.27 (m, 2H), 6.77 (d, *J* = 7.1 Hz, 1H). ^{13}C NMR (101 MHz, $CDCl_3$) δ 158.72, 151.23, 145.73, 143.78, 137.86, 136.21, 134.78, 133.74, 130.64, 129.35, 129.21, 129.09, 126.06, 124.40, 121.78, 102.41. IR (film): 3432, 3058, 2996, 2958, 2925, 2854, 1654, 1625, 1600, 1473, 1390, 1355, 1301, 1286, 1241, 1193, 1166, 1130, 1091, 1029, 902, 846, 796, 759, 686, 574 cm^{-1}. HRMS calcd. for $C_{16}H_{10}N_2OS$ $[M+H]^+$: 279.0587; found: 279.0592.

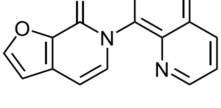

6-(Quinolin-8-yl)furo[2,3-c]pyridin-7(6H)-one (3hb): white solid was obtained in 65% isolated yield following Method B. ^{1}H NMR (400 MHz, $CDCl_3$) δ 8.91 (dd, *J* = 4.1, 1.5 Hz, 1H), 8.24 (dd, *J* = 8.3, 1.4 Hz, 1H), 7.95 (d, *J* = 8.2 Hz, 1H), 7.87 – 7.73 (m, 2H), 7.66 (t, *J* = 7.8 Hz, 1H), 7.45 (dd, *J* = 8.3, 4.2 Hz, 1H), 7.21 (d, *J* = 7.0 Hz, 1H), 6.74 (d, *J* = 1.9 Hz, 1H), 6.59 (d, *J* = 7.0 Hz, 1H).

^{13}C NMR (101 MHz, $CDCl_3$) δ 153.76, 151.23, 148.36, 143.79, 143.73, 137.58, 136.22, 134.27, 133.28, 129.39, 129.32, 129.10, 126.07, 121.81, 107.50, 99.66. IR (film): 3425, 3120, 3060, 2956, 2921, 2850, 1672, 1585, 1552, 1492, 1386, 1319, 1280, 1193, 1128, 1083, 1037, 1010, 983, 829, 796, 761, 669, 599 cm^{-1}. HRMS calcd. for $C_{16}H_{10}N_2O_2$ $[M+H]^+$: 263.0815; found: 263.0822.

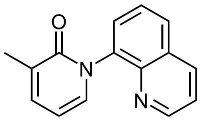

3-Methyl-1-(quinolin-8-yl)pyridin-2(1H)-one (3ib): pale yellow solid was obtained in 49% isolated yield following Method B. ^{1}H NMR (400 MHz, $CDCl_3$) δ 8.90 (dd, *J* = 4.2, 1.7 Hz, 1H), 8.20 (dd, *J* = 8.3, 1.7 Hz, 1H), 7.90 (dd, *J* = 8.2, 1.4 Hz, 1H), 7.74 (dd, *J* = 7.3, 1.4 Hz, 1H), 7.65 – 7.58 (m, 1H), 7.41 (dd, *J* = 8.3, 4.2 Hz, 1H), 7.37 – 7.32 (m, 1H), 7.26 – 7.22 (m, 1H), 6.22 (t, *J* = 6.8 Hz, 1H), 2.22 (s, 3H). ^{13}C NMR (101 MHz, $CDCl_3$) δ 163.07, 151.08, 143.35, 138.30, 137.27, 136.45, 136.03, 130.31, 129.14, 128.96, 128.44, 125.87, 121.60, 104.82, 17.21. IR (film): 3428, 2942, 2923, 2850, 1940, 1830, 1652, 1592, 1550, 1494, 1373, 1317, 1268, 1135, 1068, 977, 890, 862, 825, 790, 759, 669, 598 cm^{-1}. HRMS calcd. for $C_{15}H_{12}N_2O$ $[M+H]^+$: 237.1022; found: 237.1024.

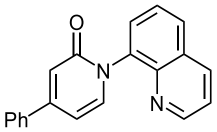

4-Phenyl-1-(quinolin-8-yl)pyridin-2(1H)-one (3jb): white solid was obtained in 79% isolated yield following Method B. ^{1}H NMR (400 MHz, $CDCl_3$) δ 8.94 (dd, *J* = 4.1, 1.5 Hz, 1H), 8.22 (dd, *J* = 8.3, 1.5 Hz, 1H), 7.93 (d, *J* = 7.6 Hz, 1H), 7.81 (d, *J* = 7.3 Hz, 1H), 7.70 – 7.64 (m, 3H), 7.48 – 7.40 (m, 5H), 6.97 (d, *J* = 1.8 Hz, 1H), 6.58 (dd, *J* = 7.1, 1.9 Hz, 1H). ^{13}C NMR (101 MHz, $CDCl_3$) δ 162.92, 152.18, 151.18, 143.32, 139.03, 137.68, 137.48, 136.19, 129.34, 129.23, 128.85, 128.63, 126.74, 126.03, 121.78, 117.75, 104.88. IR (film): 3363, 3060, 2956, 2921, 2854, 1660, 1594, 1494, 1461, 1384, 1332, 1278, 1240, 968, 917, 856, 829, 788, 752, 690, 607, 563 cm^{-1}. HRMS calcd. for $C_{20}H_{14}N_2O$ $[M+H]^+$: 299.1179; found: 299.1181.

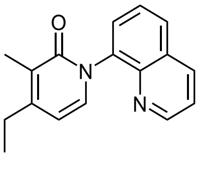

4-Ethyl-3-methyl-1-(quinolin-8-yl)pyridin-2(1H)-one (3kb): pale yellow solid was obtained in 76% isolated yield following Method B. ^{1}H NMR (400 MHz, $CDCl_3$) δ 8.93 (dd, *J* = 4.2, 1.7 Hz, 1H), 8.22 (dd, *J* = 8.3, 1.6 Hz, 1H), 7.92 (dd, *J* = 8.2, 1.3 Hz, 1H), 7.74 (dd, *J* = 7.3, 1.3 Hz, 1H), 7.66 – 7.60 (m, 1H), 7.43 (dd, *J* = 8.3, 4.2 Hz, 1H), 7.20 (d, *J* = 7.0 Hz, 1H), 6.20 (d, *J* = 7.0 Hz, 1H), 2.61 (q, *J* = 13.7, 7.2 Hz, 2H), 2.19 (s, 3H), 1.26 (t, *J* = 7.6 Hz, 3H). ^{13}C NMR (101 MHz, $CDCl_3$) δ 163.15, 151.96, 151.21, 143.61, 138.67, 136.14, 134.89, 129.29, 128.91, 128.70, 126.06, 126.02, 121.64, 106.90, 26.64, 13.18, 12.10. IR (film): 3434, 3035, 2962, 2925, 2875, 1945, 1645, 1591, 1535, 1494, 1467, 1369, 1324, 1274, 1189, 1132, 1097, 1065, 975, 890, 831, 792, 769, 669, 580 cm^{-1}. HRMS calcd. for $C_{17}H_{16}N_2O$ $[M+H]^+$: 265.1335; found: 265.1329.

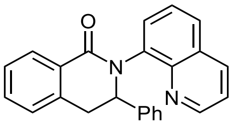

3-Phenyl-2-(quinolin-8-yl)-3,4-dihydroisoquinolin-1(2H)-one (3ac):[3] white solid was obtained in 84 % isolated yield following Method A (2 eq. of styrene was used instead of ethylene under nitrogen atmosphere). ^{1}H NMR (400 MHz, DMSO-d_6) δ 8.98 (d, *J* = 2.8 Hz, 1H), 8.41 (d, *J* = 8.0 Hz, 1H), 8.03 – 7.97 (m, 1H), 7.91 (d, *J* = 8.5 Hz, 1H), 7.63 – 7.45 (m, 4H), 7.41 (t, *J* = 7.2 Hz, 1H), 7.31 – 7.21 (m, 3H), 7.21 – 7.04 (m, 3H), 5.55 – 5.36 (m, 1H), 4.13 – 3.79 (m, 1H), 3.36 – 3.20 (m, 1H). ^{13}C NMR (101 MHz, DMSO-d_6) δ 164.05, 150.78, 143.74, 140.59, 139.06, 136.84, 136.64, 132.36, 129.97, 129.50, 129.08, 128.24, 127.95, 127.85, 127.48, 127.29, 127.05, 126.90, 125.99, 121.82, 62.50, 35.97.

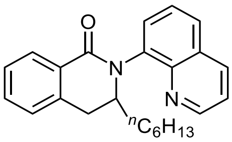

3-Hexyl-2-(quinolin-8-yl)-3,4-dihydroisoquinolin-1(2H)-one (3ad): pale yellow oil was obtained in 63 % isolated yield following Method A (2 eq. of 1-octene was used instead of ethylene under nitrogen atmosphere), the product was obtained as a mixture of diastereomers (4:1). ^{1}H NMR (400 MHz, DMSO-d_6) δ 9.00 – 8.90 (m, 0.8H), 8.88 (dd, *J* = 4.2, 1.7 Hz, 0.2H), 8.53 – 8.42 (m, 1H), 8.06 – 8.00 (m, 1H), 7.98 – 7.88 (m, 1H), 7.83 – 7.73 (m, 1H), 7.72 – 7.65 (m, 1H), 7.63 – 7.52 (m, 2H), 7.45 – 7.36 (m, 2H), 4.66 – 4.28 (m, 0.2H), 4.15 – 4.01 (m, 0.8H), 3.85 – 3.47 (m, 1H), 3.13 – 2.96 (m, 1H), 1.88 – 1.73 (m, 0.3H), 1.62 – 1.39 (m, 1.7H), 1.36 – 1.13 (m, 3H), 1.13 – 0.93 (m, 5H), 0.86 – 0.79 (m, 0.8H), 0.70 (t, *J* = 7.0 Hz, 2.3H). ^{13}C NMR (101 MHz, DMSO-d_6) δ 163.59,

151.15, 144.12, 143.93, 141.26, 137.96, 137.04, 136.94, 132.50, 132.34, 129.85, 129.49, 128.93, 128.46, 128.30, 128.19, 127.86, 127.22, 127.17, 127.01, 126.56, 122.23, 122.17, 58.84, 33.78, 32.58, 31.61, 31.33, 29.16, 28.81, 27.14, 25.77, 22.49, 22.25, 14.35, 14.23. IR (film): 3486, 3411, 3033, 2925, 2858, 2242, 1735, 1598, 1650, 1496, 1465, 1417, 1313, 1128, 914, 1029, 827, 792, 734, 613, 574 cm^{-1}. HRMS calcd. for $C_{24}H_{26}N_2O$ $[M+H]^+$: 359.2118; found: 359.2108.

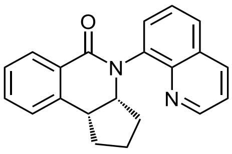

4-(Quinolin-8-yl)-1,2,3,3a,4,9b-hexahydro-5H-cyclopenta[c]isoquinolin-5-one (3ae):[3] white solid was obtained in 58 % isolated yield following Method A (2 eq. of cyclopentene was used instead of ethylene under nitrogen atmosphere). ^{1}H NMR (400 MHz, $CDCl_3$) δ 8.92 – 8.79 (m, 1H), 8.27 – 8.12 (m, 2H), 7.85 (d, *J* = 8.1 Hz, 1H), 7.68 (d, *J* = 7.2 Hz, 1H), 7.60 (t, *J* = 7.7 Hz, 1H), 7.50 (td, *J* = 7.5, 1.4 Hz, 1H), 7.42 – 7.29 (m, 3H), 5.08 – 4.89 (m, 0.8H), 4.72 – 4.53 (m, 0.2H), 3.58 – 3.39 (m, 1H), 2.47 – 1.98 (m, 2H), 1.96 – 1.50 (m, 4H). ^{13}C NMR (101 MHz, $CDCl_3$) δ 150.37, 144.47, 141.29, 138.06, 136.25, 132.04, 130.57, 129.69, 129.66, 129.08, 127.86, 127.51, 126.95, 126.67, 126.17, 121.36, 63.04, 42.43, 32.88, 32.28, 22.11.

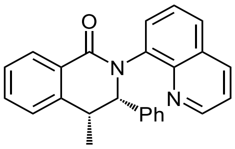

4-Methyl-3-phenyl-2-(quinolin-8-yl)-3,4-dihydroisoquinolin-1(2H)-one (3af): white solid was obtained in 50 % isolated yield following Method A (2 eq. of *E*-prop-1-en-1-ylbenzene was used instead of ethylene under nitrogen atmosphere). ^{1}H NMR (400 MHz, $CDCl_3$) δ 8.91 (dd, *J* = 4.1, 1.7 Hz, 1H), 8.27 (dd, *J* = 7.6, 1.5 Hz, 1H), 8.14 (dd, *J* = 8.3, 1.8 Hz, 1H), 7.80 – 7.69 (m, 1H), 7.50 – 7.30 (m, 5H), 7.23 – 7.08 (m, 6H), 5.06 (d, *J* = 2.3 Hz, 1H), 3.29 (dd, *J* = 7.0, 2.3 Hz, 1H), 1.87 (d, *J* = 7.1 Hz, 3H). ^{13}C NMR (101 MHz, $CDCl_3$) δ 164.38, 150.06, 144.26, 142.05, 141.75, 139.69, 136.04, 132.34, 129.33, 128.77, 128.48, 128.34, 127.75, 127.36, 127.31, 126.96, 126.77, 126.02, 121.34, 69.58, 41.99, 23.72. IR (film): 3436, 2962, 2917, 1645, 1492, 1463, 1340, 1265, 1128, 885, 821, 790, 757, 698, 574 cm^{-1}. HRMS calcd. for $C_{17}H_{16}N_2O$ $[M+Na]^+$: 387.1468; found: 387.1469.

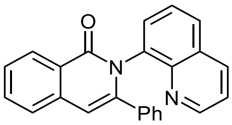

3-Phenyl-2-(quinolin-8-yl)isoquinolin-1(2H)-one (3ag):[7] white solid was obtained in 80 % isolated yield following Method B (2 eq. of phenylacetylene was used instead of ethylene under nitrogen atmosphere). ^{1}H NMR (400 MHz, $CDCl_3$) δ 8.90 (dd, *J* = 4.2, 1.7 Hz, 1H), 8.51 – 8.44 (m, 1H), 8.08 (dd, *J* = 8.3, 1.8 Hz, 1H), 7.75 – 7.66 (m, 2H), 7.60 (d, *J* = 7.7 Hz, 1H), 7.53 – 7.46 (m, 2H), 7.44 – 7.33 (m, 2H), 7.15 – 7.08 (m, 2H), 7.06 – 6.99 (m, 1H), 6.98 – 6.91 (m, 2H), 6.65 (d, *J* = 1 Hz, 1H). ^{13}C NMR (101 MHz, $CDCl_3$) δ 163.17, 150.91, 144.74, 144.43, 137.31, 137.20, 136.27, 135.98, 132.63, 130.61, 128.72, 128.63, 128.41, 127.90, 127.24, 126.65, 126.08, 125.75, 125.40, 121.47, 107.35.

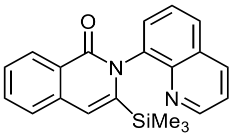

2-(Quinolin-8-yl)-3-(trimethylsilyl)isoquinolin-1(2H)-one (3ah): white solid was obtained in 74 % isolated yield following Method B (2 eq. of trimethylsilylacetylene was used instead of ethylene under nitrogen atmosphere). ^{1}H NMR (400 MHz, $CDCl_3$) δ 8.88 (dd, *J* = 4.2, 1.7 Hz, 1H), 8.46 – 8.39 (m, 1H), 8.22 (dd, *J* = 8.3, 1.8 Hz, 1H), 7.98 (dd, *J* = 8.0, 1.8 Hz, 1H), 7.75 – 7.64 (m, 3H), 7.59 (d, *J* = 7.7 Hz, 1H), 7.51 (ddd, *J* = 8.2, 7.1, 1.3 Hz, 1H), 7.42 (dd, *J* = 8.3, 4.2 Hz, 1H), 6.90 (s, 1H), -0.27 (s, 9H). ^{13}C NMR (101 MHz, $CDCl_3$) δ 163.58, 151.32, 146.70, 145.44, 139.24, 136.85, 136.09, 132.30, 130.42, 129.47, 129.17, 127.97, 127.28, 126.34, 126.13, 125.93, 121.81, 115.40, -0.25. IR (film): 3056, 2948, 2925, 2850, 1650, 1496, 1386, 1334, 1247, 1186, 1132, 1024, 948, 833, 792, 759, 690 cm^{-1}. HRMS calcd. for $C_{21}H_{20}N_2OSi$ $[M+H]^+$: 345.1418; found: 345.1412.

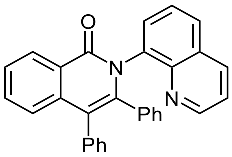

3,4-Diphenyl-2-(quinolin-8-yl)isoquinolin-1(2H)-one (3ai):[7] white solid was obtained in 60 % isolated yield following Method B (2 eq. of diphenylacetylene was used instead of ethylene under nitrogen atmosphere). ^{1}H NMR (400 MHz, $CDCl_3$) δ 8.93 (dd, *J* = 4.2, 1.6 Hz, 1H), 8.59 (d, *J* = 7.9 Hz, 1H), 8.05 (dd, *J* = 8.3, 1.6 Hz, 1H), 7.66 (dd, *J* = 8.2, 1.2 Hz, 1H), 7.63 – 7.58 (m, 1H), 7.55 – 7.47 (m, 2H), 7.40 – 7.34 (m, 2H), 7.31 (d, *J* = 8.1 Hz, 1H), 7.26 – 7.23 (m, 2H), 7.19 – 7.12 (m, 3H), 6.97 (d, *J* = 7.7 Hz, 1H), 6.83 (t, *J* = 7.5 Hz, 1H), 6.77 – 6.69 (m, 2H), 6.49 (t, *J* = 7.6 Hz, 1H). ^{13}C NMR (101 MHz, $CDCl_3$) δ 162.67, 150.76, 144.69, 141.80, 138.10, 137.67, 136.52, 135.95,

134.86, 132.41, 131.81, 131.63, 130.81, 130.74, 129.73, 128.69, 128.52, 128.38, 127.98, 127.74, 127.16, 126.71, 126.63, 126.60, 126.37, 125.71, 125.60, 125.54, 121.45, 118.47.

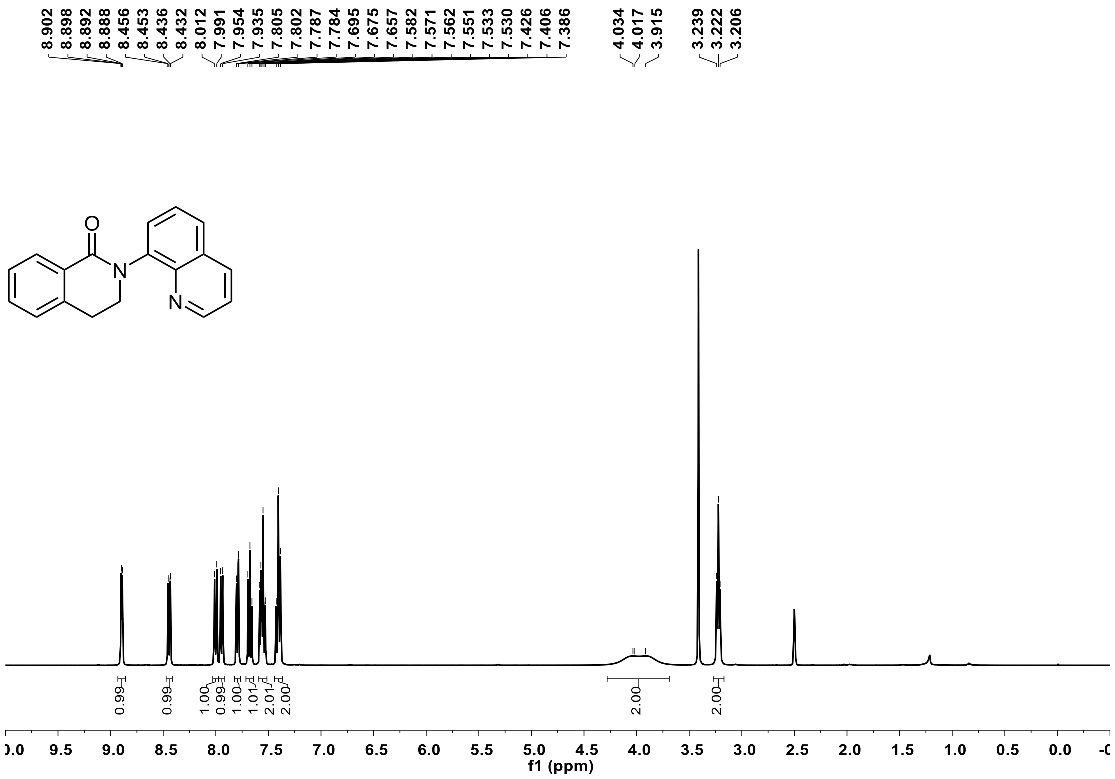

Supplementary Figure 4. ^{1}H NMR (400 MHz, DMSO-d_6) spectrum of 3aa

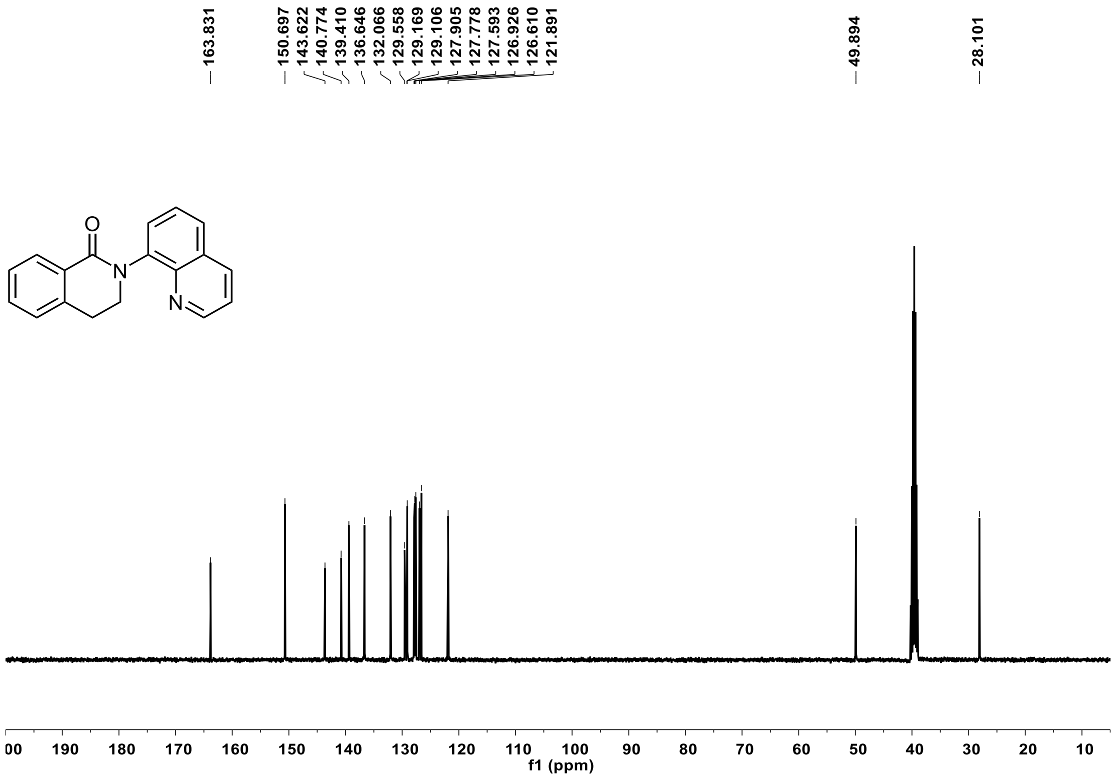

Supplementary Figure 5. ^{13}C NMR (101 MHz, DMSO-d_6) spectrum of 3aa

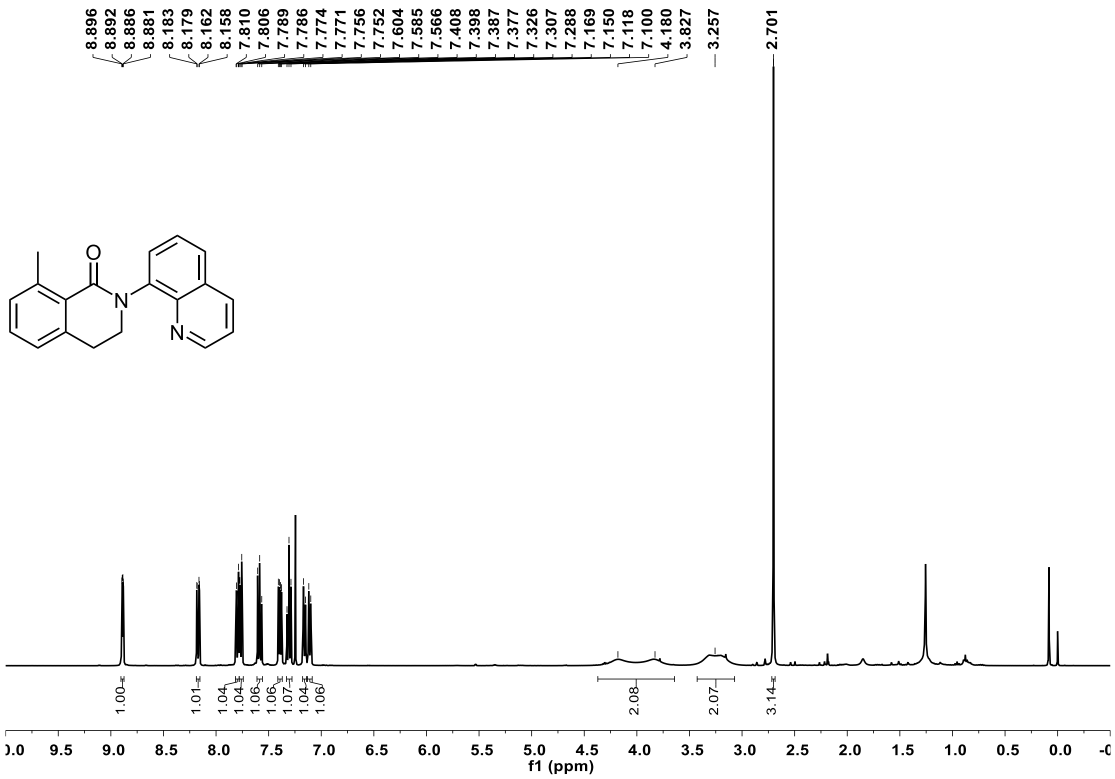

Supplementary Figure 6. ^{1}H NMR (400 MHz, $CDCl_3$) spectrum of 3ba

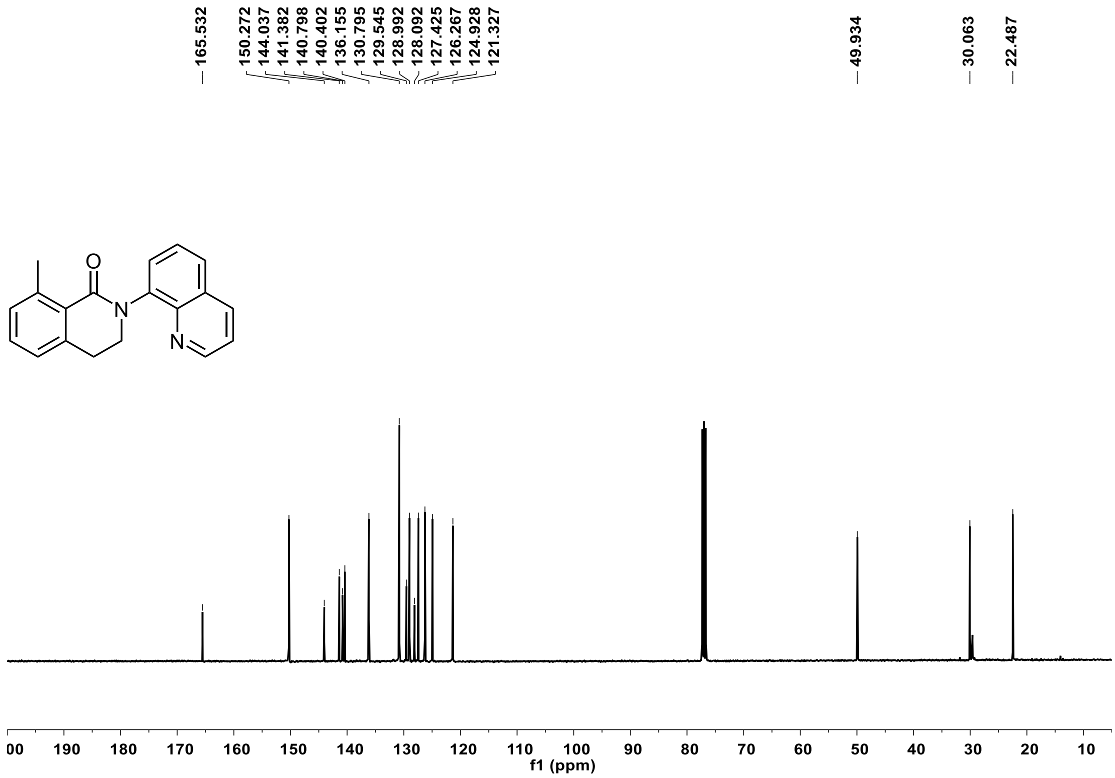

Supplementary Figure 7. ^{13}C NMR (101 MHz, $CDCl_3$) spectrum of 3ba

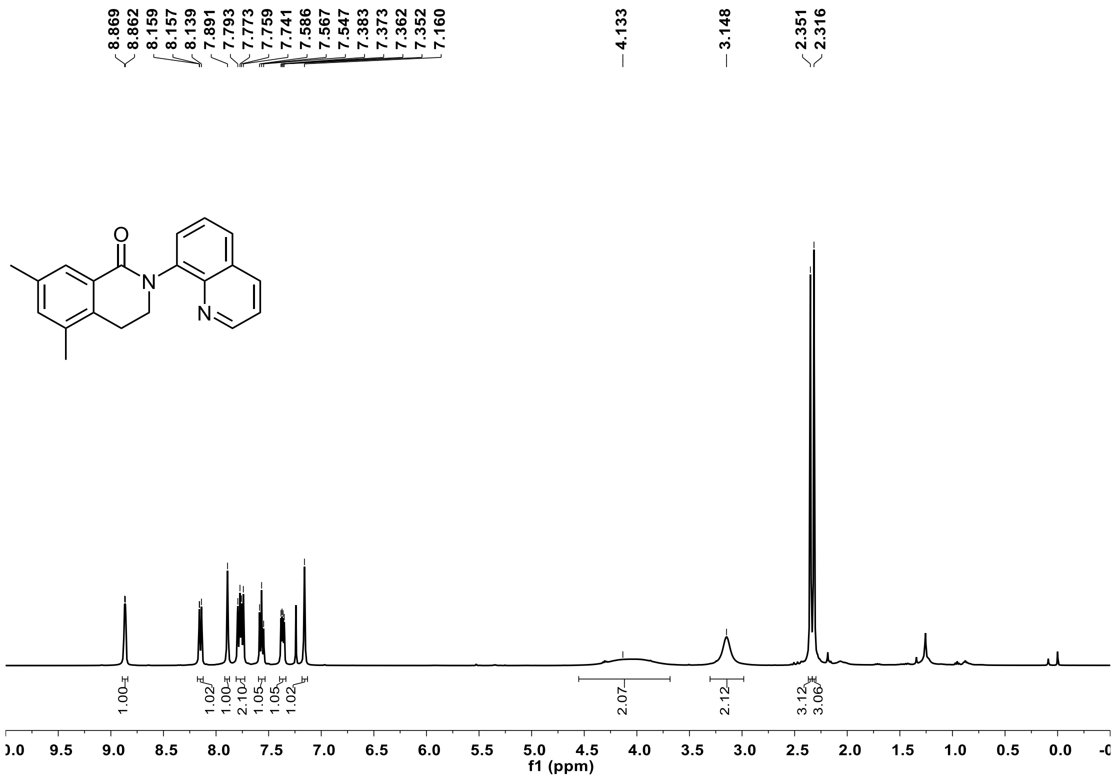

Supplementary Figure 8. ^{1}H NMR (400 MHz, $CDCl_3$) spectrum of 3ca

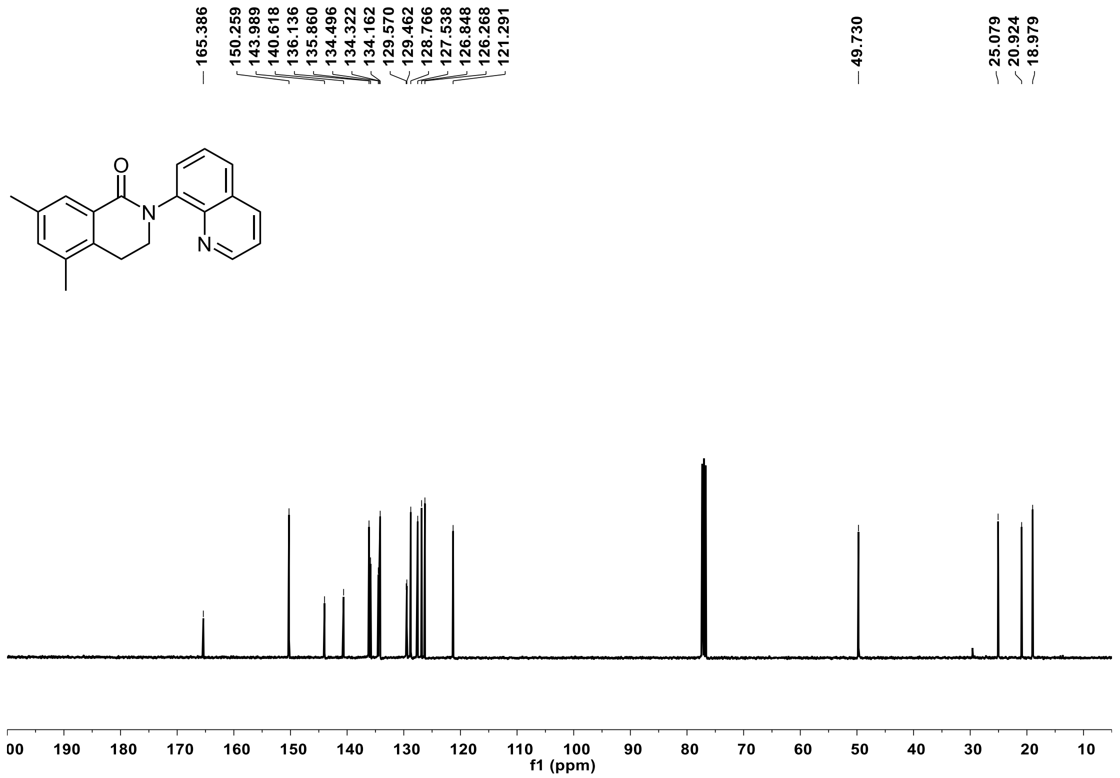

Supplementary Figure 9. ^{13}C NMR (101 MHz, $CDCl_3$) spectrum of 3ca

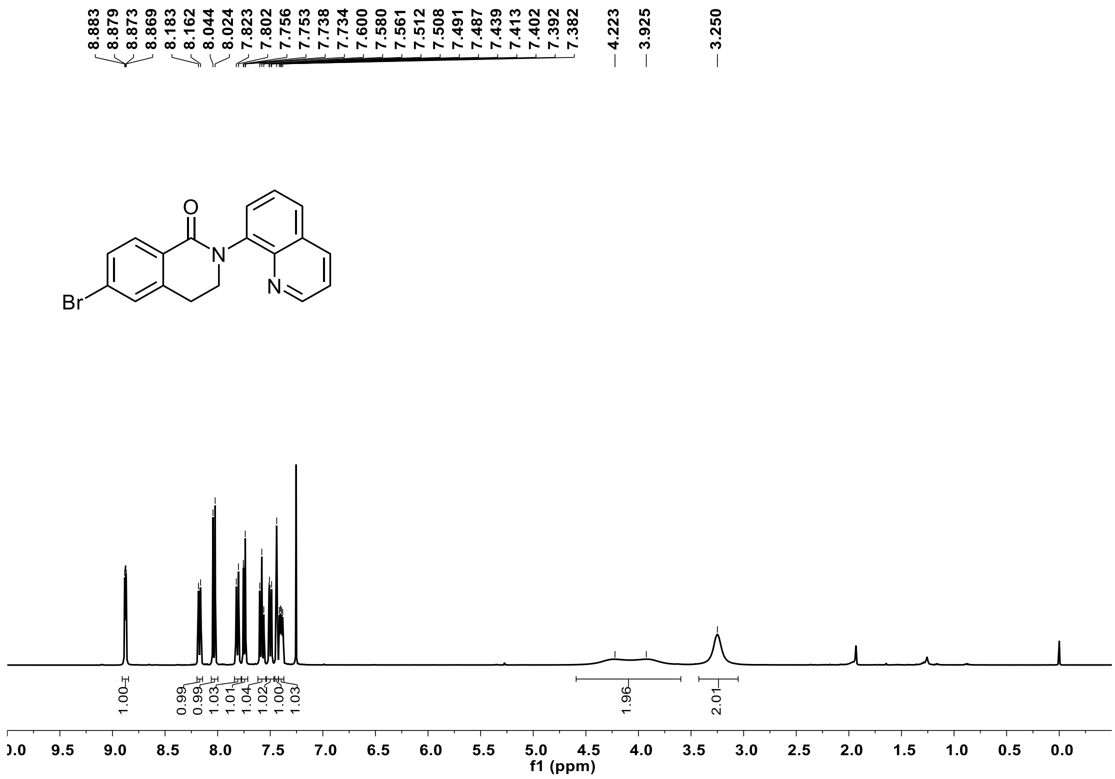

Supplementary Figure 10. ^{1}H NMR (400 MHz, $CDCl_3$) spectrum of 3da

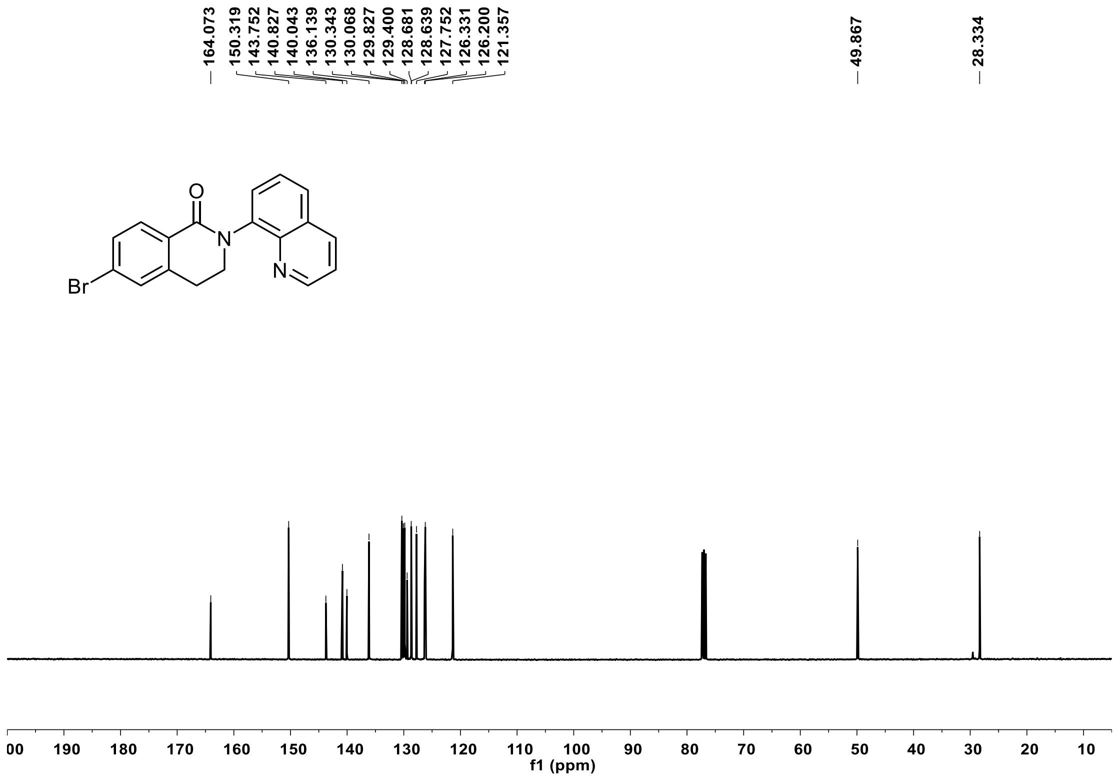

Supplementary Figure 11. ^{13}C NMR (101 MHz, $CDCl_3$) spectrum of 3da

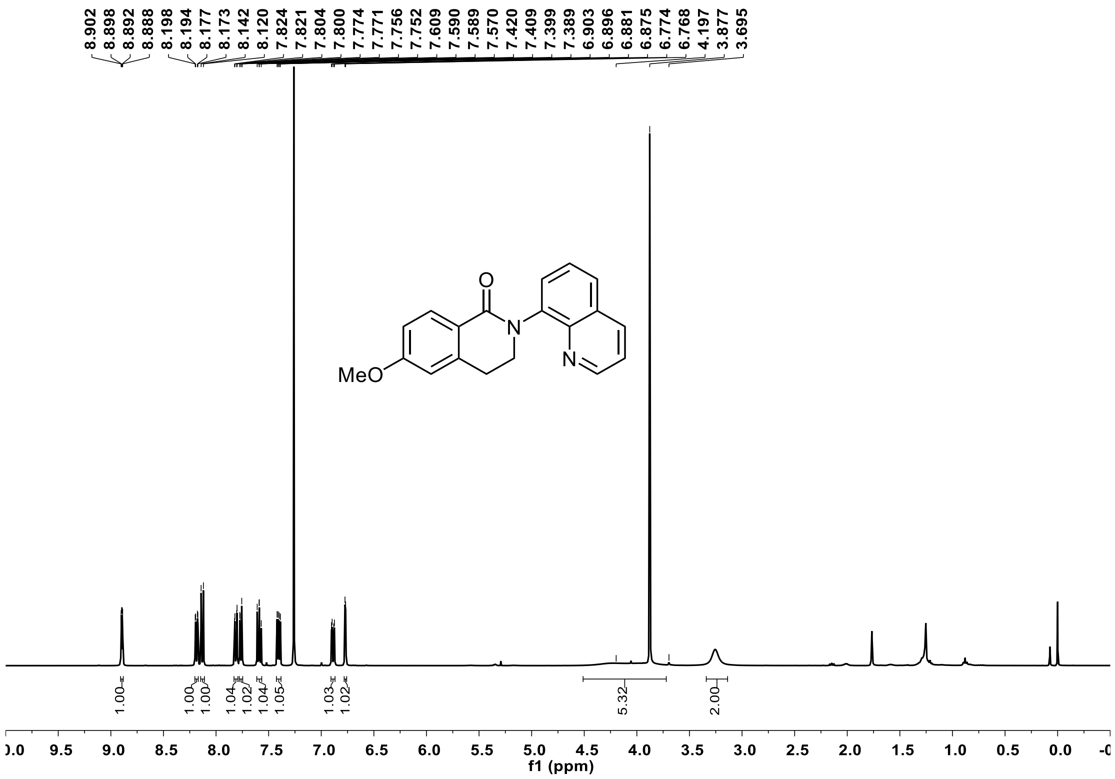

Supplementary Figure 12. ^{1}H NMR (400 MHz, $CDCl_3$) spectrum of 3ea

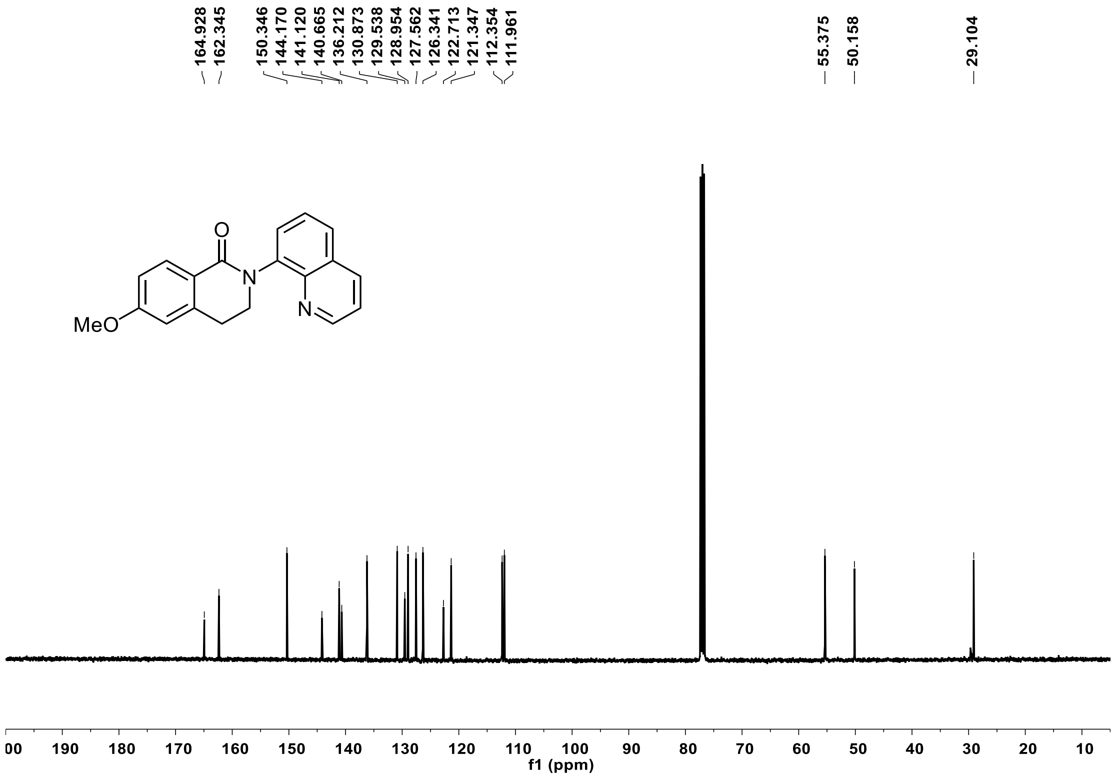

Supplementary Figure 13. ^{13}C NMR (101 MHz, $CDCl_3$) spectrum of 3ea

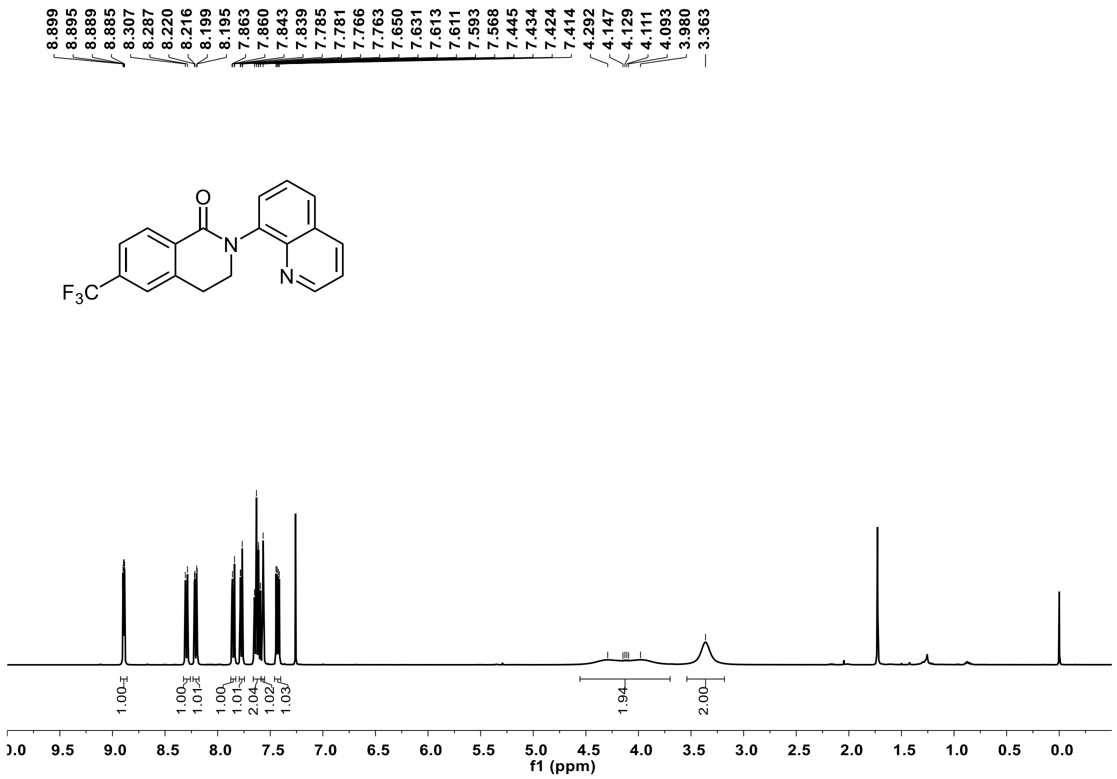

Supplementary Figure 14. ^{1}H NMR (400 MHz, $CDCl_3$) spectrum of 3fa

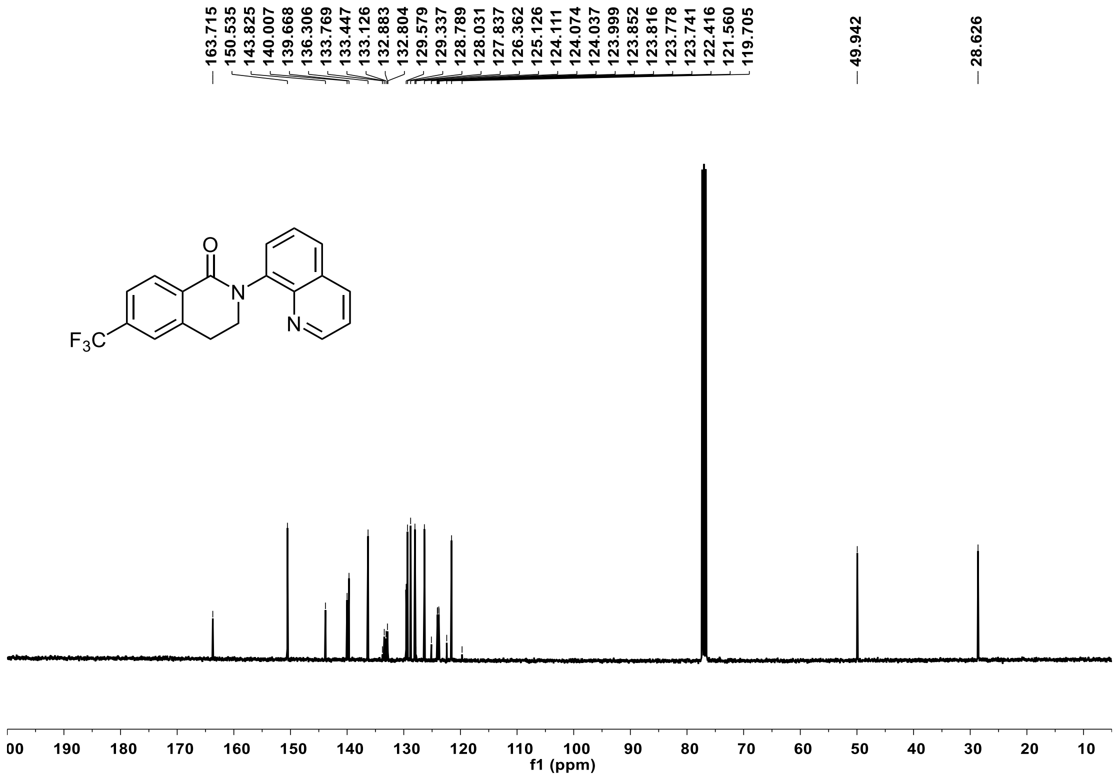

Supplementary Figure 15. ^{13}C NMR (101 MHz, $CDCl_3$) spectrum of 3fa

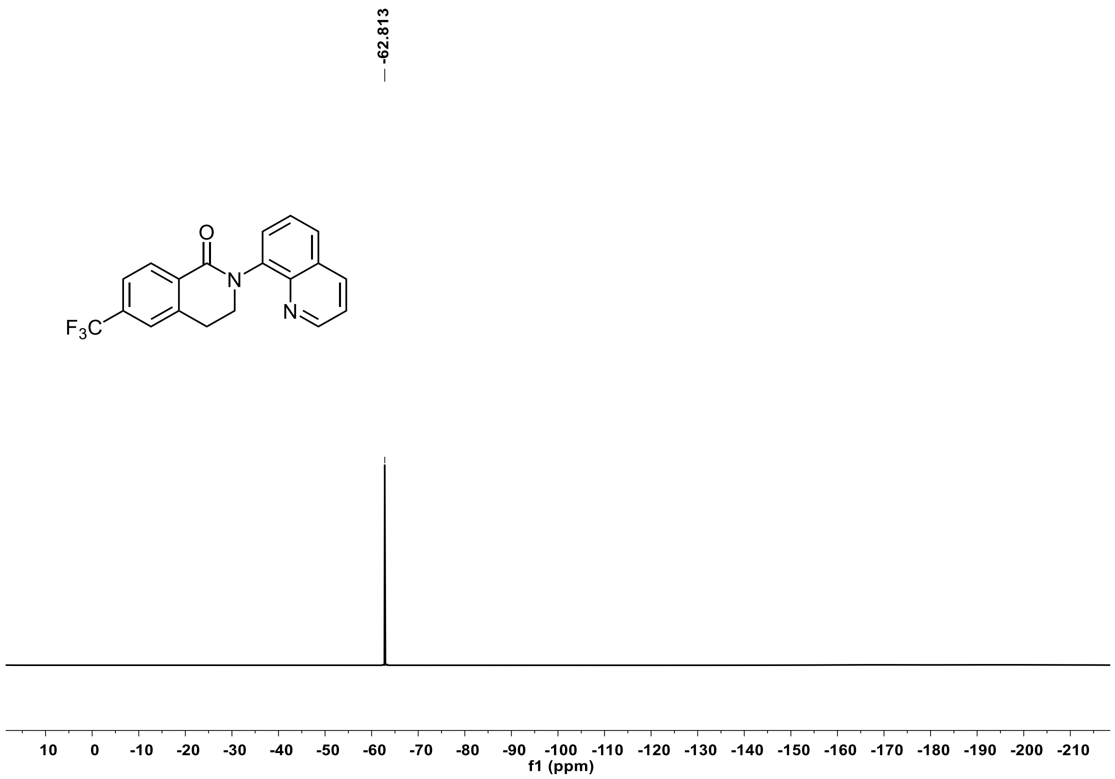

Supplementary Figure 16. ^{19}F NMR (376 MHz, $CDCl_3$) spectrum of 3fa

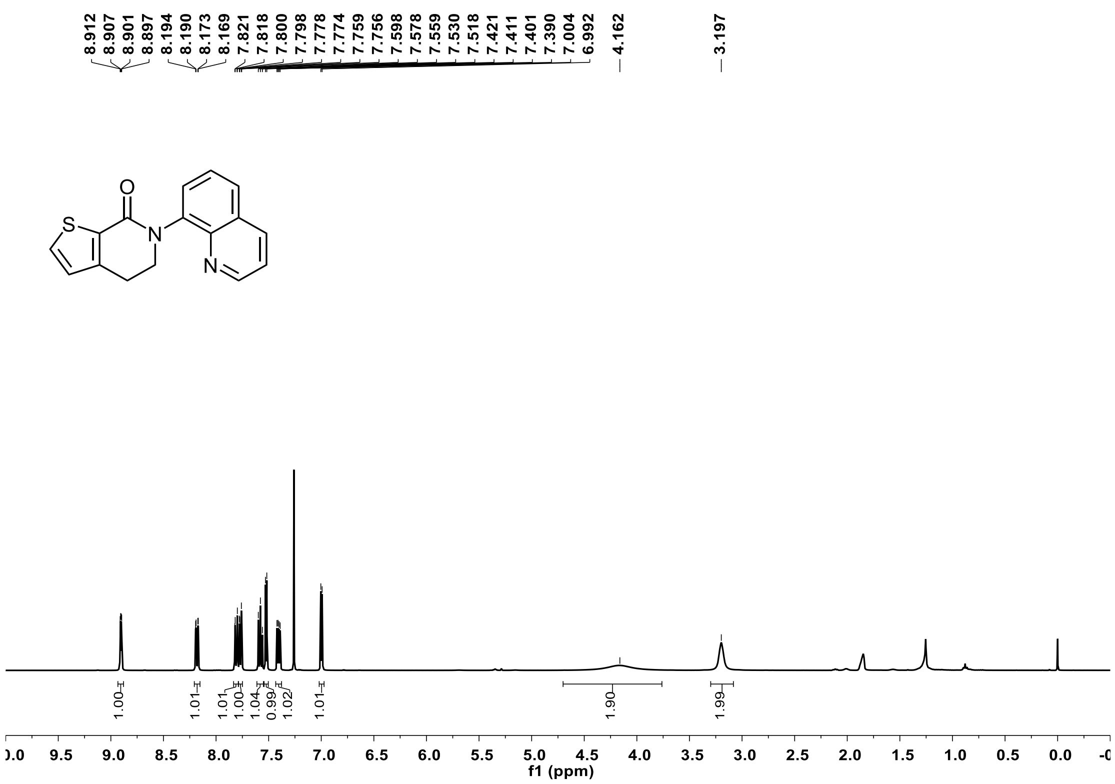

Supplementary Figure 17. ^{1}H NMR (400 MHz, $CDCl_3$) spectrum of 3ga

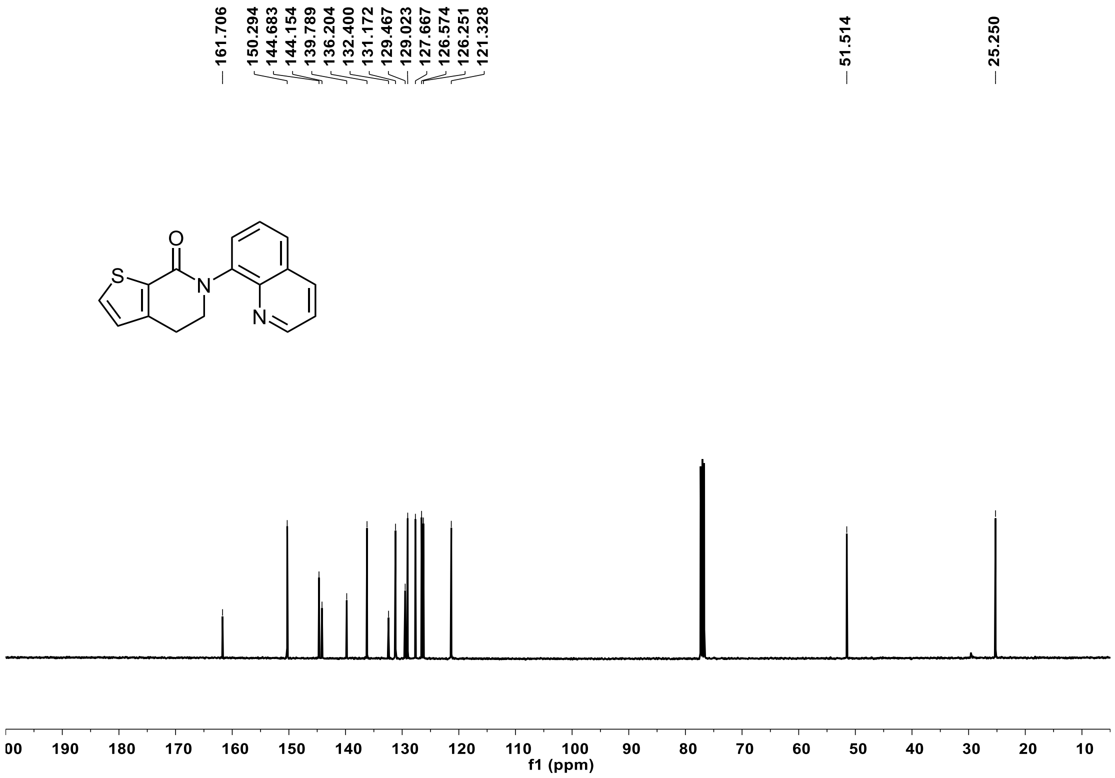

Supplementary Figure 18. ^{13}C NMR (101 MHz, $CDCl_3$) spectrum of 3ga

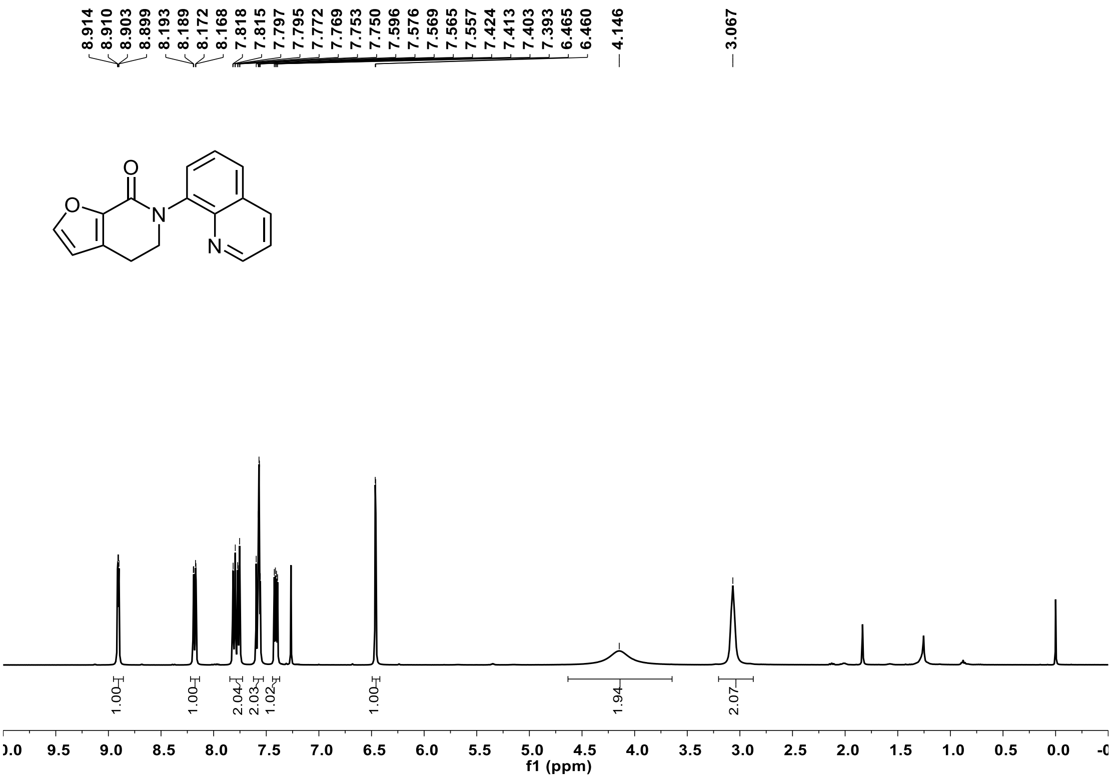

Supplementary Figure 19. ^{1}H NMR (400 MHz, $CDCl_3$) spectrum of 3ha

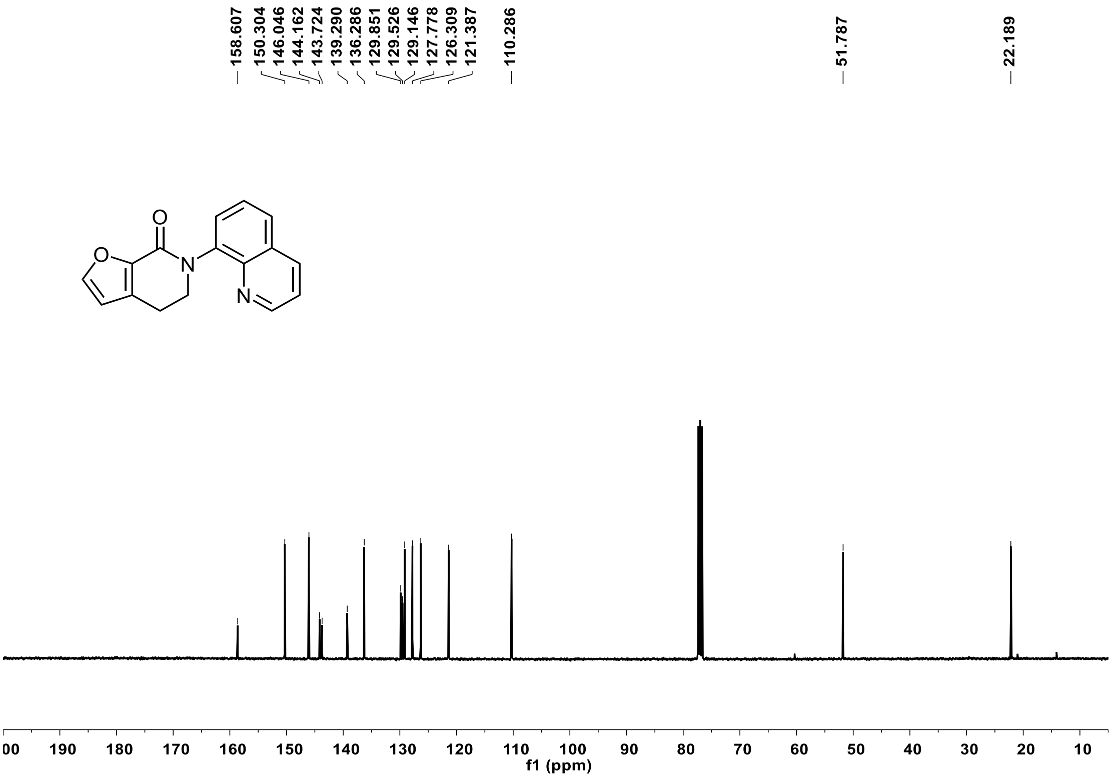

Supplementary Figure 20. ^{13}C NMR (101 MHz, $CDCl_3$) spectrum of 3ha

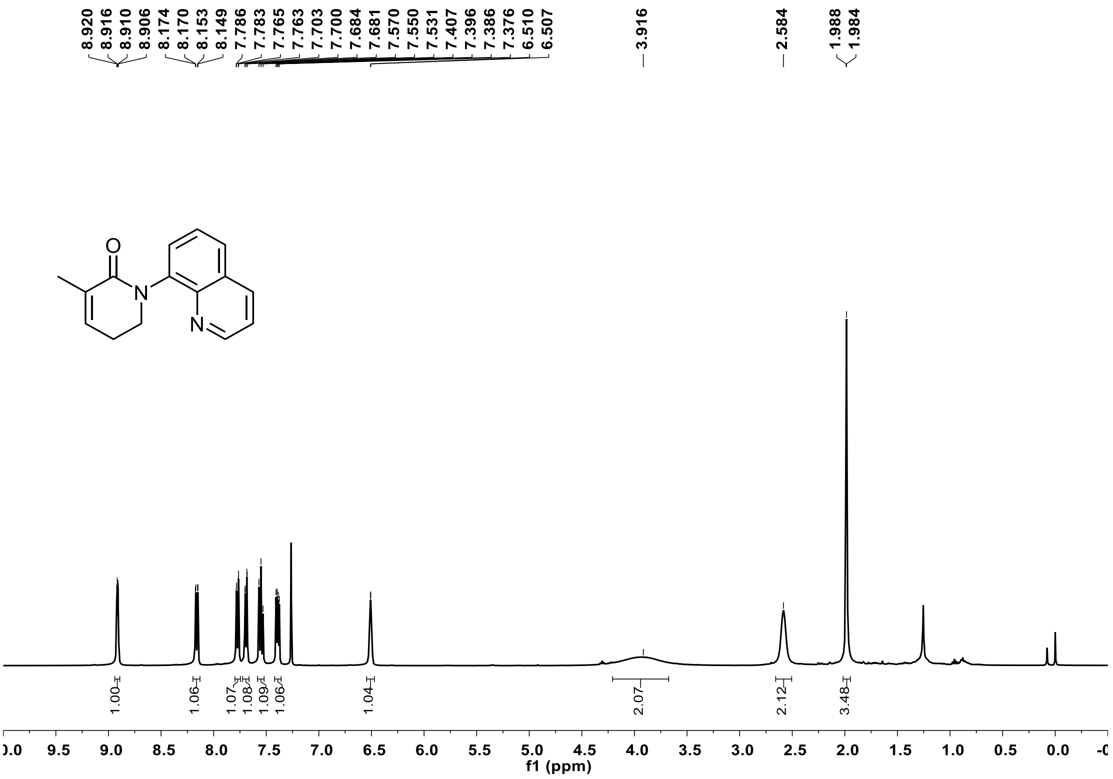

Supplementary Figure 21. ^{1}H NMR (400 MHz, $CDCl_3$) spectrum of 3ia

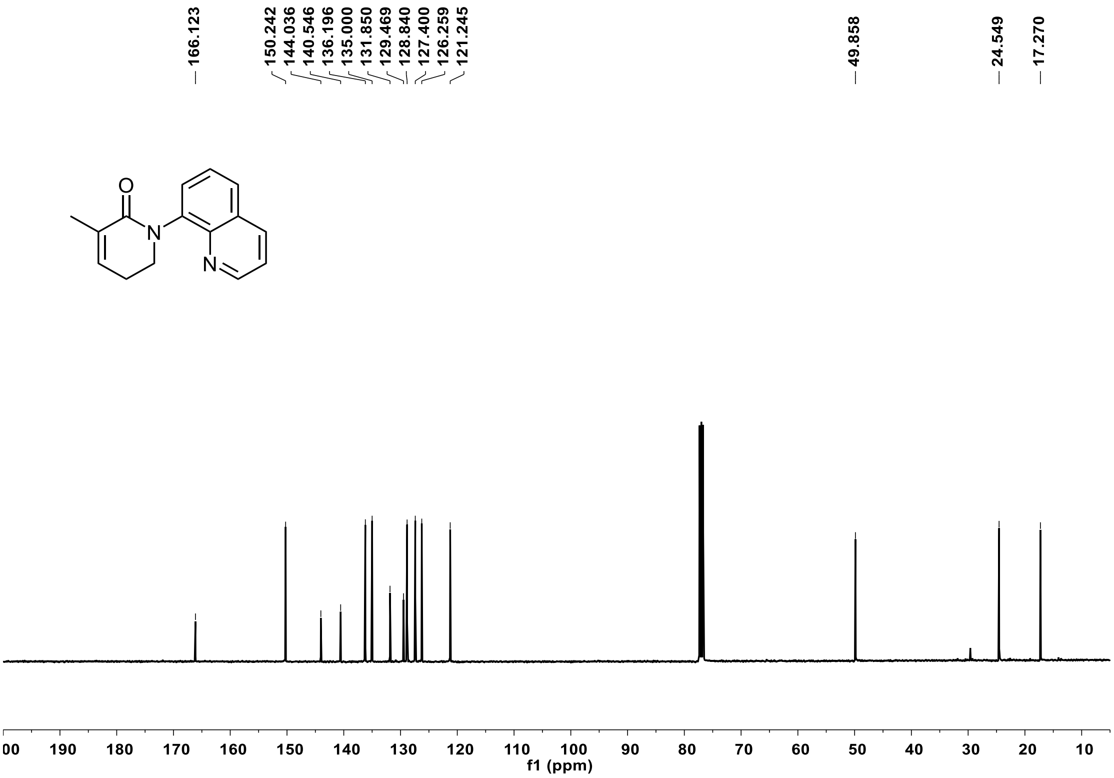

Supplementary Figure 22. ^{13}C NMR (101 MHz, $CDCl_3$) spectrum of 3ia

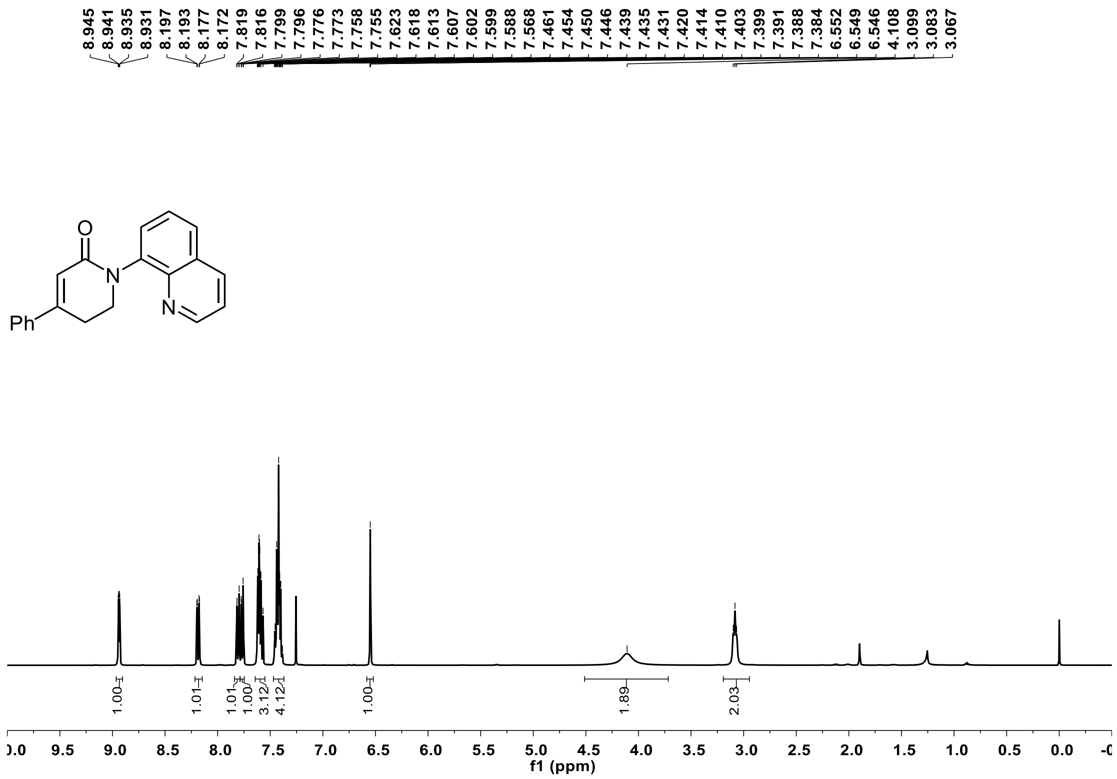

Supplementary Figure 23. ^{1}H **NMR (400 MHz,** $CDCl_3$**) spectrum of 3ja**

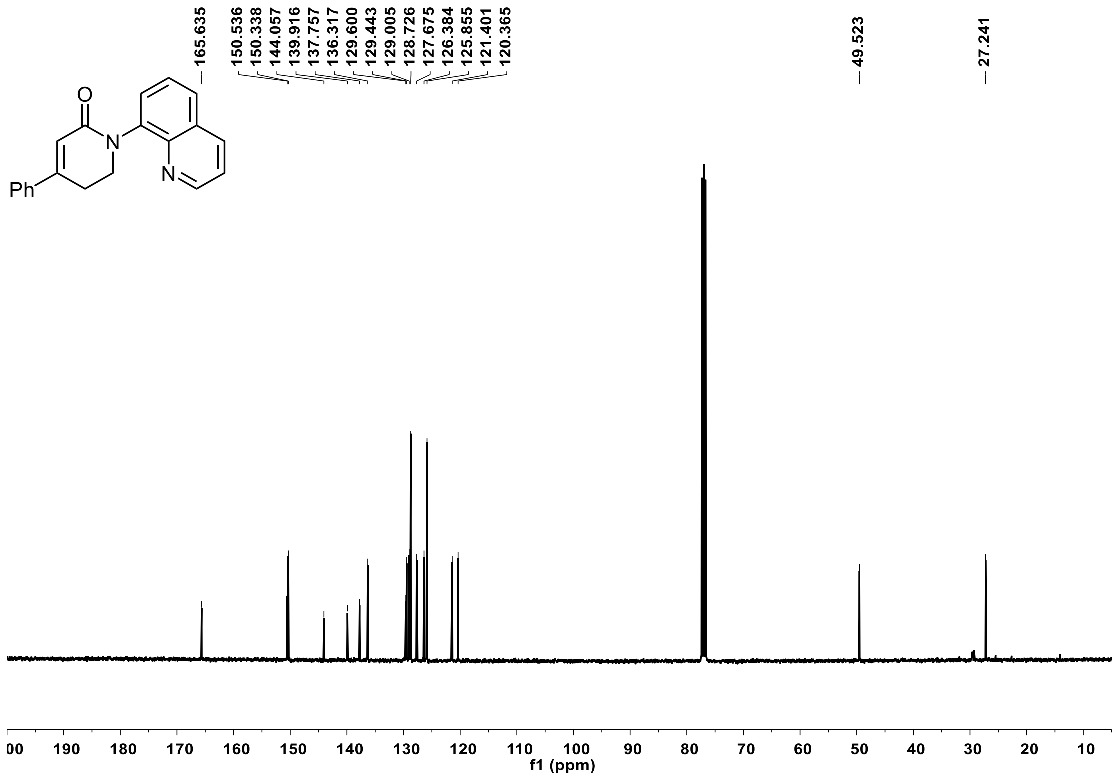

Supplementary Figure 24. ^{13}C **NMR (101 MHz,** $CDCl_3$**) spectrum of 3ja**

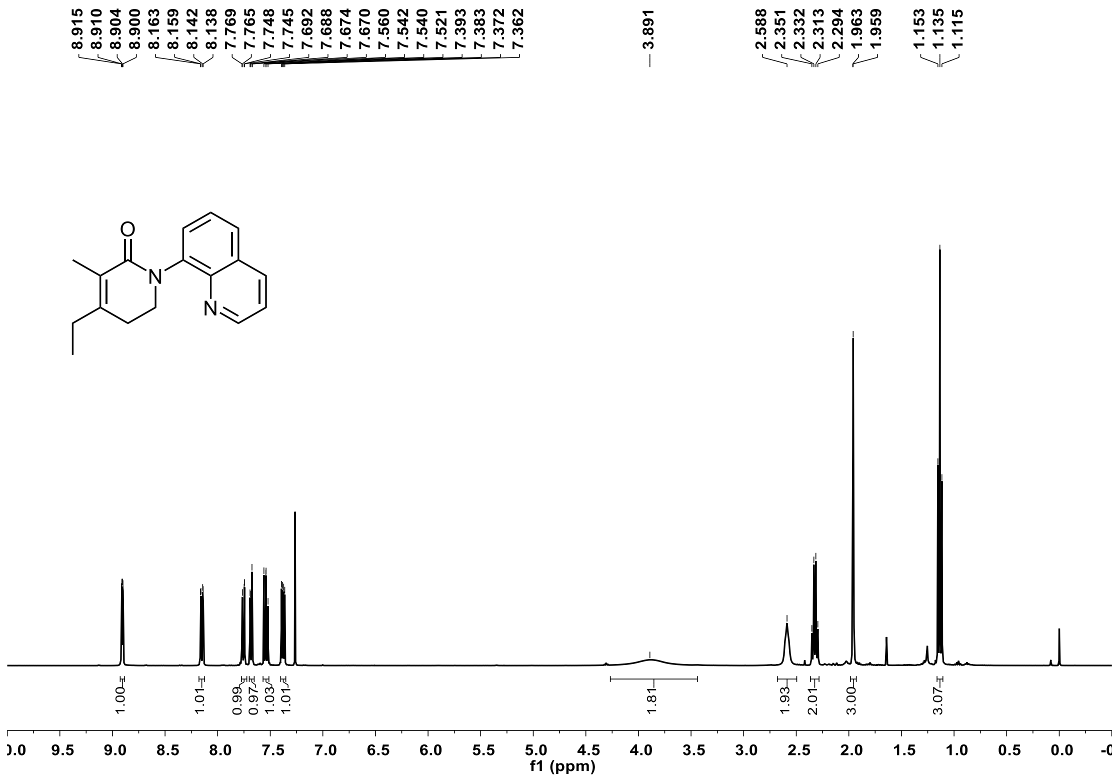

Supplementary Figure 25. ^{1}H NMR (400 MHz, $CDCl_3$) spectrum of 3ka

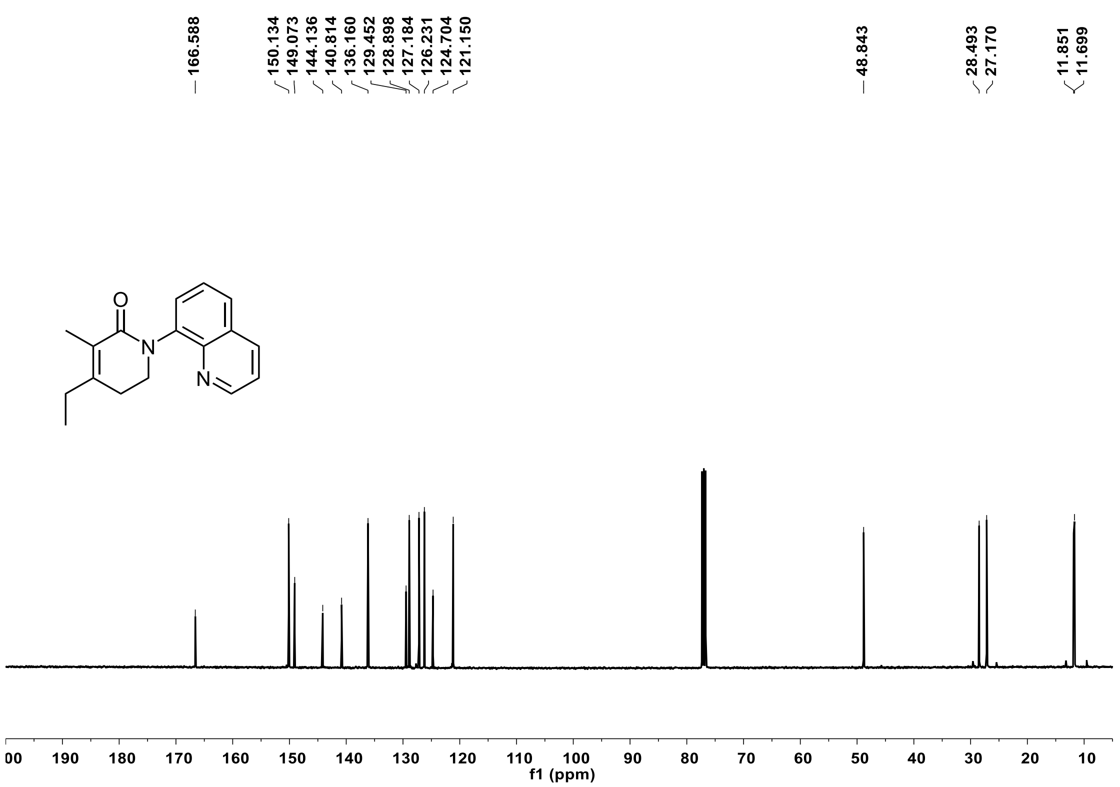

Supplementary Figure 26. ^{13}C NMR (101 MHz, $CDCl_3$) spectrum of 3ka

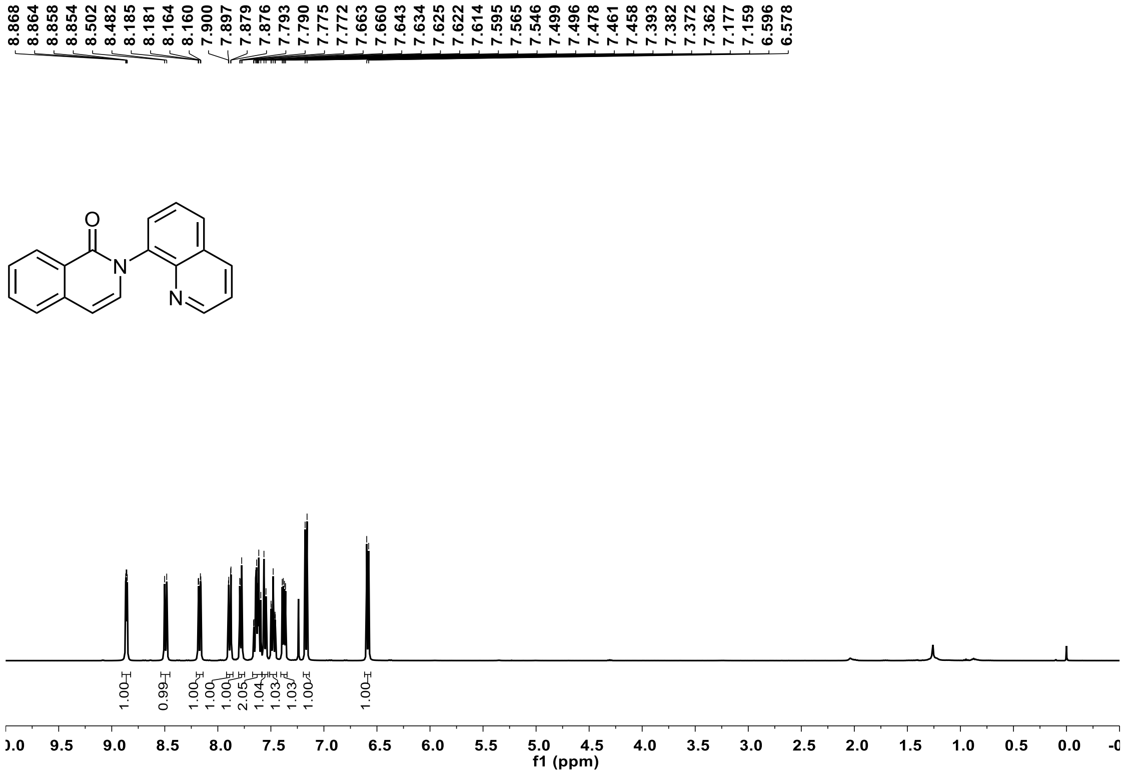

Supplementary Figure 27. ^{1}H NMR (400 MHz, $CDCl_3$) spectrum of 3ab

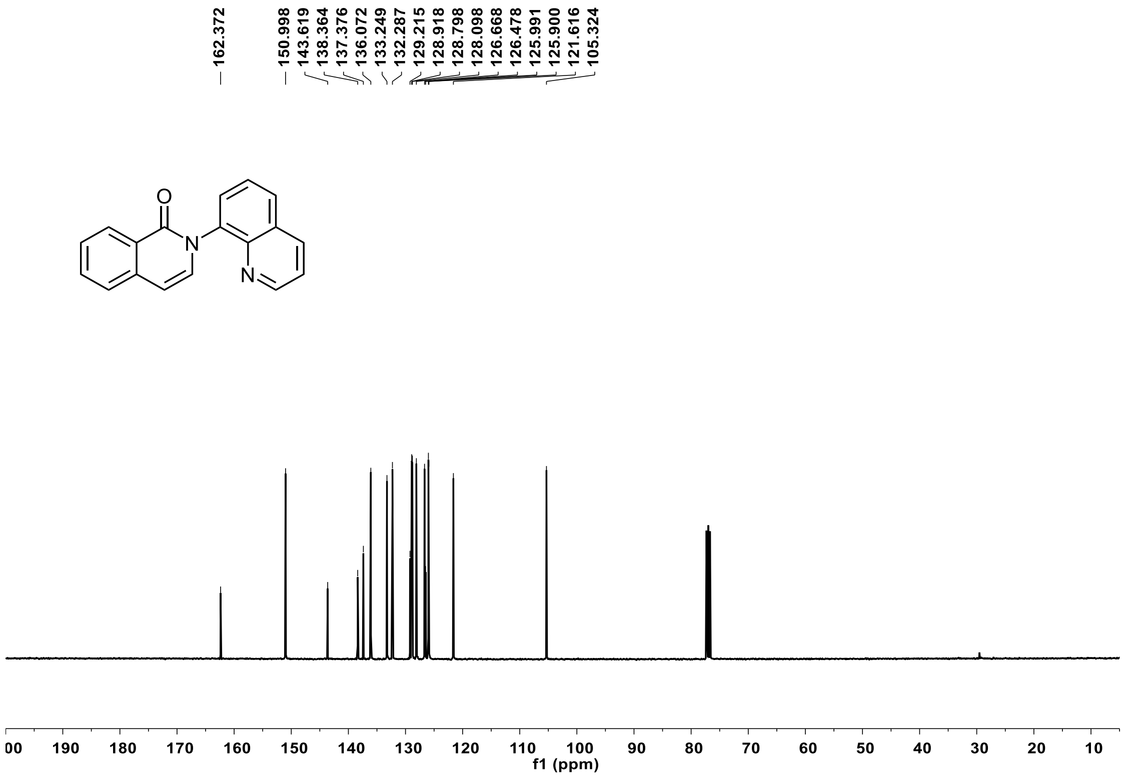

Supplementary Figure 28. ^{13}C NMR (101 MHz, $CDCl_3$) spectrum of 3ab

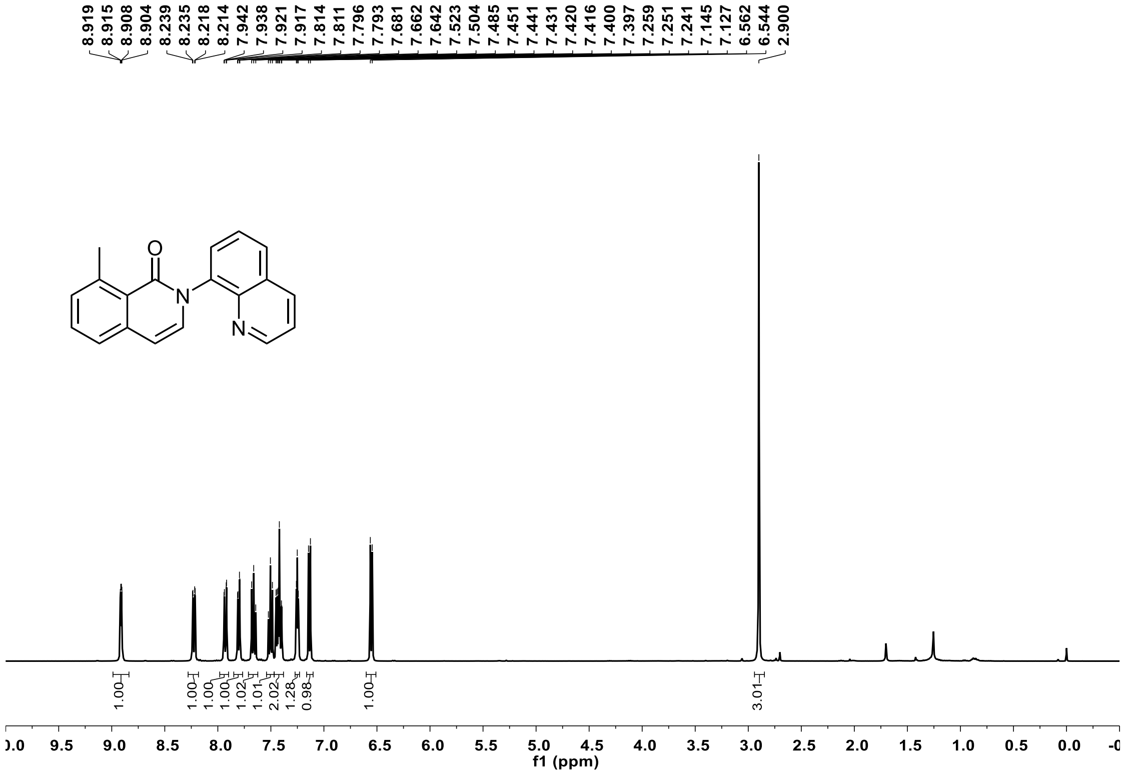

Supplementary Figure 29. ^{1}H NMR (400 MHz, $CDCl_3$) spectrum of 3bb

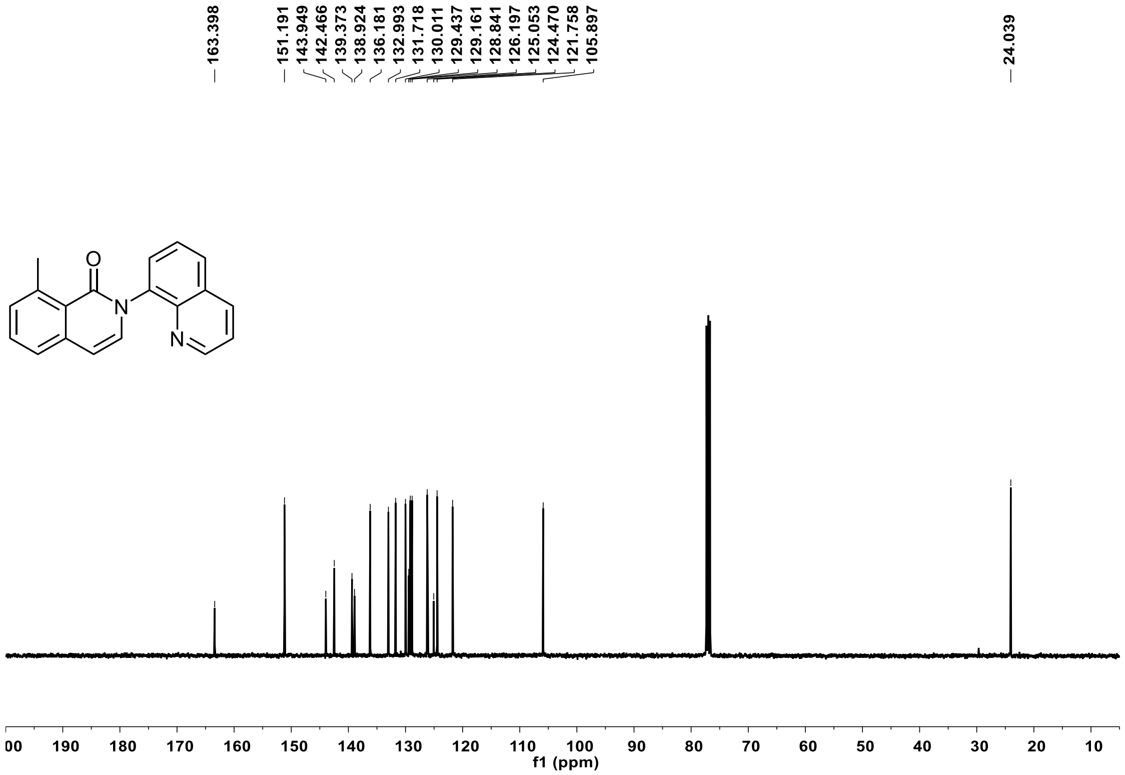

Supplementary Figure 30. ^{13}C NMR (101 MHz, $CDCl_3$) spectrum of 3bb

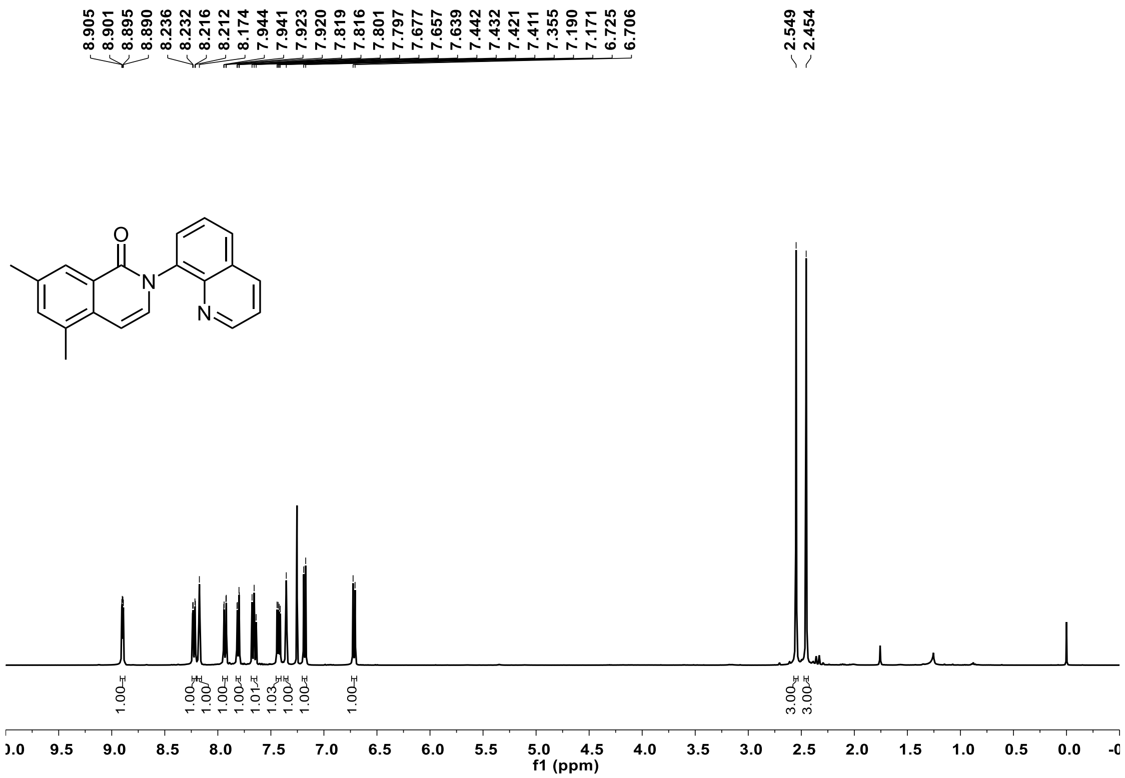

Supplementary Figure 31. ^{1}H NMR (400 MHz, $CDCl_3$) spectrum of 3cb

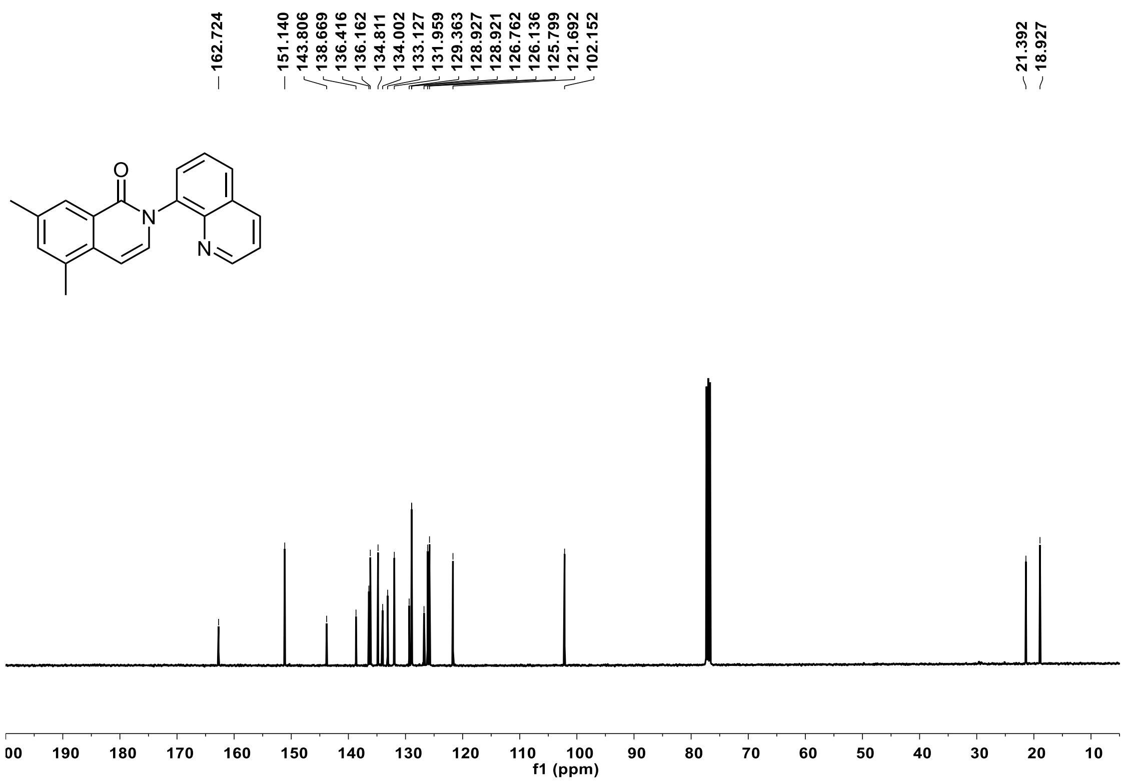

Supplementary Figure 32. ^{13}C NMR (101 MHz, $CDCl_3$) spectrum of 3cb

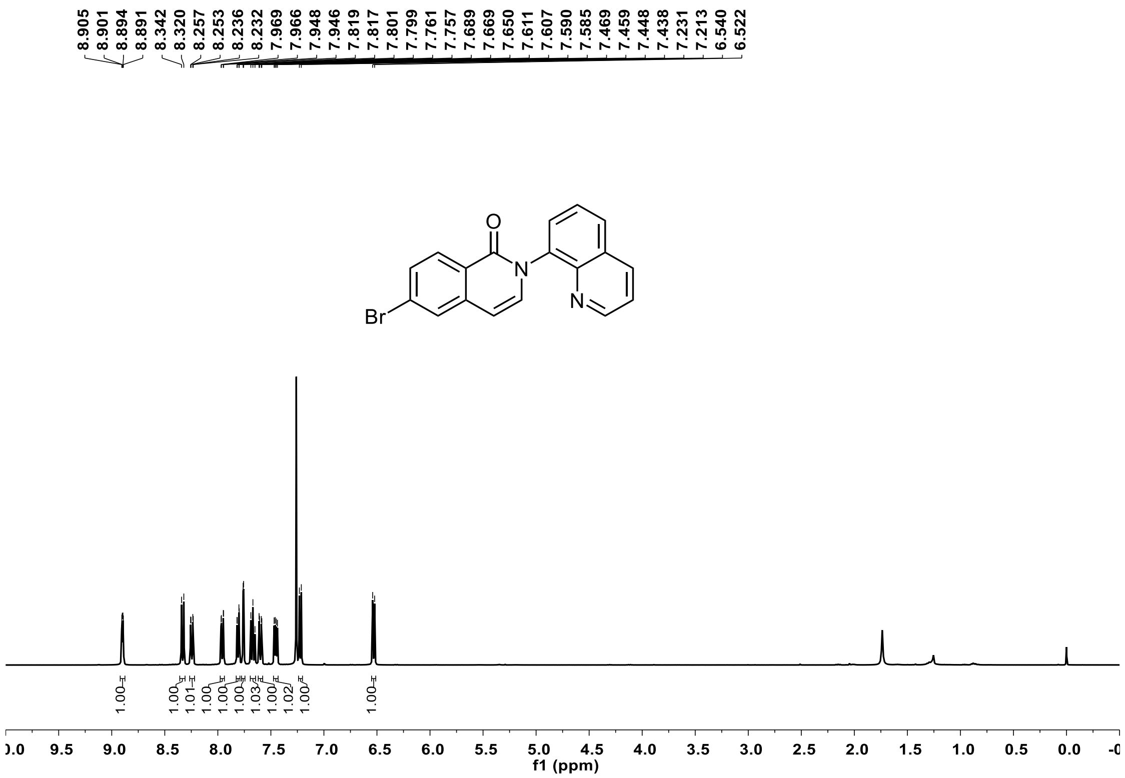

Supplementary Figure 33. ^{1}H NMR (400 MHz, $CDCl_3$) spectrum of 3db

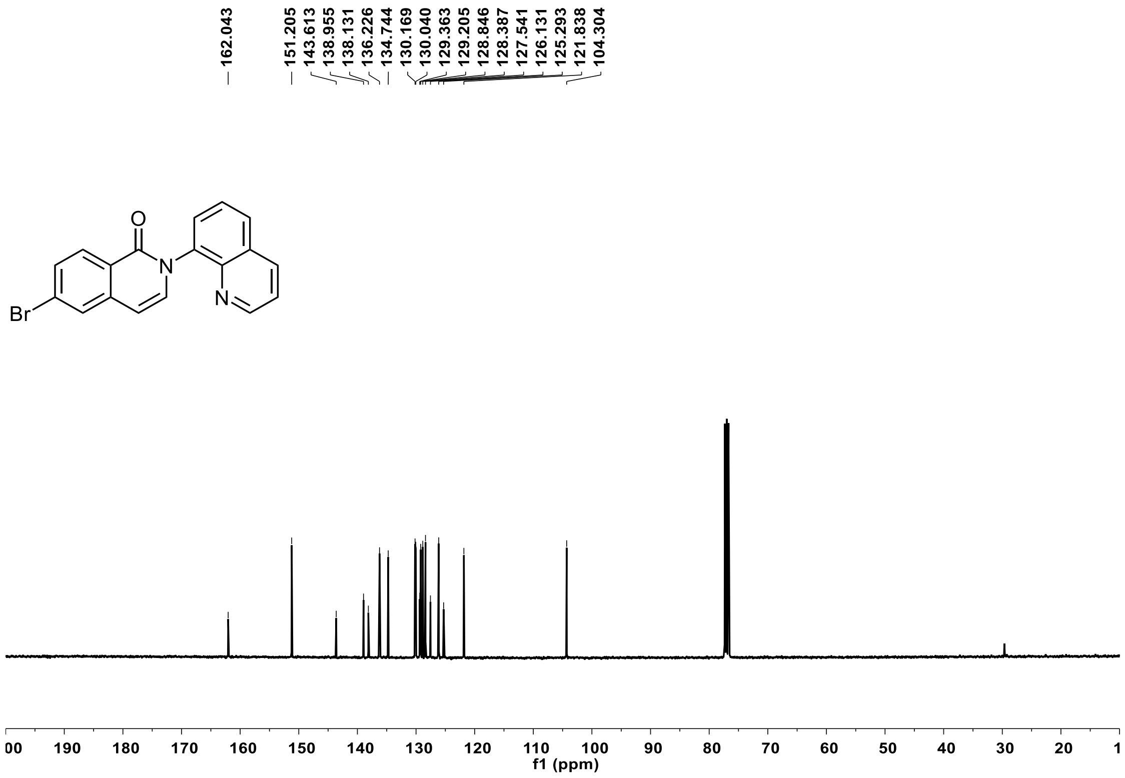

Supplementary Figure 34. ^{13}C NMR (101 MHz, $CDCl_3$) spectrum of 3db

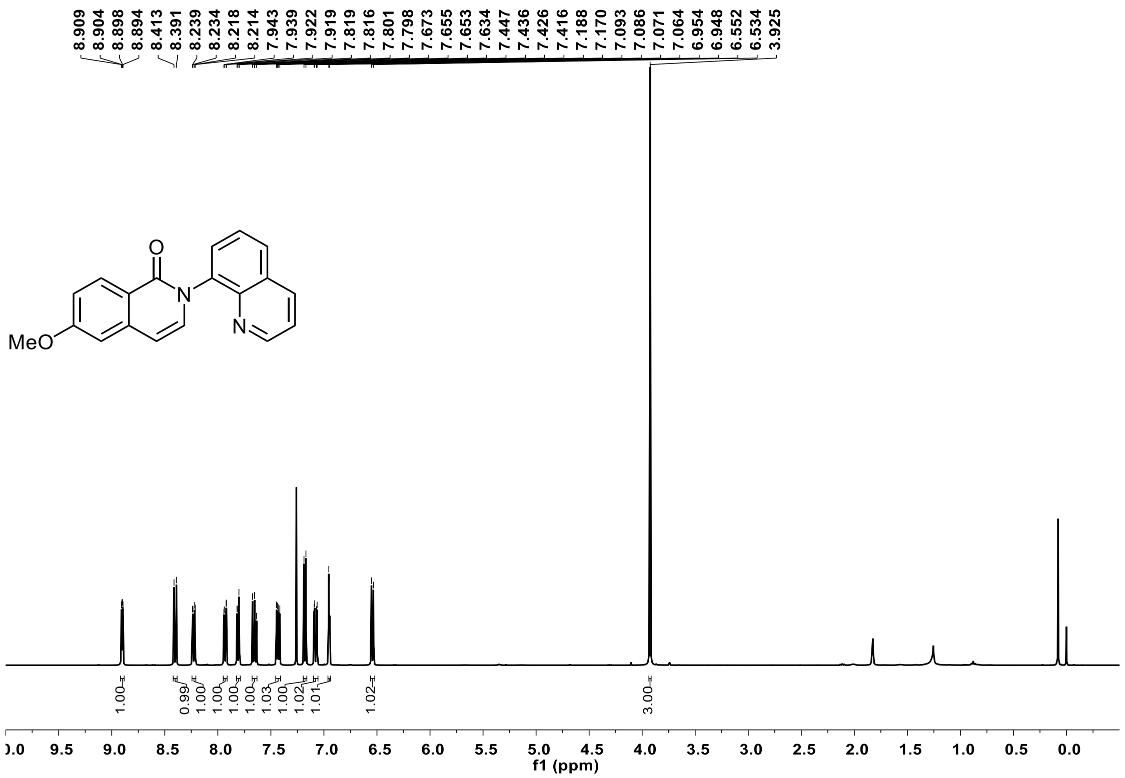

Supplementary Figure 35. ^{1}H NMR (400 MHz, $CDCl_3$) spectrum of 3eb

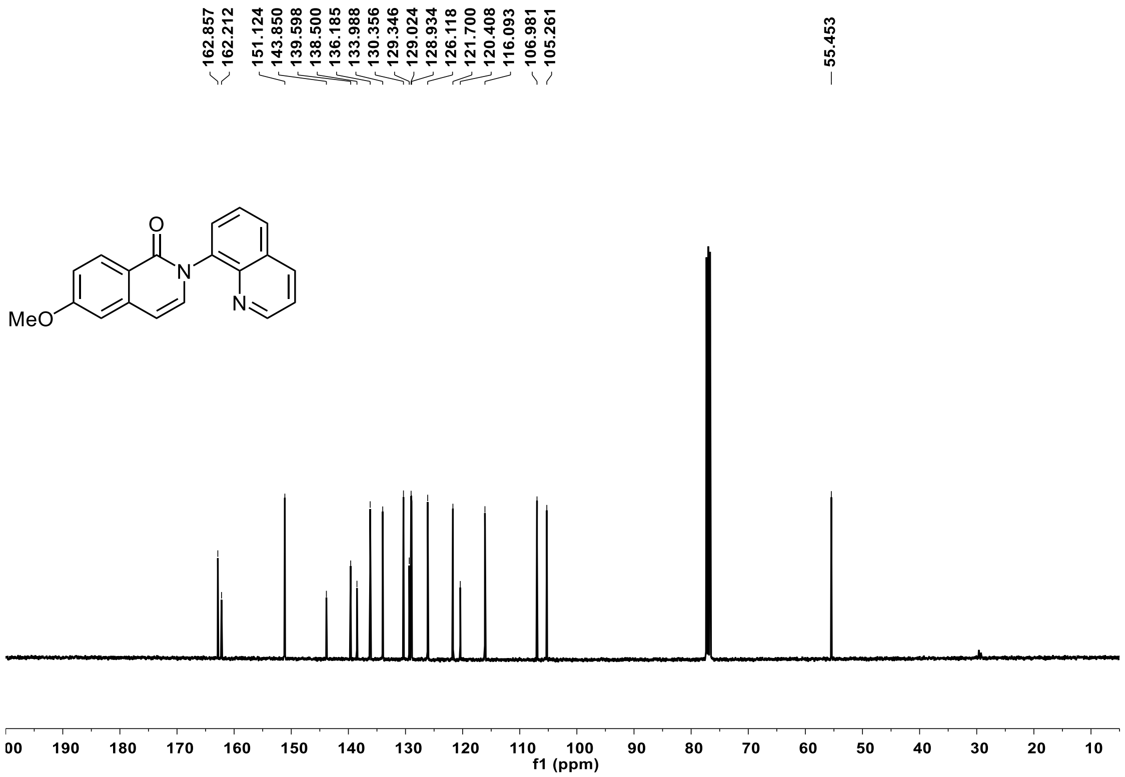

Supplementary Figure 36. ^{13}C NMR (101 MHz, $CDCl_3$) spectrum of 3eb

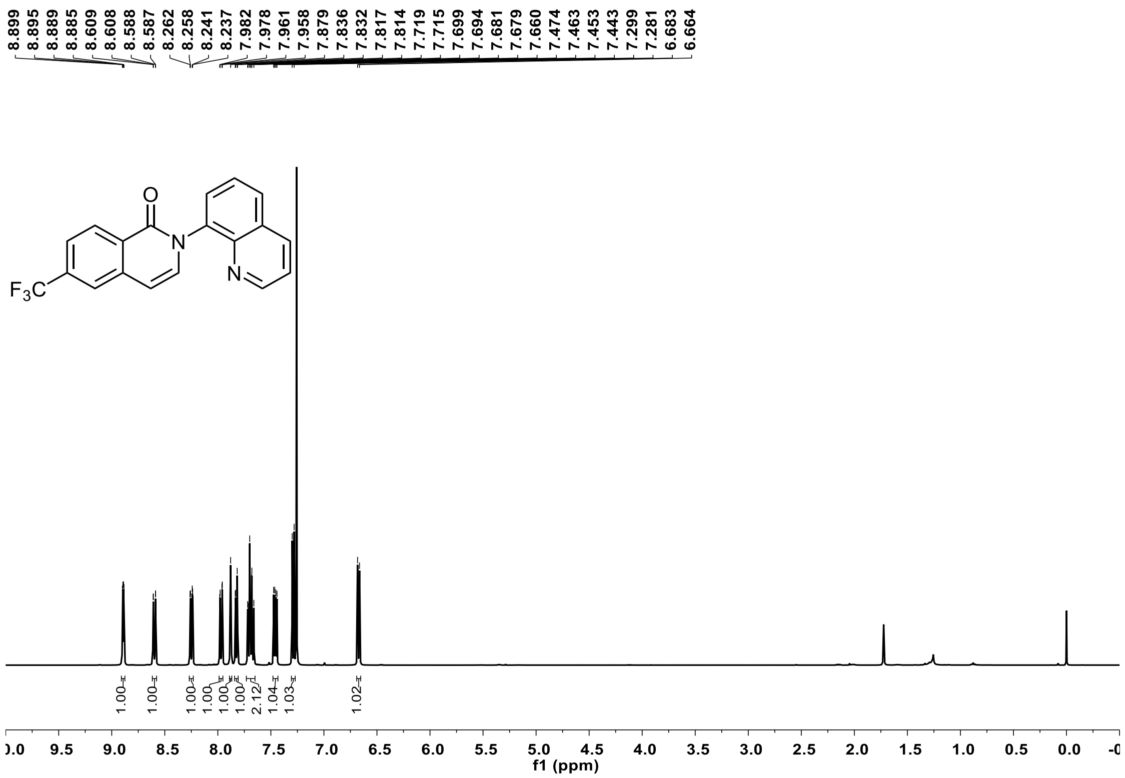

Supplementary Figure 37. ^{1}H NMR (400 MHz, $CDCl_3$) spectrum of 3fb

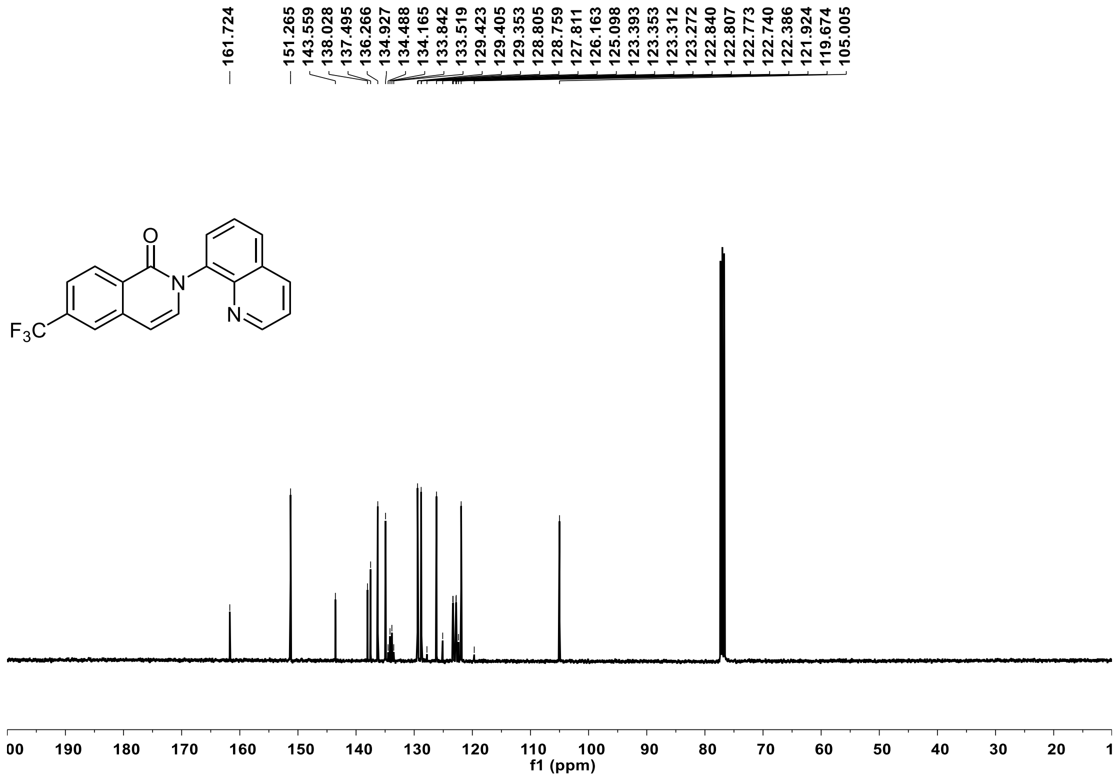

Supplementary Figure 38. ^{13}C NMR (101 MHz, $CDCl_3$) spectrum of 3fb

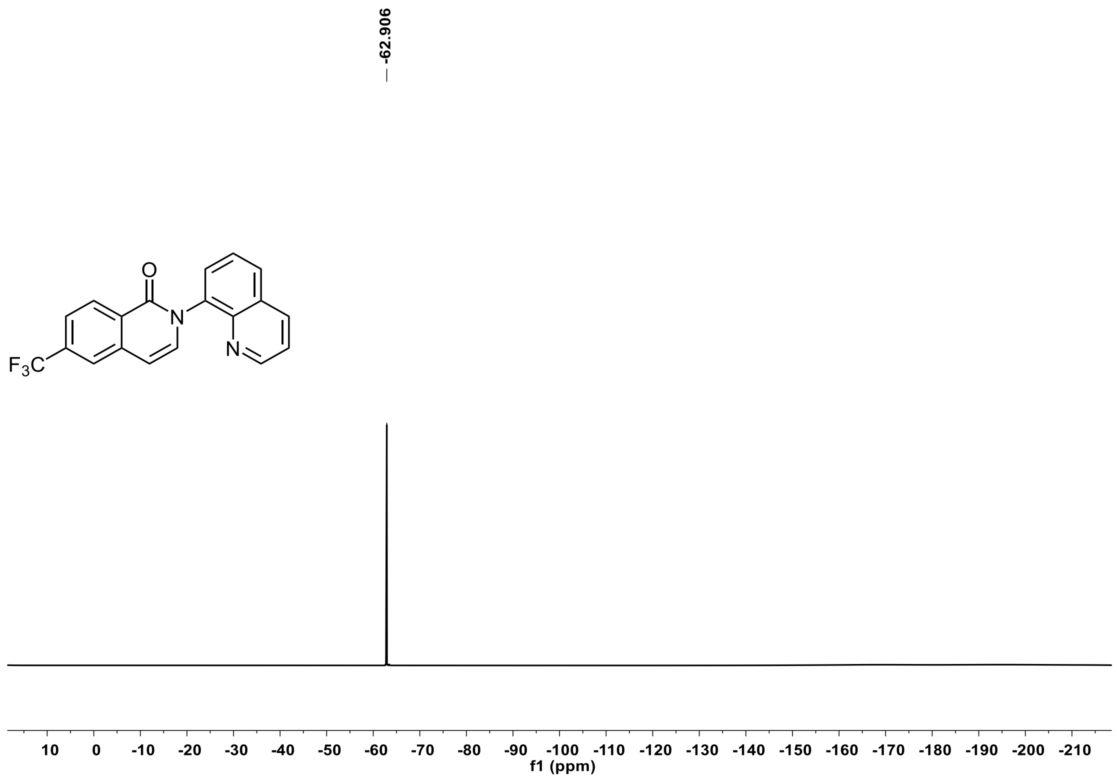

Supplementary Figure 39. **^{19}F NMR (376 MHz, $CDCl_3$) spectrum of 3fb**

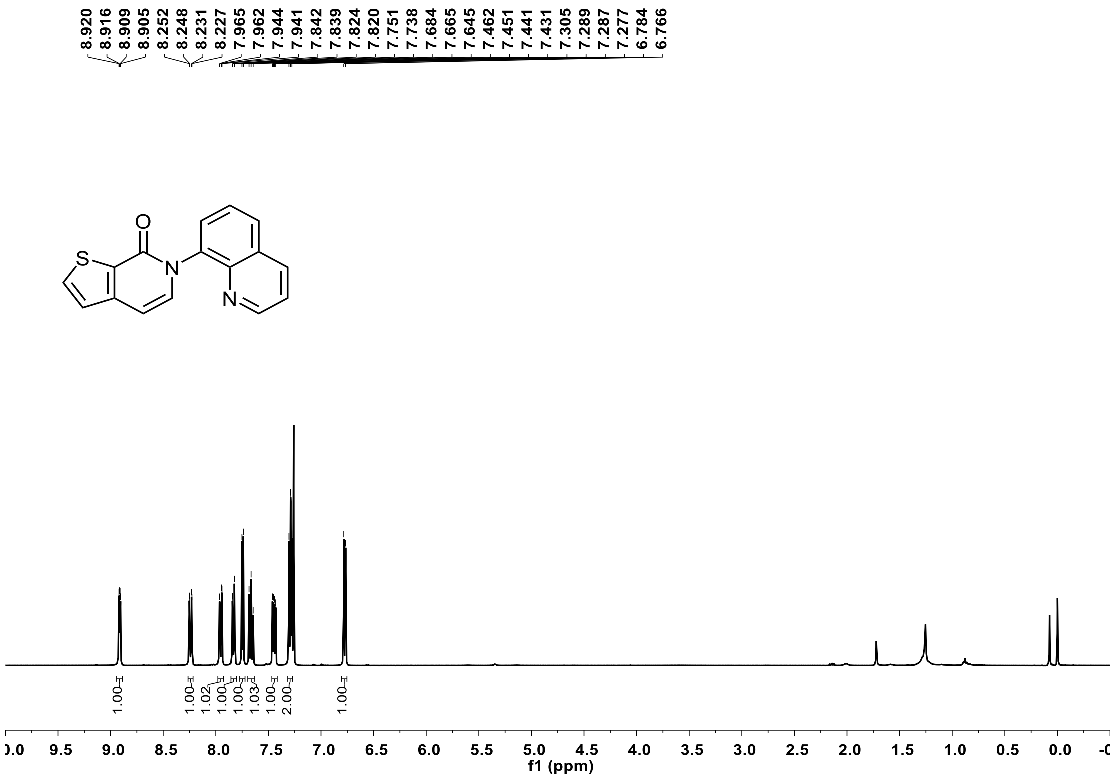

Supplementary Figure 40. ^{1}H NMR (400 MHz, $CDCl_3$) spectrum of 3gb

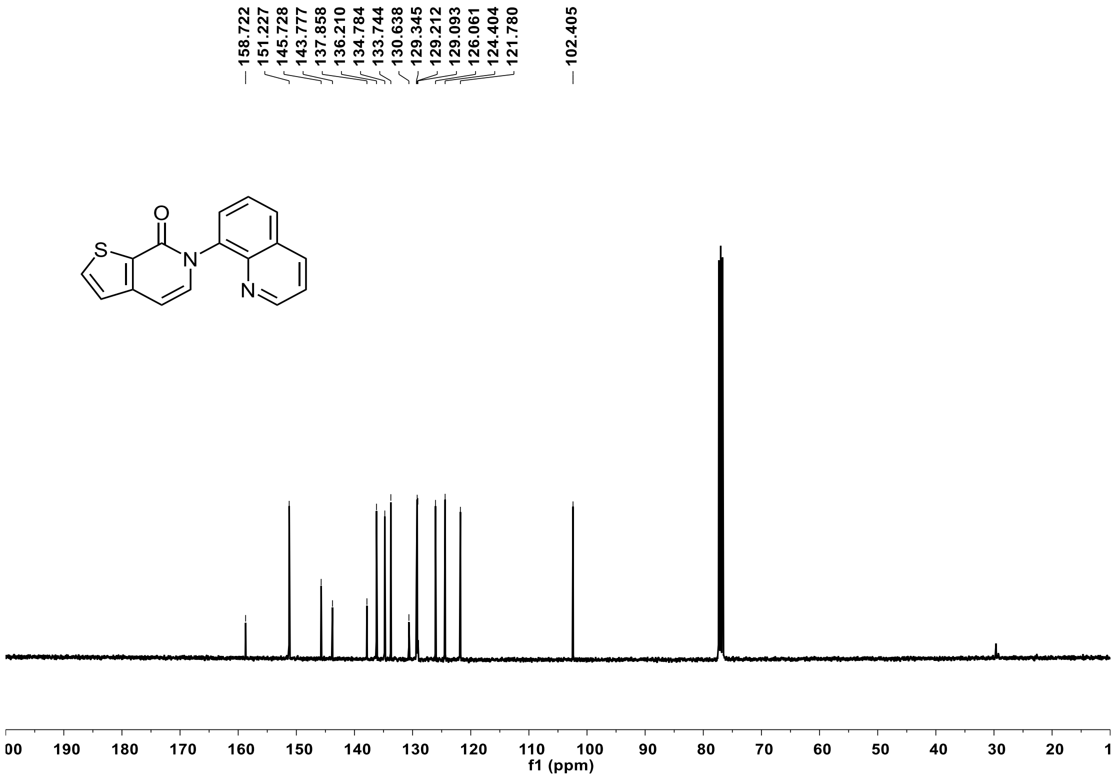

Supplementary Figure 41. ^{13}C NMR (101 MHz, $CDCl_3$) spectrum of 3gb

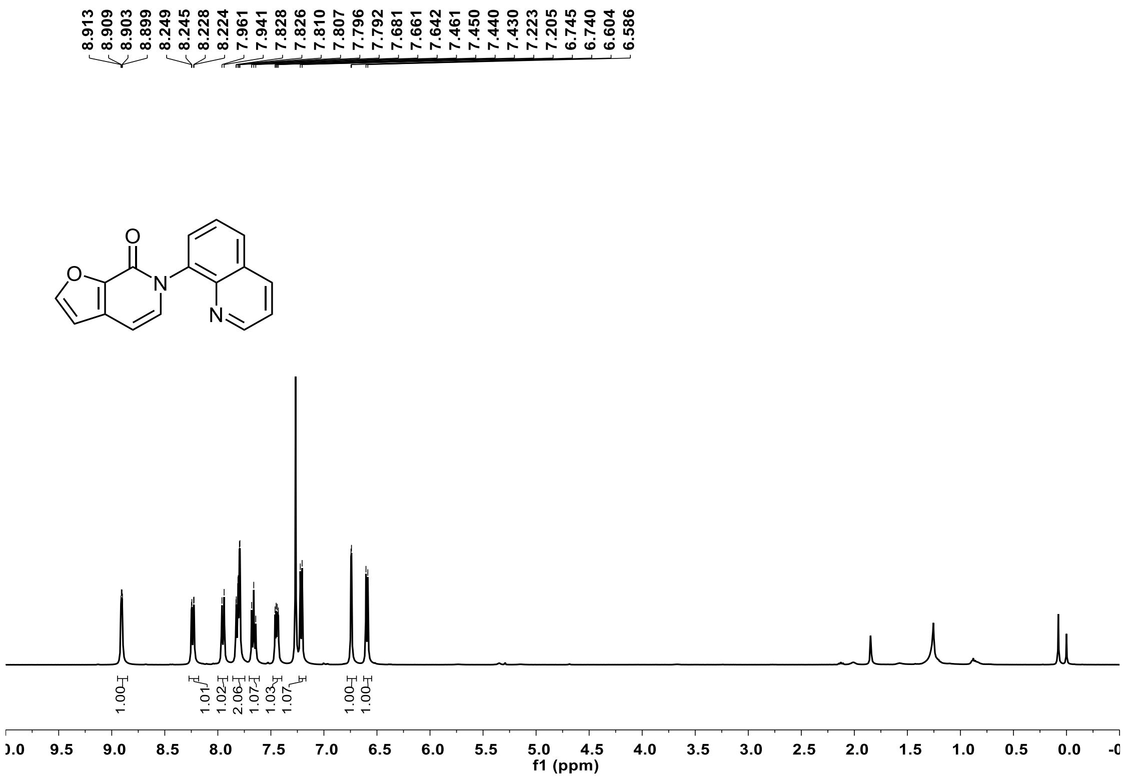

Supplementary Figure 42. ^{1}**H NMR (400 MHz, CDCl**$_{3}$**) spectrum of 3hb**

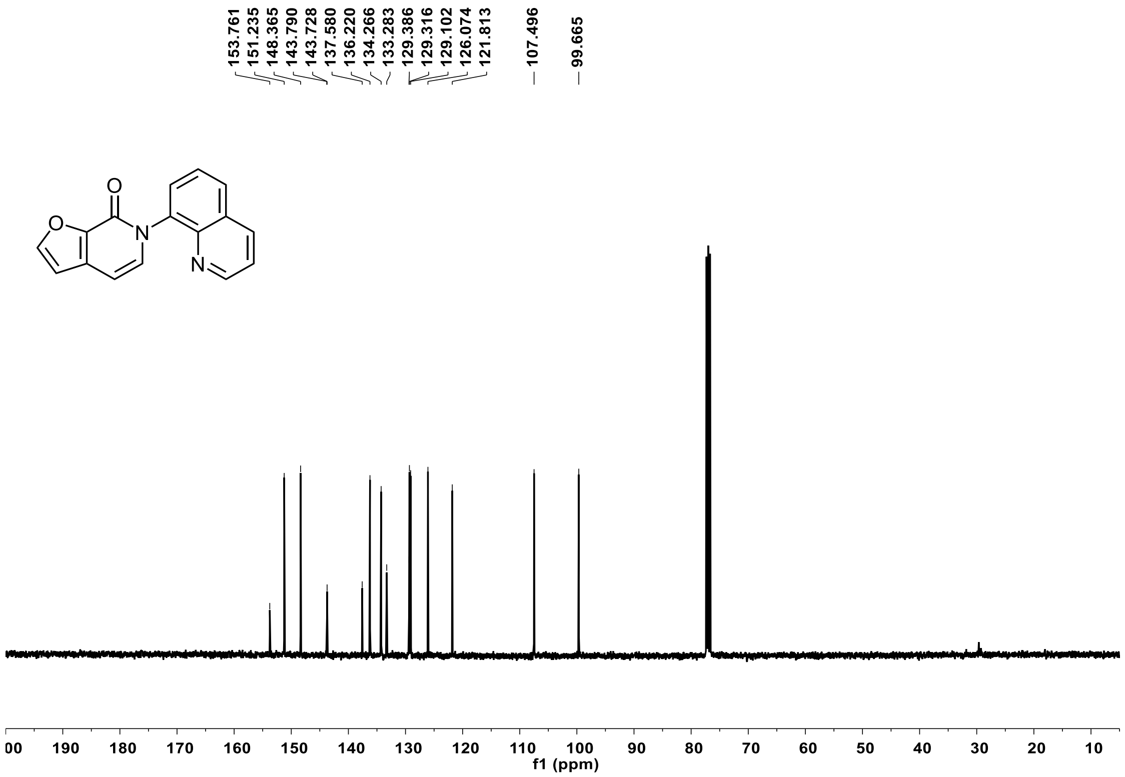

Supplementary Figure 43. ^{13}**C NMR (101 MHz, CDCl**$_{3}$**) spectrum of 3hb**

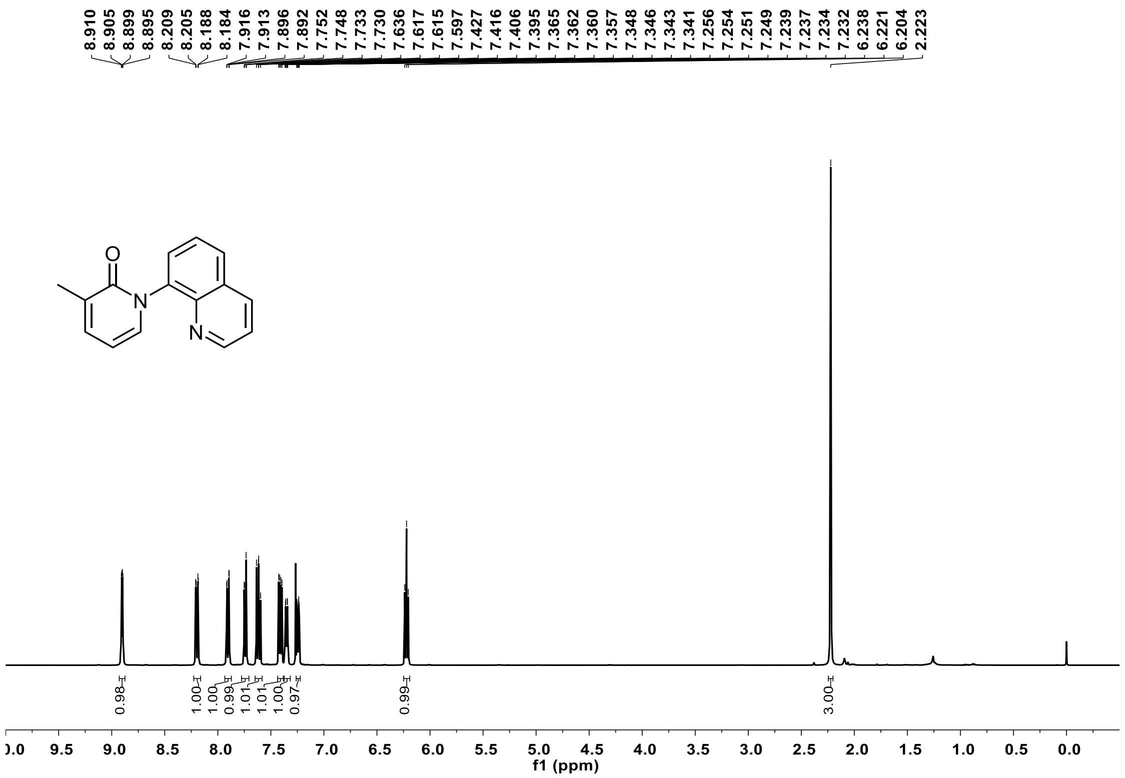

Supplementary Figure 44. ^{1}H NMR (400 MHz, $CDCl_3$) spectrum of 3ib

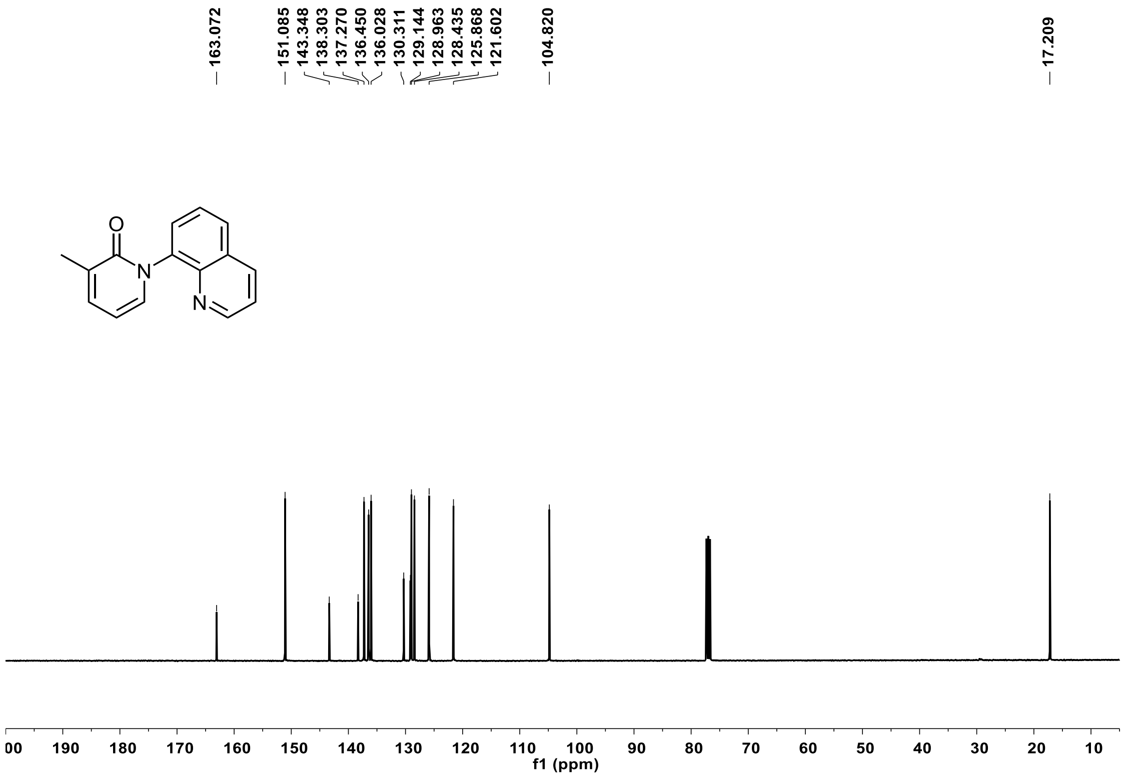

Supplementary Figure 45. ^{13}C NMR (101 MHz, $CDCl_3$) spectrum of 3ib

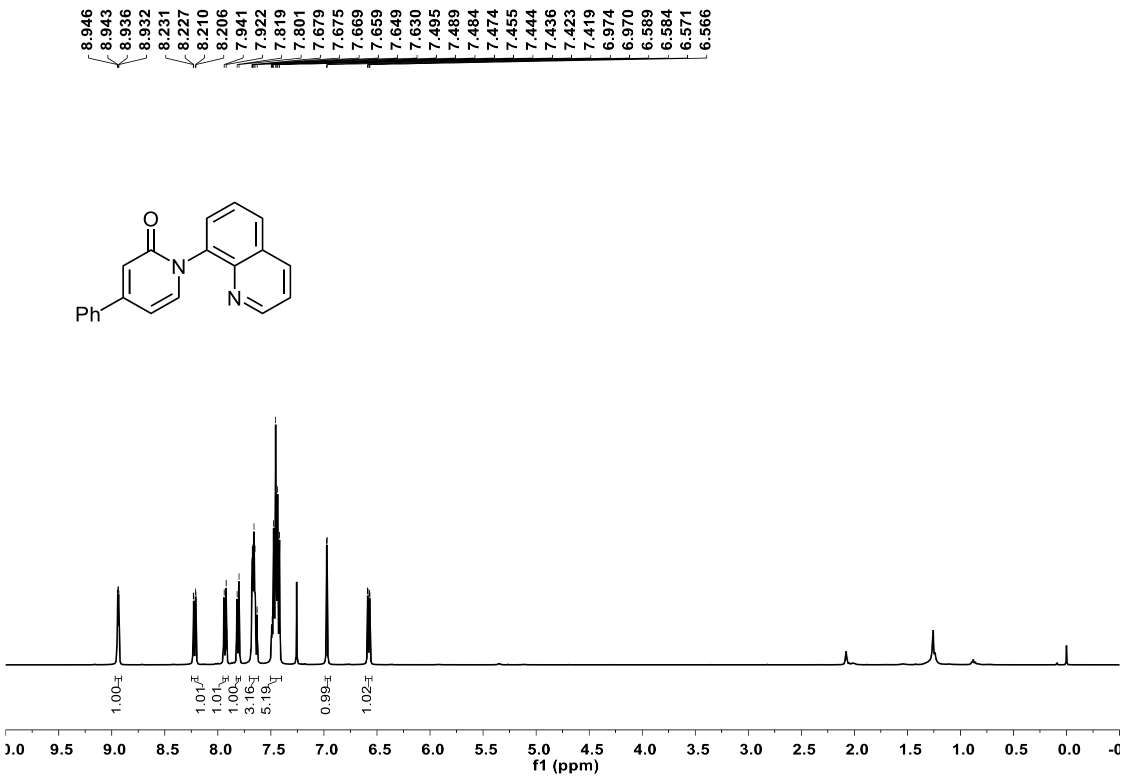

Supplementary Figure 46. ^{1}H NMR (400 MHz, $CDCl_3$) spectrum of 3jb

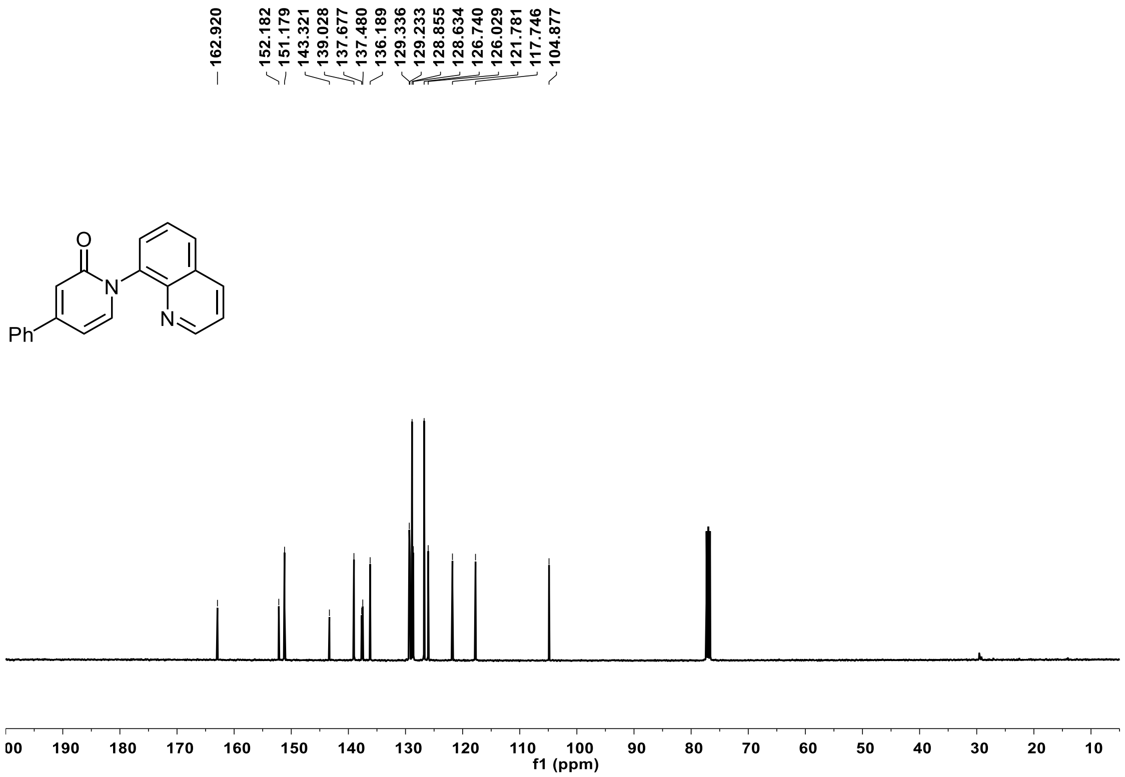

Supplementary Figure 47. ^{13}C NMR (101 MHz, $CDCl_3$) spectrum of 3jb

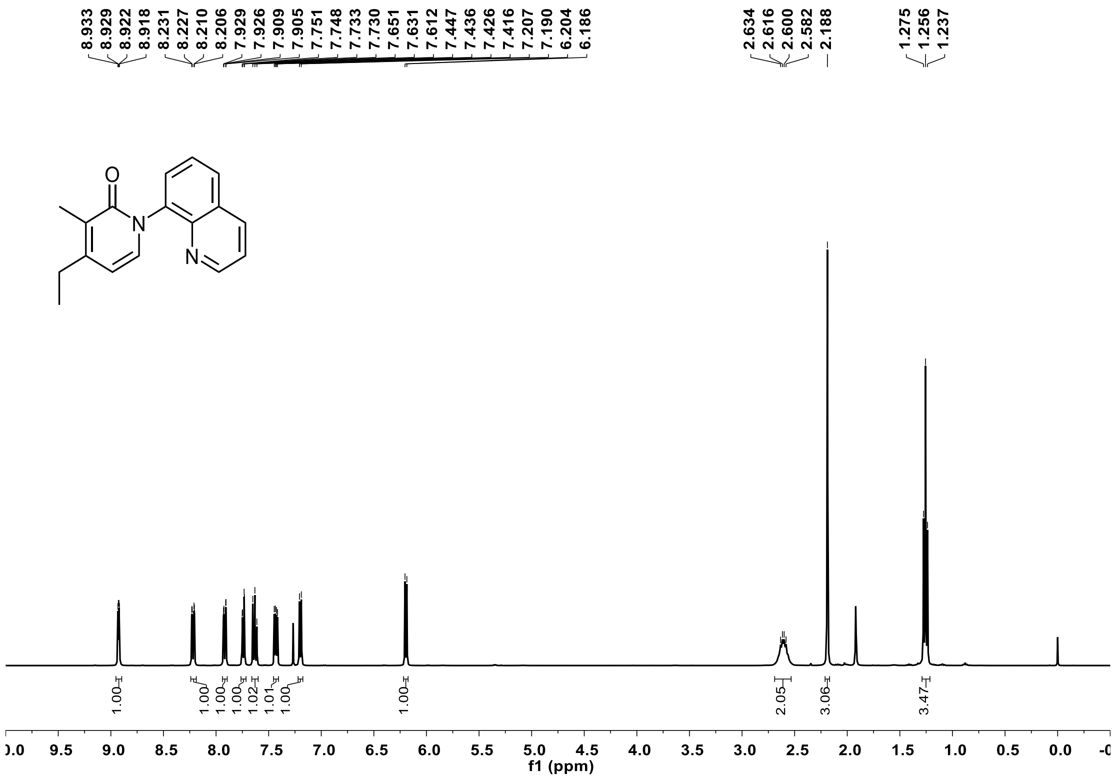

Supplementary Figure 48. ^{1}H NMR (400 MHz, $CDCl_3$) spectrum of 3kb

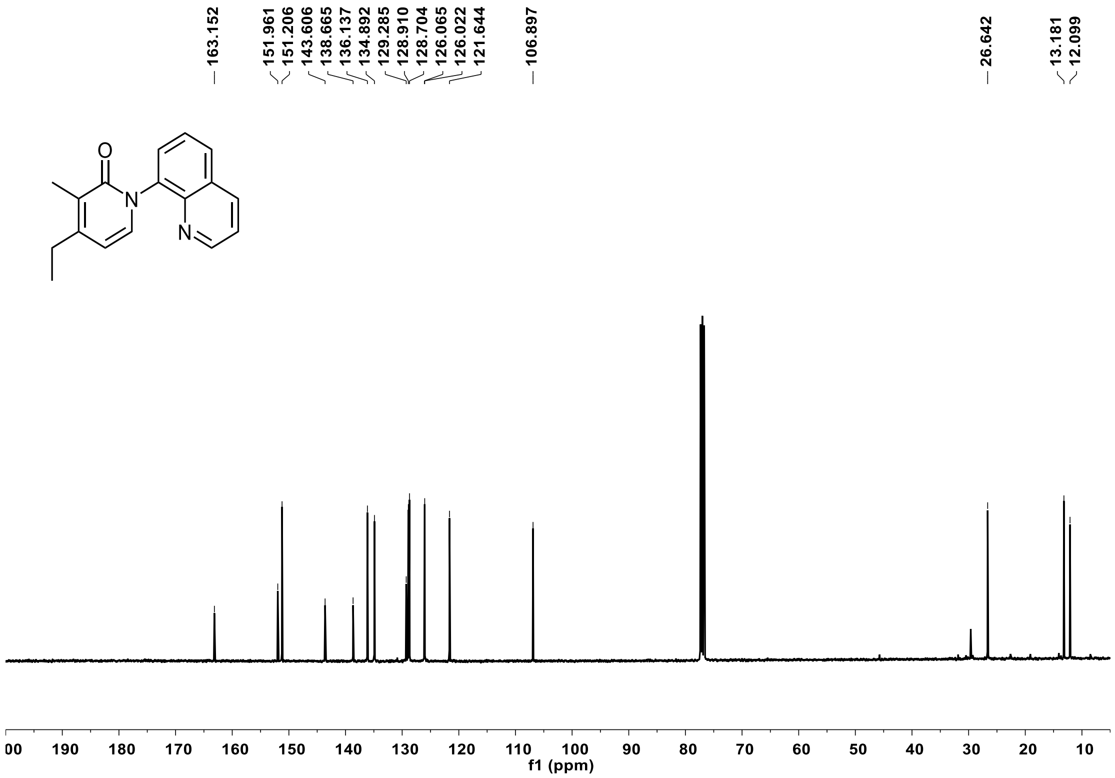

Supplementary Figure 49. ^{13}C NMR (101 MHz, $CDCl_3$) spectrum of 3kb

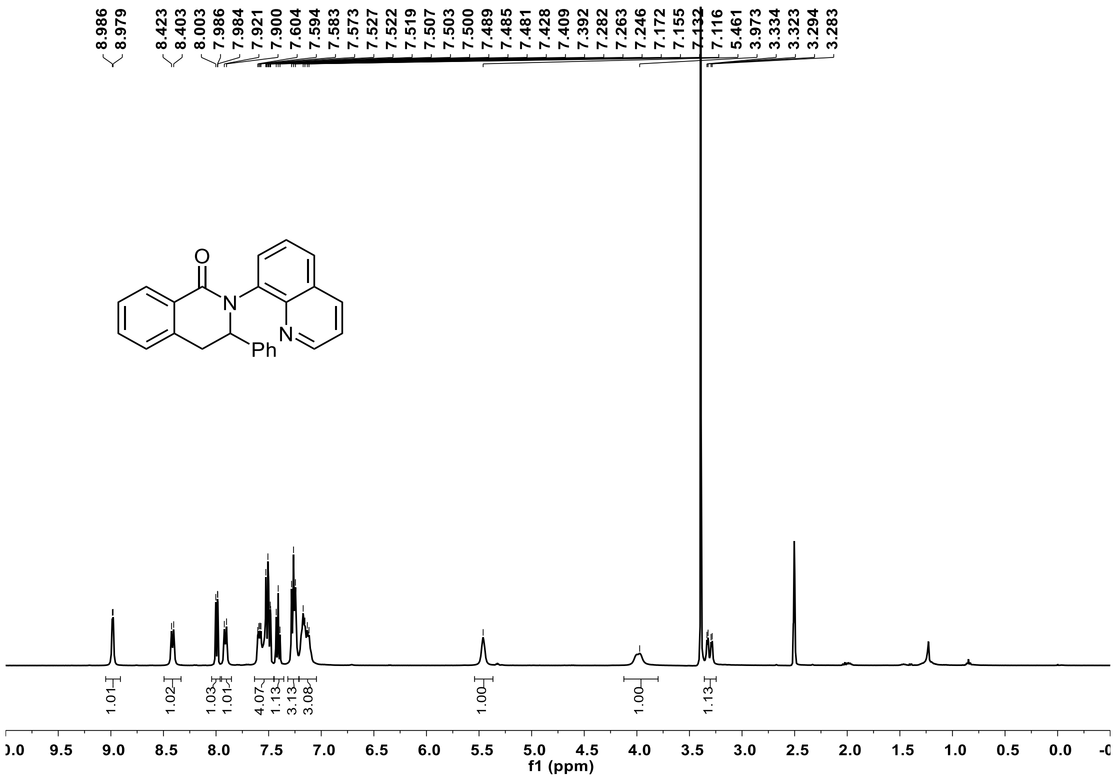

Supplementary Figure 50. ^{1}H NMR (400 MHz, DMSO-d_6) spectrum of 3ac

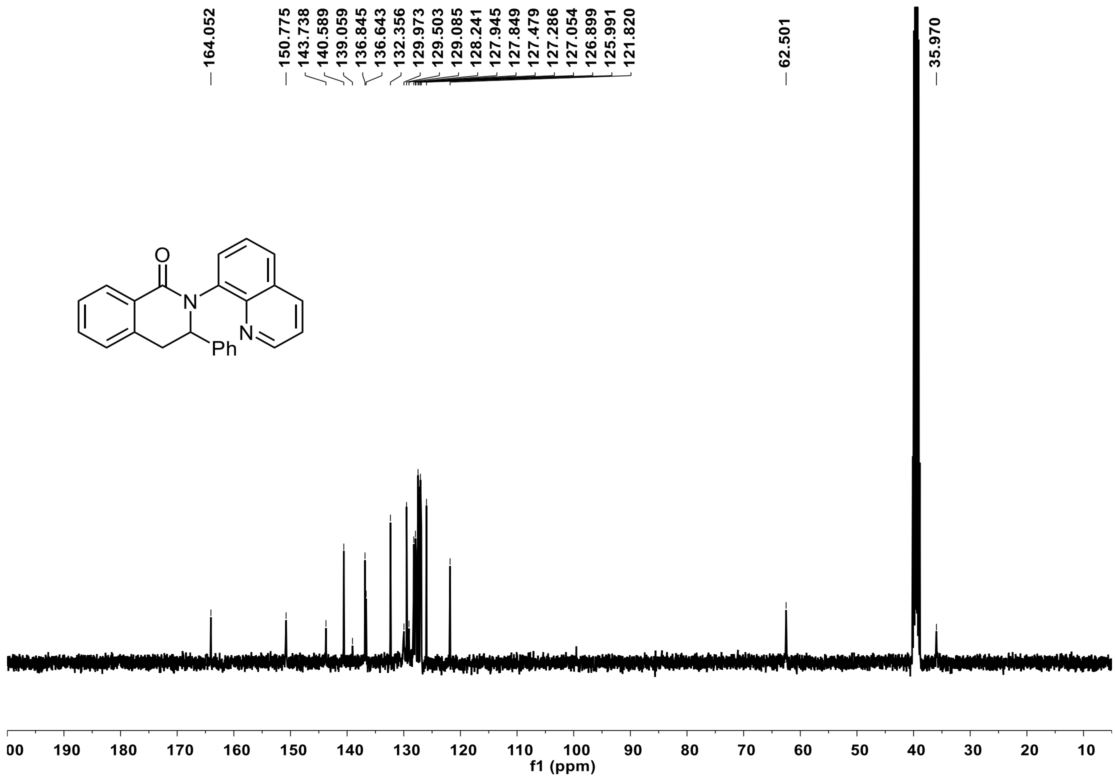

Supplementary Figure 51. ^{13}C NMR (101 MHz, DMSO-d_6) spectrum of 3ac

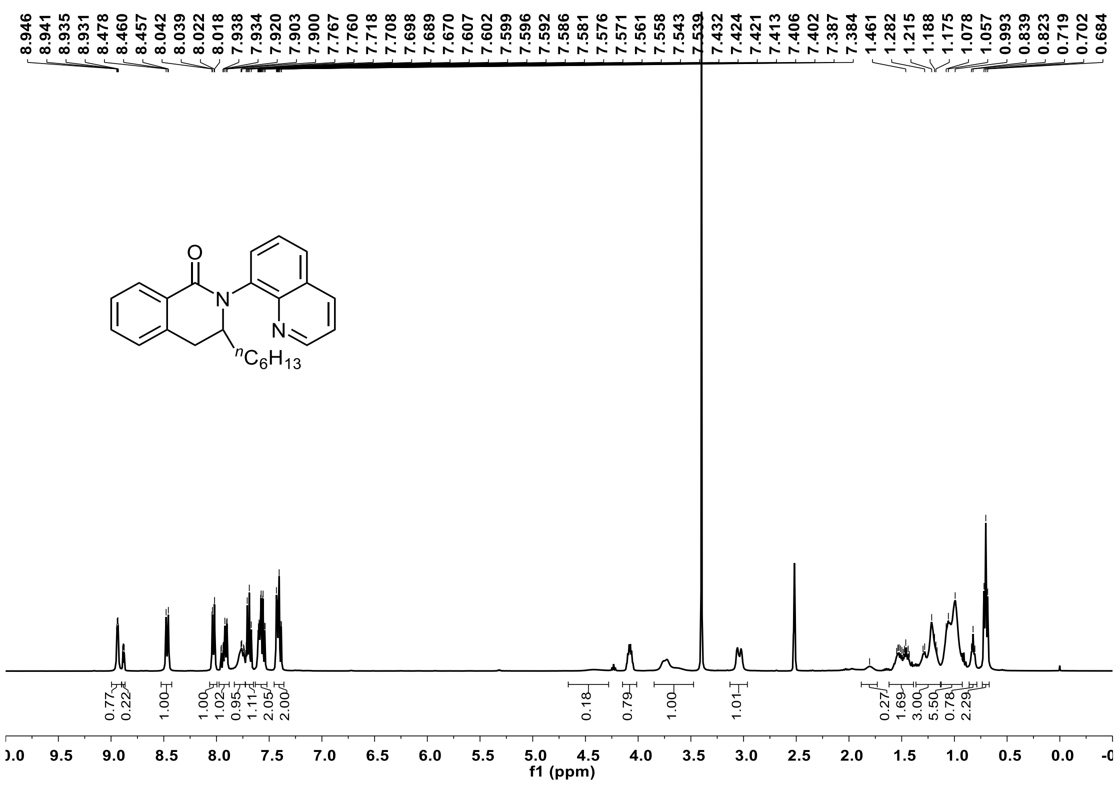

Supplementary Figure 52. ^{1}H NMR (400 MHz, DMSO-d_6) spectrum of 3ad

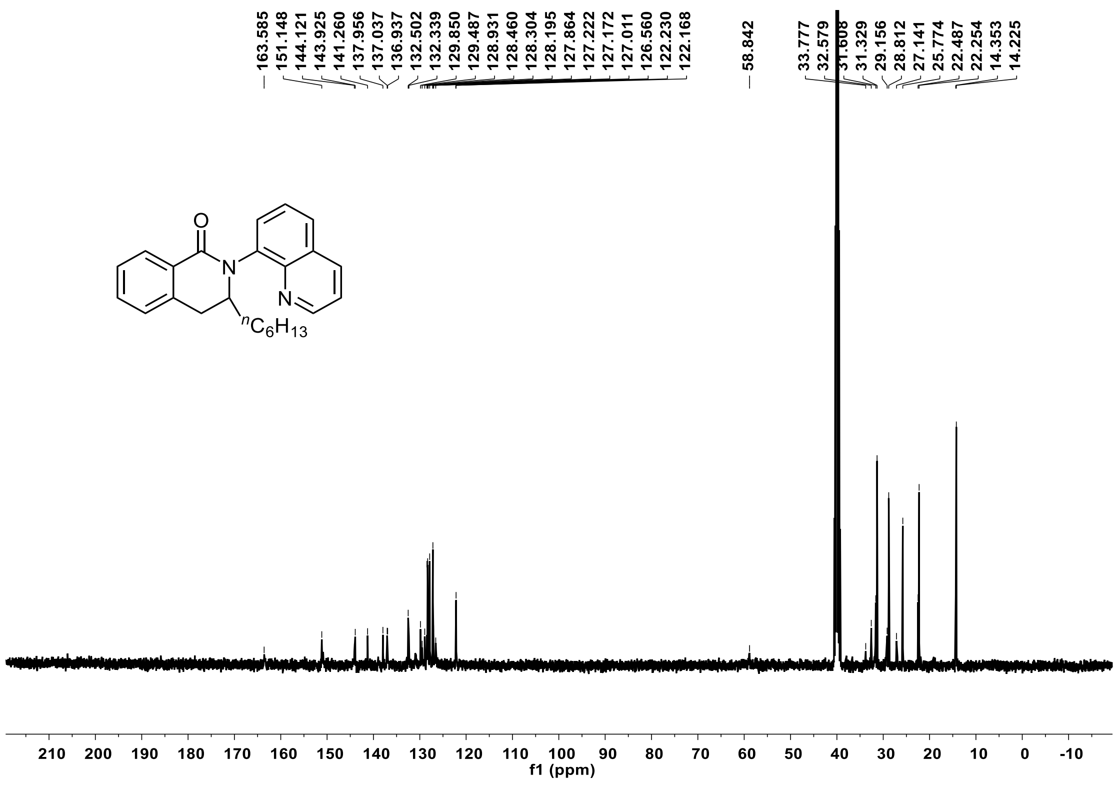

Supplementary Figure 53. ^{13}C NMR (101 MHz, DMSO-d_6) spectrum of 3ad

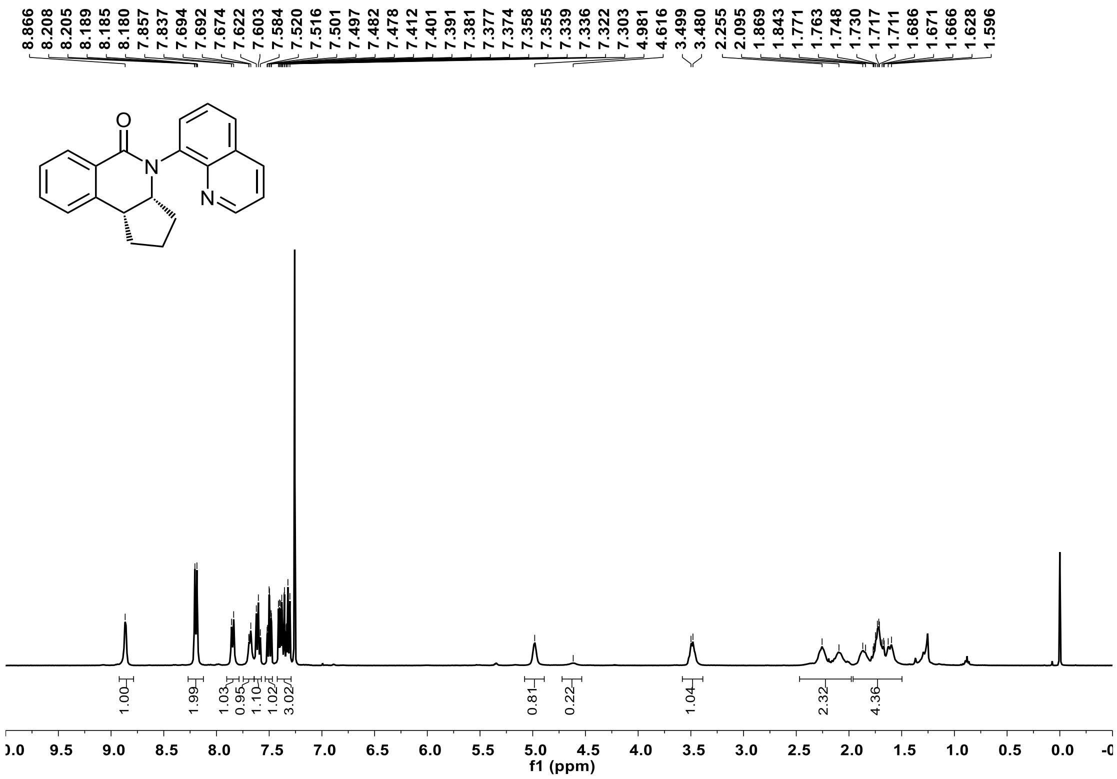

Supplementary Figure 54. ^{1}H NMR (400 MHz, $CDCl_3$) spectrum of 3ae

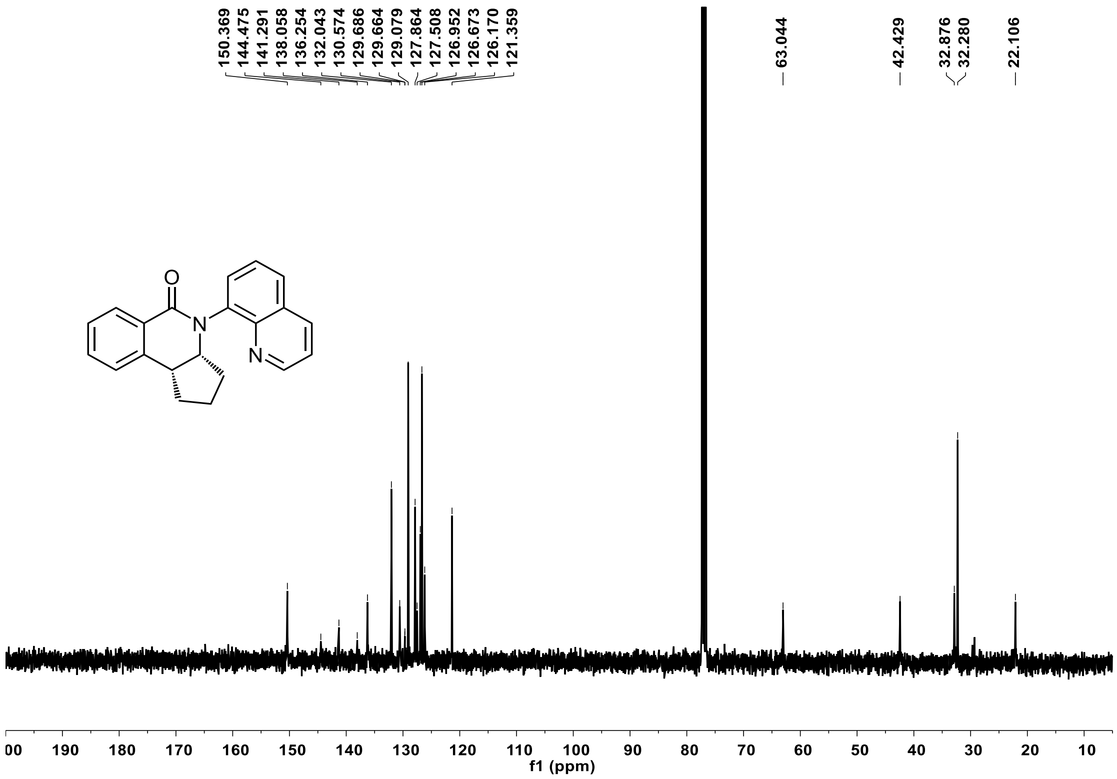

Supplementary Figure 55. ^{13}C NMR (101 MHz, $CDCl_3$) spectrum of 3ae

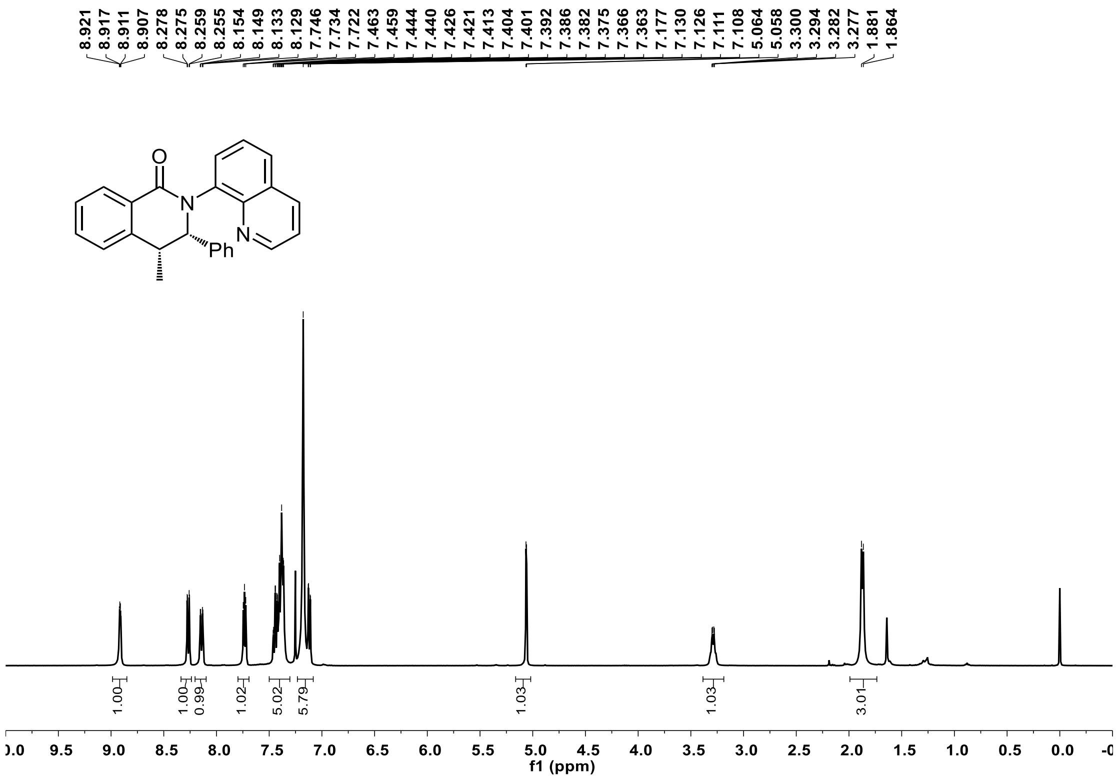

Supplementary Figure 56. ^{1}H NMR (400 MHz, $CDCl_3$) spectrum of 3af

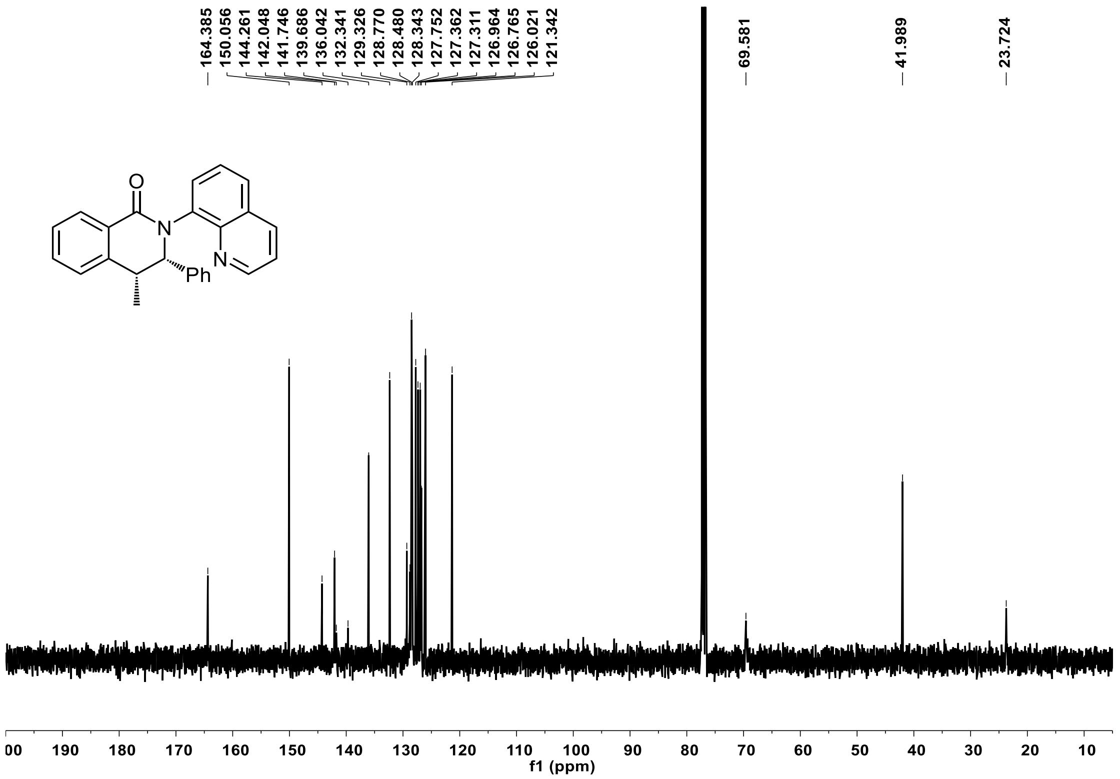

Supplementary Figure 57. ^{13}C NMR (101 MHz, $CDCl_3$) spectrum of 3af

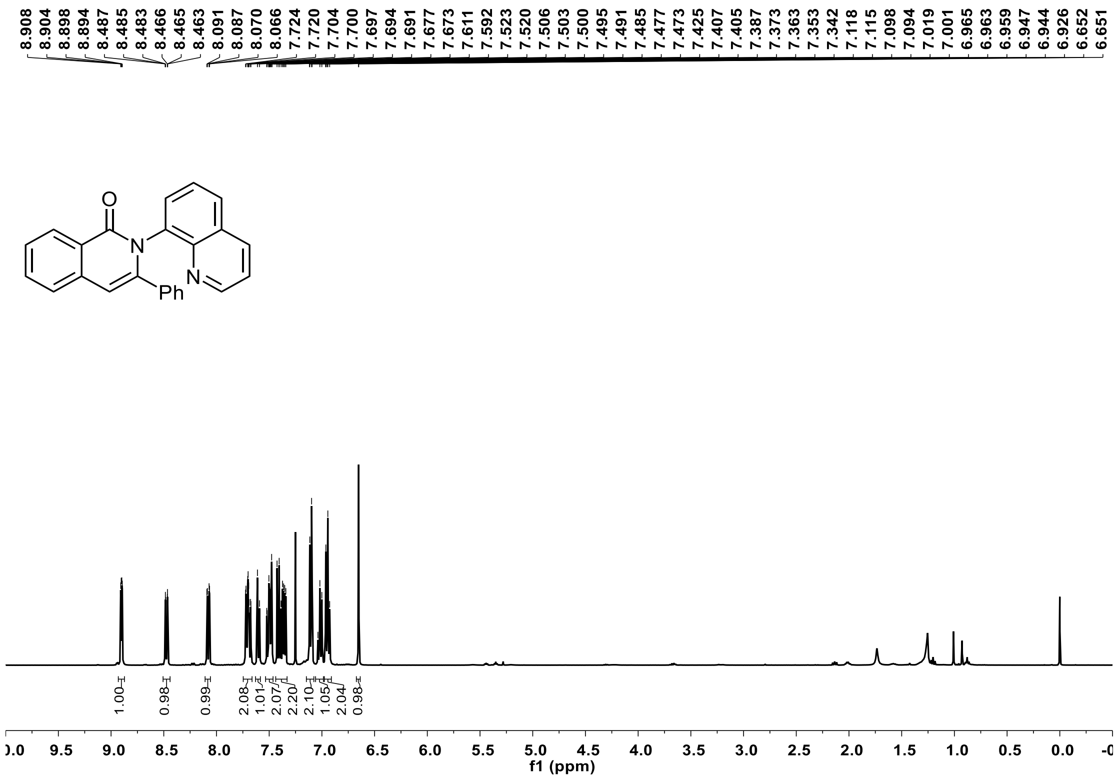

Supplementary Figure 58. ^{1}H NMR (400 MHz, $CDCl_3$) spectrum of 3ag

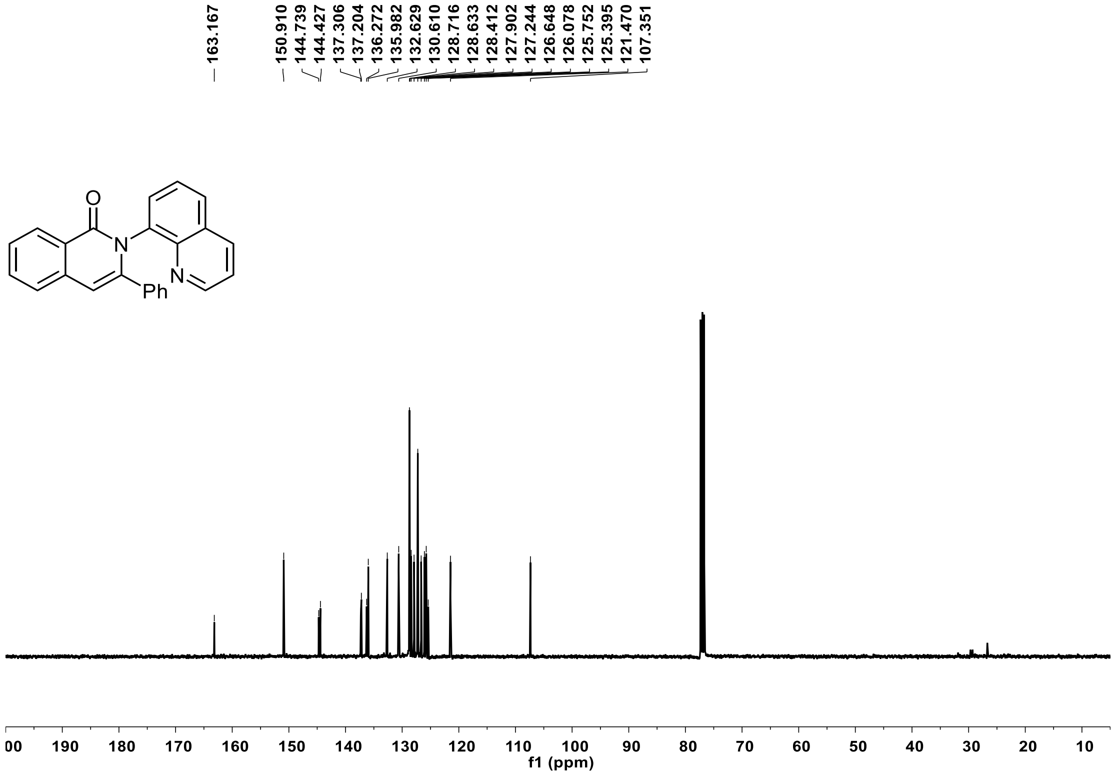

Supplementary Figure 59. ^{13}C NMR (101 MHz, $CDCl_3$) spectrum of 3ag

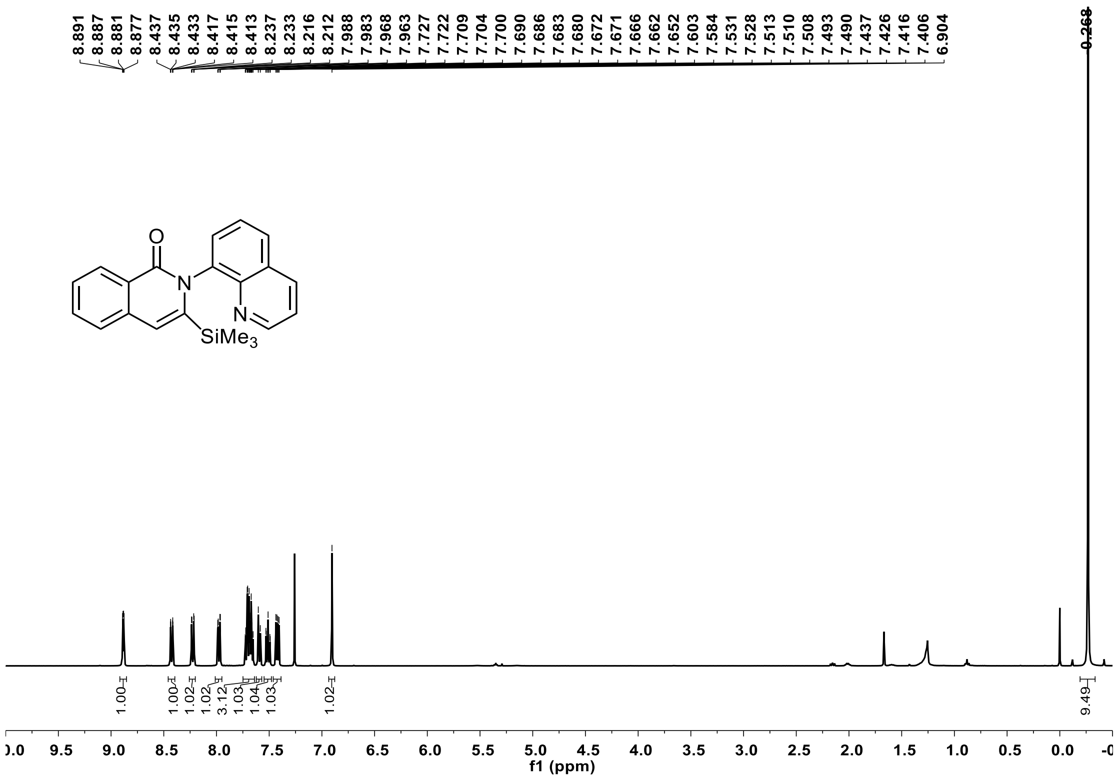

Supplementary Figure 60. ^{1}H NMR (400 MHz, $CDCl_3$) spectrum of 3ah

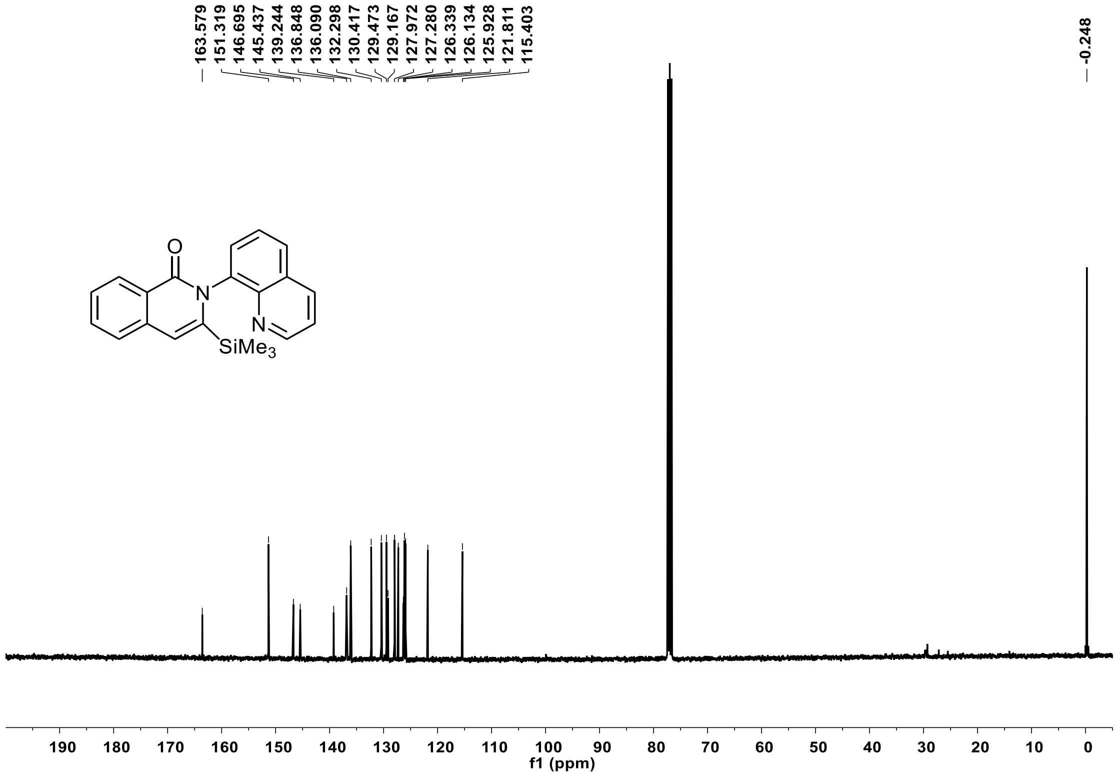

Supplementary Figure 61. ^{13}C NMR (101 MHz, $CDCl_3$) spectrum of 3ah

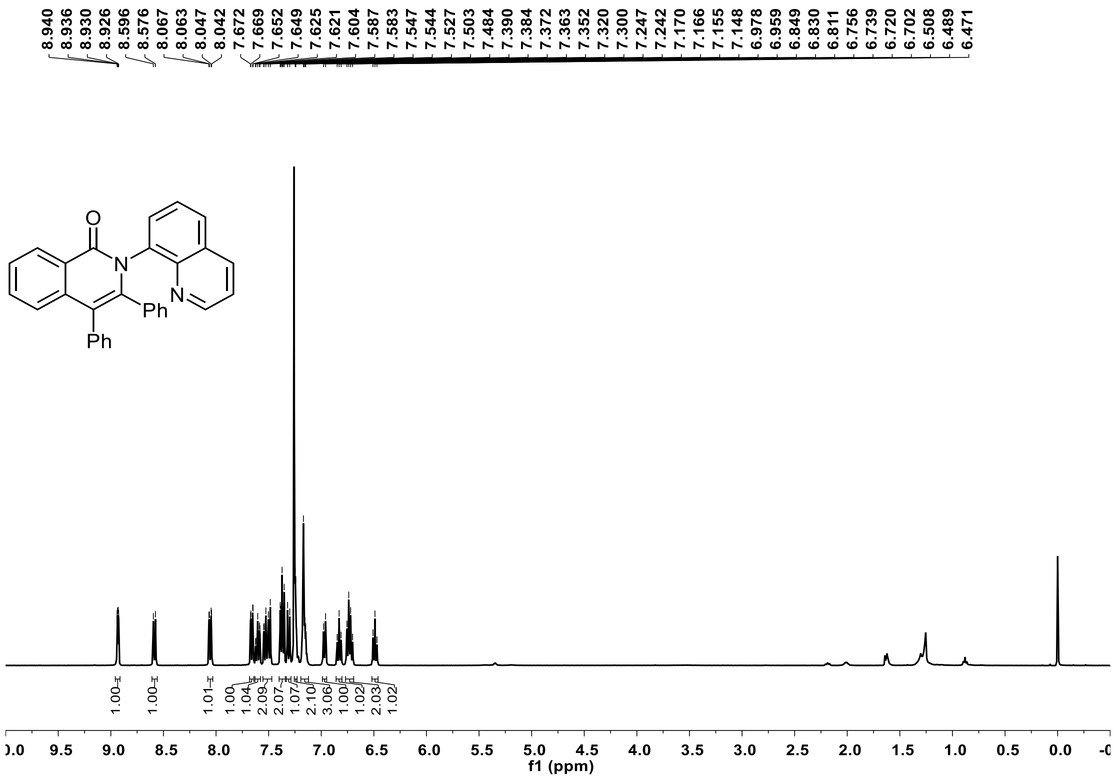

Supplementary Figure 62. ^{1}H NMR (400 MHz, $CDCl_3$) spectrum of 3ai

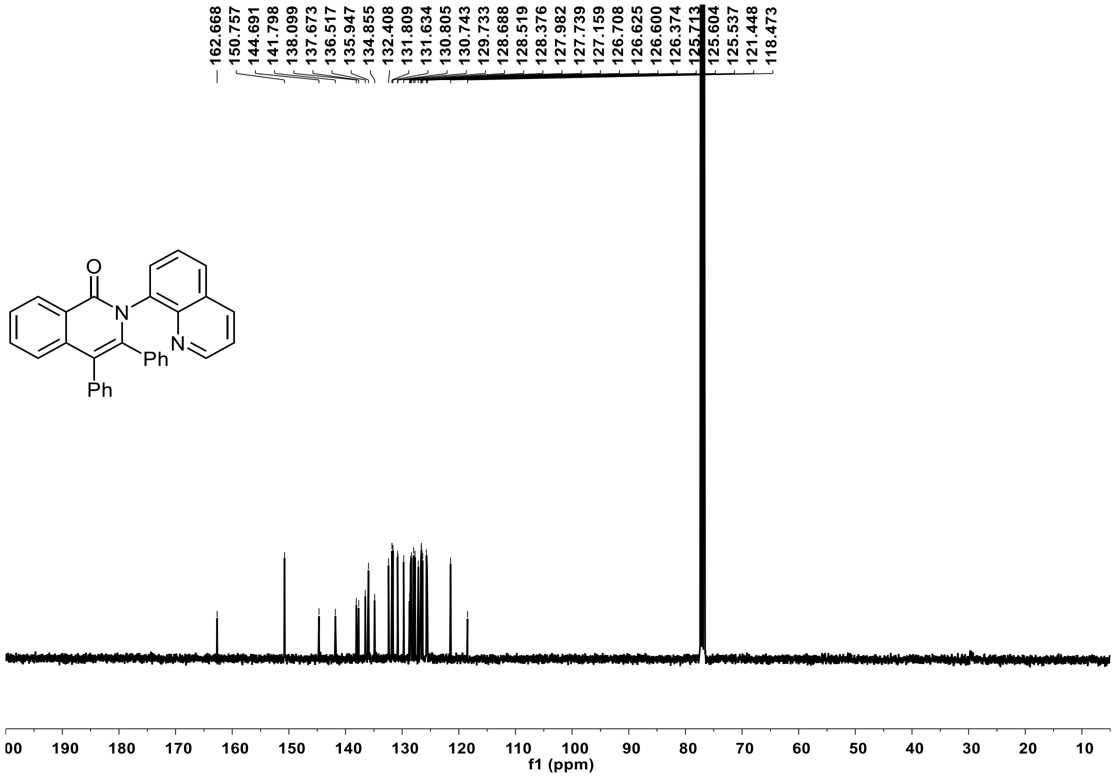

Supplementary Figure 63. ^{13}C NMR (101 MHz, $CDCl_3$) spectrum of 3ai

Supplementary References

1. Grayaznova TV, *et al.* Pyridine-directed palladium-catalyzed electrochemical phosphonation of C(sp2)–H bond. *J. Organomet. Chem.* **785**, 68-71 (2015).

2. Saito F, Aiso H, Kochi T, Kakiuchi F. Palladium-Catalyzed Regioselective Homocoupling of Arenes Using Anodic Oxidation: Formal Electrolysis of Aromatic Carbon–Hydrogen Bonds. *Organometallics* **33**, 6704-6707 (2014).

3. Grigorjeva L, Daugulis O. Cobalt-Catalyzed, Aminoquinoline-Directed Coupling of sp^2 C–H Bonds with Alkenes. *Org. Lett.* **16**, 4684-4687 (2014).

4. Gao K, Yoshikai N. Low-Valent Cobalt Catalysis: New Opportunities for C–H Functionalization. *Acc. Chem. Res.* **47**, 1208-1219 (2014).

5. Ackermann L. Cobalt-Catalyzed C–H Arylations, Benzylations, and Alkylations with Organic Electrophiles and Beyond. *J. Org. Chem.* **79**, 8948-8954 (2014).

6. Moselage M, Li J, Ackermann L. Cobalt-Catalyzed C–H Activation. *ACS Catal.* **6**, 498-525 (2016).

7. Grigorjeva L, Daugulis O. Cobalt-Catalyzed, Aminoquinoline-Directed C(sp^2)-H Bond Alkenylation by Alkynes. *Angew. Chem. Int Ed.* **53**, 10209-10212 (2014).
